# A systematic review and meta-analysis on the effectiveness of if-then plans – in a strict sense – to facilitate fruit and vegetable consumption in adults

**DOI:** 10.1186/s12966-026-01915-y

**Published:** 2026-04-15

**Authors:** Sanne Karlsen Melum, Torsten Martiny-Huenger

**Affiliations:** UiT The Arctic University of Norway, Hansine Hansens veg 18, Tromsø, N-9019 Norway

**Keywords:** Healthy diet, Intervention, If-then planning, Meta-analysis

## Abstract

**Background:**

A fruit-and-vegetable rich diet is important in the prevention of non-communicable diseases. If-then plans, or implementation intentions, are proposed to facilitate behavior change by formulating plans that link perceivable cues and goal-directed responses. We investigated the effectiveness of if-then planning interventions to facilitate fruit and vegetable intake in adults, with a strict focus on if-then planning procedures and excluded procedures representing conventional planning.

**Methods:**

A systematic review (MEDLINE, Embase, and PsycInfo; last searched April 3rd, 2025) and meta-analysis was conducted. Included studies were randomized controlled trials, testing the effect of if-then plans on fruit and/or vegetable intake against active control groups.

**Results:**

Ten articles were identified as eligible, including 12 comparisons (*N* = 2399) with intervention-outcome periods of 1 week to 24 months. If-then planning participants reported consuming approximately a quarter of a portion of fruit and vegetable per day more than participants in the control groups (*MD* = 0.29, 95% *CI*: 0.11; 0.48).

**Conclusion:**

A central limitation of the analysis is that all included studies are based on self-reported fruit and vegetable consumption. While the effect size of the investigated intervention is small, its low resource requirements make it an accessible option for promoting fruit and vegetable consumption.

**Supplementary Information:**

The online version contains supplementary material available at 10.1186/s12966-026-01915-y.

## Background

Non-communicable diseases (e.g., cardiovascular disease, cancer, type 2 diabetes mellitus) and their associated risk factors (e.g., overweight/obesity, high blood pressure, high blood glucose) are prevalent, causing high rates of morbidity and mortality globally [1, 2]. A healthy diet rich in fruit and vegetables is important in the prevention and management of non-communicable diseases [3]. Fruit and vegetables are low in energy, high in fiber, and are a good source of a variety of micronutrients and antioxidants that are associated with a lower risk of many non-communicable diseases [4]. The current recommendation for fruit and vegetable intake from the World Health Organization (WHO) is to eat at least five portions per day (i.e., 1 portion ~ 80 g) [5, 6]. Estimates of actual fruit and vegetable consumption in the European Union (EU), along with specific data from Germany and Norway, indicate that 20–30% of EU adults do not consume fruit and vegetables on a daily basis [7, 8]. Moreover, only 14–30% meet the recommended intake of five portions per day [8, 9]. Furthermore, data from Germany suggest that these numbers have not changed substantially over the last decade [7]. While an unhealthy diet is a modifiable risk factor, it is not easy to make sustainable and lasting dietary changes [10], and health care providers, including registered dietitians need evidence-based, resource-efficient and effective strategies to guide and support patients to increase their fruit and vegetable intake.

### Behavior-change theories and strategies in dietetics

A central role of the dietitian (or health-care provider) is to support clients (the public) in implementing nutrition-related recommendations. In nutrition and dietetics there has been an emphasis on targeting motivational aspects of eating behavior (e.g., motivational interviewing, S.M.A.R.T goals) [11–13]. This focus overlaps with a long tradition in psychological research of emphasizing the relevance of motivation and goal setting [14–16]. Nonetheless, investigations into the intention-behavior relations indicate that goal setting (motivation) is often not enough to lead to the intended behaviors [10, 17, 18]. In addition, strategies that facilitate implementing intended behaviors (volition) in everyday situations are important [19]. The present meta-analysis is focused on a self-regulation strategy specifically targeting the translation of intentions into behavior: if-then planning (*implementation intentions*) [19, 20]. Because both terms, *if-then planning* and *implementation intentions*, are used interchangeably in the literature, we will henceforth refer to the strategy as if-then planning. The strategy’s potential to support behavior change, while at the same time being resource-efficient, makes it a primary candidate to be adopted in a wide range of applied health interventions.

### Why is if-then planning a promising strategy

Every day, people execute specific behavior to collect (e.g., buy, pick one item over another), prepare (e.g., cook, fill a lunch box, add sugar or salt), and consume (e.g., eat, get a refill) food. While behavior is initially driven by deliberately searching through options and choosing based on preferences [21], over time and repetition, habits start guiding the daily routines without much further deliberation in the specific situations [22]. Motivational approaches to overcoming habitual behavior face two problems: First, newly set goals do not immediately change the previously established habit structures that continue to drive old behaviors [23]. Second, motivational arousal at the time of goal setting (e.g., during a nutrition counseling session) is likely to fade amid subsequent everyday concerns and is not spontaneously reinstated at critical moments, hours or days later [24].

If-then planning addresses both of these problems [25]. The strategy involves identifying a critical situation (i.e., a perceivable cue, such as feeling hungry in the office) and a directly actionable, goal-directed response (e.g., heading to the grocery store to buy a salad). Both parts are then combined to form a situation-response plan (e.g., “If I feel hungry in the office, then I will head to the grocery store and buy a salad”).

With regard to the previously identified problems: First, formulating and memorizing such if(situation)-then(response) plans is assumed to change a person’s memory structures in a way that adds new habit-like, goal-congruent situation-response links. Second, because the goal-directed response is linked to a perceivable cue, the memory structure is activated in a bottom-up fashion as soon as the critical cue is perceived [26, 27]. Consequently, if-then planning is assumed to create new, goal-congruent situation-response links that establish the required bottom-up automaticity to change even habitual behaviors [28] such as everyday food consumption.

### Ambiguities in the “if-then planning” label

Interventions labeled as if-then planning have not always adhered to the previously described characteristics that define the strategy [29]. Responding to this issue, Hagger and Luszczynska [29] identified “if-then planning” studies that implemented a form of “when, where, and how” planning but argued that if-then planning and “when, where, and how” planning are two distinct types of action planning [29]. If-then plans include (A) a perceivable cue, (B) a directly actionable, goal-directed response, and (C) a clear link between the two within the plan. In contrast, “when, where, and how” plans to achieve a goal and other forms of planning (e.g., calendar-like, time-based planning, such as “going for a run on Thursday at 6pm”) are missing central aspects of these characteristics: First, such planning often does not include clear perceivable cues that can serve as anchors for the goal-directed response. Second, the goal-directed responses are often not directly actionable and require additional preparatory actions, which are themselves subject to forgetting.

Besides Hagger and Luszczynska’s [29] arguments on this topic (see also Vilà ([30] p282)), central aspects of the present reasoning can be found in other areas. For example, the relevance of perceivable cues for “remembering” and triggering linked responses has been highlighted in research on prospective memory [31, 32] and habit formation ([33] p184). In conclusion, if-then planning is assumed to work through a set of specific mechanisms. For these mechanisms to function effectively, the intervention procedure must include the previously specified components. Consequently, to estimate the effectiveness of if-then planning in this strict sense, we included only studies with intervention procedures that incorporated the critical characteristics of if-then planning from the research labeled as employing “implementation intentions” or ”if-then planning.” Studies in which any one of these aspects was missing were excluded.

### Prior meta-analyses of if-then planning

A database search and a search on PROSPERO [34] did not identify reviews or meta-analyses on the specific topic of if-then planning and fruit and vegetable consumption. However, three meta-analyses have investigated if-then planning with a healthy diet as the outcome. Importantly, all three included subgroup analyses on promoting a healthy diet, which are closely related to the present topic of promoting fruit-and-vegetable intake.

The earliest meta-analysis reported a positive effect of the planning intervention, with a Cohen’s d of 0.51 [35]. However, the authors cautioned that this effect might be an overestimation due to the inclusion of studies with suboptimal control groups ([35] p192). Subsequent meta-analyses appear to support this caution, reporting positive but smaller effect sizes of 0.33 [36], and 0.26 [37]. The latter study even reported a very small effect size of 0.12 after correcting for publication bias ([37] p138)]. Importantly, these meta-analyses included studies that should be categorized as alternative forms of planning, rather than if-then planning in a strict sense. Thus, the reported effect-size estimates are not specific to if-then planning.

Additionally, we identified two meta-analyses [30, 38] that report effect-size estimates for if-then planning subgroups that exclude other types of planning. One recent large-scale analysis of all if-then planning studies (642 comparisons) [38] found an effect size for the if-then planning subgroup of 0.43 (373 comparisons). However, decisions on including a study as “if-then planning” were based on the ability to retrieve the content of the plans that participants formed ([38] p167). Unfortunately, this categorization leans heavily toward including laboratory studies where researchers provided participants with a specific if-then plan and excluded the type of intervention studies that are our present focus. Sheeran et al.’s [38] approach likely resulted in a narrow selection of clear if-then planning implementations such as McCrea et al. [39] However, if this categorization, almost by default, excludes most applied intervention studies that require participants to form their own if-then plans (which are then not listed in the article), the method is not suitable for our current goal of identifying applied intervention studies that adhere as closely as possible to if-then planning in a strict sense, and to estimate their effect size.

We identified one other meta-analysis targeting applied interventions on reducing unhealthy fat consumption, which reported a subgroup analysis of if-then planning with alternative kinds of planning excluded [30]. This analysis showed an effect size of 0.32. However, the analysis identified and included only three studies and should thus be considered as providing only preliminary evidence.

### The present research

The objective of the present systematic review and meta-analysis is to evaluate the effectiveness of the self-regulation strategy if-then planning, or implementation intentions - in a strict sense - to increase adults’ fruit and vegetable consumption. The rationale behind this objective is that fruit and vegetables are highly health-beneficial food that is consumed too infrequently by a majority of the world’s population [3, 4, 7–9]. The focus on if-then planning is because it is a theory-based strategy [19, 20] that can be implemented resource-efficiently, for example, in typical nutrition counseling sessions. The focus of the present meta-analysis on if-then planning in a strict sense is warranted to avoid confounding the effectiveness evaluation with action planning in a broader, more conventional sense.

## Method

This systematic review and meta-analysis was conducted and reported following The Preferred Reporting Items for Systematic Reviews and Meta-Analyses (PRISMA [40]). No meta-analysis protocol was submitted to an official registry. However, an unpublished protocol that guided the present meta-analysis can be accessed in the Appendix of Melum [41]. The eligibility criteria for inclusion in the meta-analysis are summarized in Table 1.

**Table 1:** List of eligibility criteria

Factor	Description
Study design	Randomized control trial (RCT)
Population	Adults (age > 18); no psychological diagnosis
Intervention	If-then planning intervention in an if (situation)-then (response) format.
Control	Active control group that encouraged the same behavior change as the intervention but did not provide guidance to form if (situation)-then (response) plans.
Outcome	Fruit and/or vegetable intake in participants’ everyday environment (e.g., retrospective self-report).
Other criteria	Peer-reviewed journal articles published in English (MEDLINE, Embase, and APA PsycInfo).

More detailed information on the selection of if-then planning interventions is presented in Table 2

### Information sources and search strategy

The literature search was conducted in three electronic databases: MEDLINE, Embase, and APA PsycInfo on the 23rd of November, 2022, without date restrictions (an updated search on 03. April. 2025 showed no additional published studies that met the inclusion criteria). Additional non-database search included hand searching reference lists of published reviews and reference lists of the included journal articles. In essence, the literature search on ‘abstract’, ‘title’ and ‘keywords’ consisted of the following elements: ‘intervention’ (i.e., if-then plans OR implementation intentions) AND ‘outcome’ (e.g., fruit OR vegetable OR food; all details are listed in Appendix 1). Additionally, we aimed to identify unpublished studies by sending out a request for unpublished data via the mailing list of the European Society of Social Psychology and by searching preregistrations on the Open Science Framework [42]. No unpublished data were identified, and no preregistrations were identified that pointed to the existence of unpublished data.

### Study selection procedure

First, duplicates were removed from the initially retrieved references. The remaining references were imported to Covidence [43], where they were screened by title and abstract for eligibility by SKM. Study reports found eligible based on their title and abstract were assessed in full-text by both SKM and TMH. Those that met the inclusion criteria were further evaluated to determine whether the intervention procedure adhered to if-then planning in a strict sense. This evaluation was conducted independently by SKM and TMH based on the information provided in the reports and the original instructions in cases where the report authors complied with our request to share them (see Appendix 2 for details). Uncertain cases were resolved by discussion between SKM and TMH.

### Outcome prioritisation and comparison selection

The outcome of interest was fruit and/or vegetable intake. We prioritised portions per day if several outcome measures were reported, and converted alternative measures (e.g., per week measures) to portions per day if feasible. Statistical comparisons were performed on the main effect between intervention condition and control condition at the latest reported outcome measure. Differences in the studies were dealt with and prioritised as follows: (1) We prioritised fruit and vegetable intake reported as a summarized measure; (2) we combined fruit and vegetable intake when reported separately; (3) we combined different intervention conditions when the study included only a single control condition (details are reported in the Data Adjustment section) and the intervention conditions applied if-then planning in a strict sense. Data extraction was first performed by SKM and subsequently validated by TMH. Deviating outcomes were resolved through discussion between the two authors.

### Data adjustments

Some articles required data adjustments to fully utilize the available data. The details of these adjustments are described in Appendix 3. One report [44] included two intervention conditions but only one control group, and fruit and vegetable consumption was reported as separate measures. To make full use of the data, we combined the fruit and vegetable measures and the two intervention conditions to compare them with the single control condition. For another report [45], per-day fruit-and-vegetable scores were calculated from the reported per-week scores. Standard deviations were estimated from the baseline (numerically reported) and the result plots. In three comparisons (from two reports [46, 47]), we observed irregularities in the reported standard deviation, which led us to suspect that the reported values were mislabeled standard errors (see Appendix 5, for the meta-analysis results without the transformation). We transformed the values suspected to be standard errors into standard deviations. Finally, we made no adjustments to the “cup” measure in one report [48] and treated one cup as equal to one portion, consistent with the other studies.

### Statistical analyses

A meta-analysis using an inverse-variance random-effects model with restricted maximum likelihood (REML) estimator for heterogeneity (between study variance) [49], and Hartung-Knapp-Sidik-Jonkman (HKSJ) adjustment [50, 51] was performed in R [52] using the meta package [53] on the unstandardized mean difference (MD) [54]. Results from the meta-analyses are presented in a forest plot, including the prediction interval, and a contour-enhanced funnel plot. Heterogeneity was visually inspected and tested statistically using Chi² and Tau². In addition, I²-statistics was used as a guide on the inconsistency between studies, with values of ~ 25%, ~ 50%, and ~ 75% being interpreted as ‘low’, ‘moderate’, and ‘high’ inconsistency, respectively [55].

### Study quality assessment (risk of bias)

Risk of bias was assessed using the *Revised Cochrane Risk-of-Bias Tool for Randomized Trials* (RoB2 [56]), across five domains for the central intervention-control comparison in each included article. An overall risk-of-bias conclusion was reached. The analysis was conducted by SKM, and ambiguous decisions were resolved through discussion with TMH.

## Results

### Search results

**Fig. 1:**
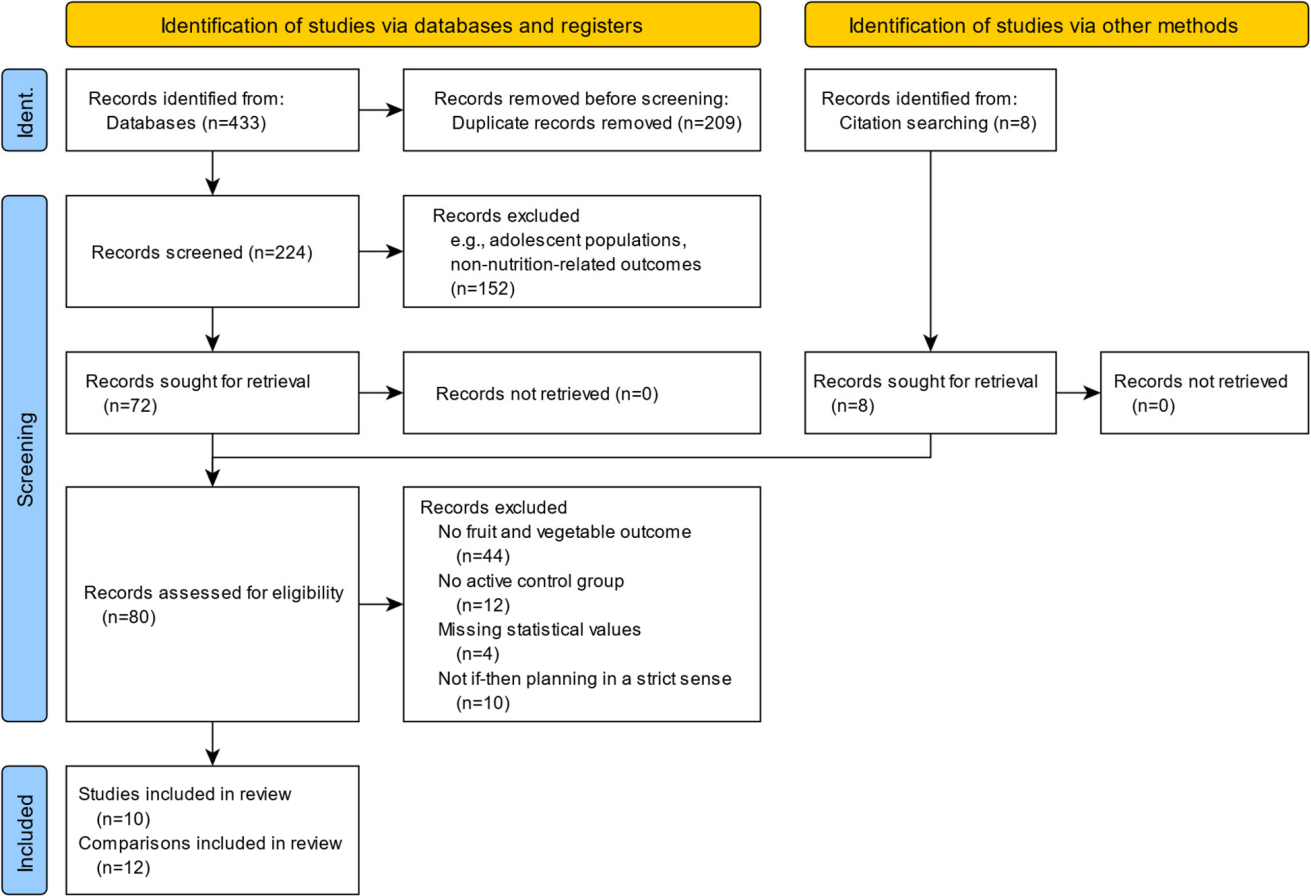
Record identification flow chart. *Note*. Ident. = Identification

Searching the databases MEDLINE, Embase and PsycInfo on if-then planning interventions and nutrition-related outcomes identified 433 records (Fig. 1 illustrates a flow chart diagram of the record identification process). Two hundred nine records were identified as duplicates and removed. The resulting 224 records were screened by title and abstract. Of those, 152 records were excluded for not meeting eligibility criteria (including children or adolescent populations, protocols, non-randomized trials, or non-nutrition-related outcomes). Additionally, 8 articles were identified in previous meta-analyses as potentially relevant and included in the subsequent full-text analysis. Thus, 80 articles were assessed in full-text. Forty-four articles were excluded for having nutrition-based outcomes other than fruit and vegetables (e.g., number of vegetarian days, fat, salt, or caloric intake). Twelve articles were excluded for not having an appropriate control group (e.g., no control group, passive control group). Four articles did not provide sufficient information required for the meta-analytic test (e.g., no means and variance information for the main effect of interest). Finally, ten articles were removed because the intervention procedure resembled action planning in general rather than strict if-then planning (Table 2 provides instruction examples and the inclusion/exclusion reasoning, and Appendix 4 presents these details for all studies in/excluded based on the intervention instructions). Consequently, a total of ten articles were identified as relevant for the meta-analysis. Three articles included two intervention conditions, and two of these allowed two independent intervention-control comparisons. Thus, a total of twelve comparisons are included in the meta-analysis (details are provided in Table 3). A table including all 70 excluded article references and the central reason for exclusion is provided in Appendix 7.

**Table 2:** Example instructions and comments on the inclusion versus exclusion reasoning

Intervention instructions	Comments
Included Study/Condition Examples	
“[…] containing lists of ten critical situations and ten appropriate responses. […] temptation items were translated into “if” statements […], the processes of change items were translated into “then” statements […]. Participants in the volitional help sheet condition […] were then asked to draw links between as many critical situations and appropriate responses as they wanted and thereby form implementation intentions […] (Armitage [46], “volitional help sheet implementation intention” condition, p. 602)	Situation: temptation cues (perceivable cues) Response: responses to temptation (goal-directed responses) Link: explicit instructions to link situations and responses
“[…] paying particular attention to the situations in which you will implement these plans. You could choose to focus on planning what fruit and vegetables to buy, how you will prepare them […] you may find it useful to state, “If it is lunchtime at university, then I will eat an apple instead of crisps!” Please write your plans in the space provided, following the format in the example (“if … then …”). […] (Chapman et al. [57], “if-then implementation intention” condition, p. 321)	Situation: Crisps at lunch (example, perceivable cue) Response: what F/V to buy, how to prepare (goal-directed responses) Link: final plan written in if-then format
“[…] a table containing a list of potential barriers to FV consumption on one side and a list of potential solutions on the other side. […] The questionnaire contained 12 barriers and one space to write down a barrier not already mentioned in the table. Each barrier was preceded by ‘if’. […] The questionnaire also contained 12 solutions and one space to write down a new solution to overcome barriers to FV consumption. Each solution was preceded by ‘then’. […] Participants were instructed to link each barrier that personally applied to them to a solution to overcome it (e.g. If I lack time, then I will buy pre-cut or frozen FV at the grocery store).” (Vézina-Im et al. [58], p. 603)	Situation: barriers Response: solution to barriers Link: Template to link situation to responses
Excluded Study/Condition Examples	
“[…] You are more likely to carry out your intention to eat two extra portions of fruit or vegetables each day over the next three months if you make a decision about what you will eat and when and where you will do so. For example, you may find it useful to eat an apple instead of a chocolate bar for your afternoon snack, whilst watching Countdown in the living room. What I would like to do now is to decide with you what you will eat and when and where you will do this over the next 3 months. Let’s first record the times and places that you already eat a portion of fruit or vegetables, then we will make a plan for the 2 extra portions each day. […]” (Jackson et al. ([59] p2386))	Situation: “when and where”; times and places (example provides a perceivable cue) Response: extra portion of F/V *Ambiguous* Link: example (not in situation-response format); no link-formation instructions
“[…] This is my plan about fruit and vegetable consumption for the next seven days. During the next week, I plan to eat … (please, write down what type and amount of fruit/vegetable you plan to eat) at … (write down the time of the day) in/at … (describe the situation/place where you plan to eat your food). If I am tempted to eat something else, I plan to … (write down, how you plan to deal with temptations). […]” (Luszczynska & Haynes ([60] p1078))	Situation(1): Describe the situation/place (perceivable cues) *Ambiguous* Situation(2): Time of day (”calendar” planning; no perceivable cues). Response: What type of F/V (choice) Link: no link formation instructions (the reversed situation-response order is highly unusual for if-then planning). Comment: The second part introduces an if-then structure, but rather than specifying a clear situational cue, it appears to be meant as a contingency to what was planned before.
“[…] Please be specific about which meal or snack the substitution will be made each day. 1) What types of food, food additions or beverage can you do without or eat less of so that you eat more fruit and vegetables? Food item(s): When you usually eat it: […] How you will eat less of it: […] 2) When can you fit in at least 1 extra serving of vegetables […] each day? Please be specific about what time(s) of day, snack or meal you can substitute in an extra serving of vegetables. [Repeated once with focus on fruits]” (Djuric et al. ([61] p269))	Response: How to eat less of it (potentially appropriate goal-directed response) *Ambiguous* Situation: which meal or snack to substitute/ what time of day (mix of potential perceivable cue [old meal/snack] and “calendar” planning [time of day]) Link: Text boxes for different components; no link-formation instructions Comment: The instructions appear to induce a thorough deliberation of a person’s everyday routines and where to fit in extra portions of fruit and vegetables, but developing clear if-then plans to achieve these changes appear to be missing.

**Table 3:** Overview of central characteristics of the included comparisons

Code (condition label)	Int./Control (sample)	Intervention	Follow-up measure	Outcome (measurement summary)
Arm2015 [46] (“volitional help sheet implementation intention”)	33/23	Single-session plan formation	1 month	F (5 item, self-report, retrospective [1 month]) (quantity measure used)
Cha2009 [57] (“if-then implementation intention”)	104/97 (student)	Single-session plan formation	1 week	FV (1 item, self-report, open-ended, retrospective [1 week])
Cha2010a [62]	74/77 (student)	Single-session plan formation	3 months	FV (1 item, self-report, open-ended, retrospective [1 week])
Cha2010b [62]	72/61 (student)	Single-session plan formation (+ 3 month consumption questionnaire)	6 months	FV (1 item, self-report open-ended, retrospective [1 week])
Cha2012 [44]	266/127 (student)	Single-session plan formation	2 months	FV (2 item [F & V], self-report, open-ended, retrospective [1 week])
deB2017 [63] (“preparatory planning”)	57/62 (student)	Single-session plan formation	2 weeks	F (multi item, self-report, frequency and portions of different F categories, retrospective [2 weeks]
Gui2013a [47]	86/54	Four (20–30 min) face-to-face planning sessions (weekly) (control group: 4 mail-based, weekly delivered brochures on healthy eating)	12 months	FV (6 item, self-report, FV categories, retrospective [1 week])
Gui2013b [47]	85/70	Four (20–30 min) face-to-face planning sessions (weekly) (control group: 4 mail-based, weekly delivered brochures on healthy eating)	12 months	FV (6 item, self-report, FV categories, retrospective [1 week])
Sta2010 [45]	62/75	Single-session (2 h) plan formation + 1 week, 1, 2, and 4 month consumption questionnaire	24 months	FV (check-box based [FV items], self-report, 7-day diary)
Tap2014 [48]	50/50	4 month long, weekly if-then plan formulation and revision	4 months (of the 6 month total intervention time, no if-then planning information was given for the first 2 months)	FV (multiple fruit and vegetable items from Block et al., 1986), self-report
Vez2019 [58]	22/23 (mixed)	Single-session plan formation (+ 3 month consumption questionnaire)	6 months	FV (6 item, self-report, FV categories, retrospective [1 week])
Wie2011 [64]	334/40	Single-session plan formation	1 month	FV (2 item [F & V average/day], self-report, open-ended)

*FV* Fruit and vegetable consumption, *F* Fruit consumption, *Int*. Intervention

### Description of included studies

The included studies were peer-reviewed, randomized control trials published in health psychology-, behavioral- or nutrition journals. The included articles were published over an eleven-year period, between 2009 and 2019, with studies conducted mostly in western European countries (e.g., United Kingdom [5], Germany [2], France [1], Netherlands [1]), with the exception of one study being conducted in Canada [1]. The length between intervention and follow-up varied from 1 week to 24-months. The population included in the review were recruited from university students (4), with a mean age of ~ 21 years (range 18–44 years), and from adult populations (5; e.g., members of a health insurance company, employees of a German train company, and responding to a newspaper advertising), with an approximate mean age of ~ 42 years (range 19–65 years). One study had a mixed sample of university students and university employees (age range 18–44 years).

The gender distribution is strongly biased towards female participants, with 9 articles including 70% to 100% female participants, and only a single study that included 71% male participants. The mean percentage of female participants over all included studies is ~ 75%. In those studies reporting ethnicity, participants were predominantly Caucasian (~ 80%). In summary, the included population was predominantly from European countries, female, Caucasian, and young-to-middle aged adults (Table 3). The total population included in the meta-analysis is *N* = 2399 (1514 participants in the intervention conditions and 885 participants in the control conditions). The sample size imbalance between treatment conditions and control conditions is mostly a result of some reports having more than one relevant intervention condition that were combined to compare them to the respective single control group.

### Results of random-effects meta-analysis

**Fig. 2:**
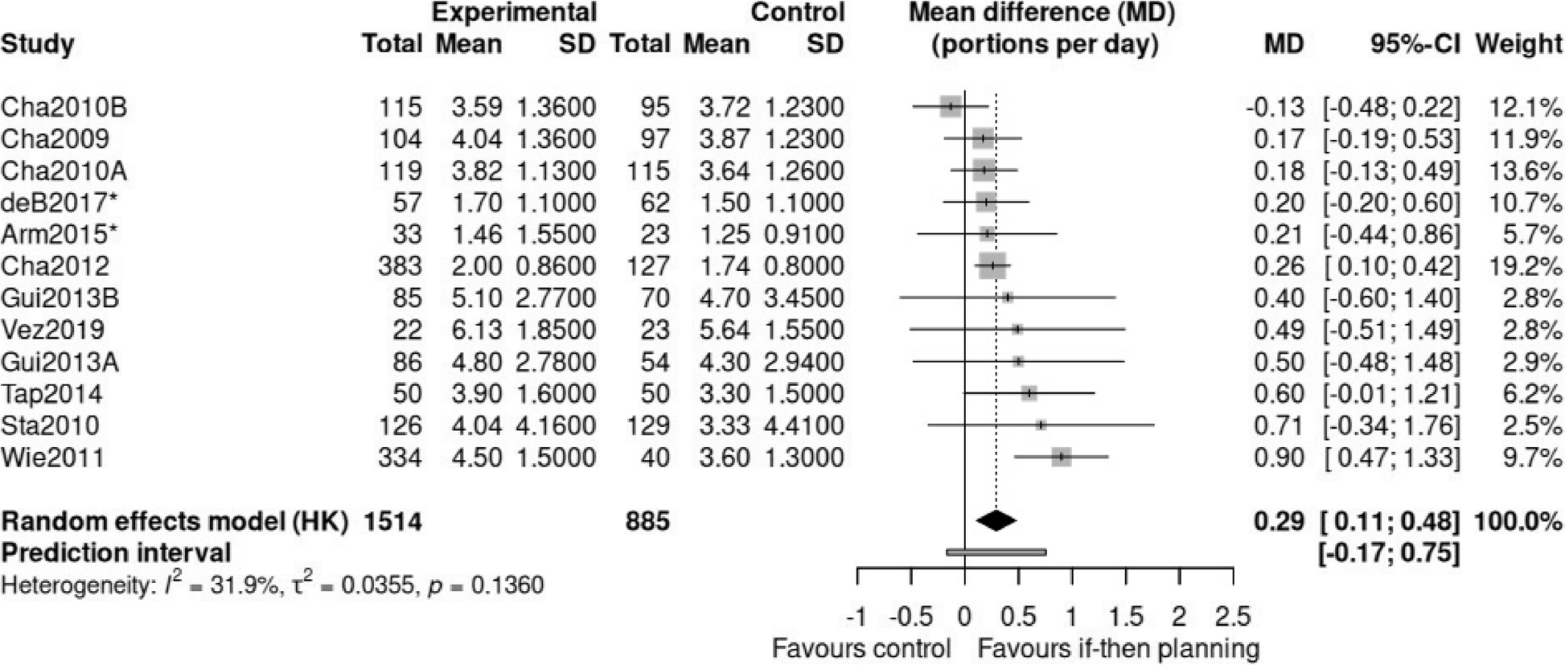
Forest plot of comparisons between if-then planning interventions and control conditions on fruit and vegetable consumption. Note. Forest plot illustrating the pooled mean difference (MD), using a random-effects model (REML), with HKSJ-adjustment. *The respective comparison contains only a fruit consumption measure

The mean difference (MD) from a total of 12 independent comparisons were pooled in an inverse-variance random-effects model, with restricted maximum likelihood (REML) estimator for between study heterogeneity, and Hartung-Knapp-Sidik-Jonkman (HKSJ) adjustment for confidence intervals around the pooled MD (Fig. 2; the data and analysis script is listed in Appendix 6). The pooled effect estimate indicates that if-then planning interventions can be favoured compared to the control groups (MD = 0.29; 95% CI: 0.11; 0.48), *t* = 3.55, *p* = .005. Overall, participants in the if-then planning conditions reported consuming a quarter of a portion more fruit and vegetables per day than the participants in the control conditions. The prediction interval indicates the possibility that a single future study can find both a null effect or up to a difference of 3/4 of a portion per day. A repetition of the analysis, set to estimate the standardized mean difference (SMD) resulted in an SMD of 0.20. According to Cohen’s effect size categorization [65], this is considered to be a small effect.

Visual inspection of the forest plot indicates that confidence intervals are generally overlapping and all comparisons show a descriptive effect in favour of the if-then planning condition. One exception is Cha2010B [62], which indicates a negative descriptive effect. Overall, the study’s results can be considered relatively homogenous, with a heterogeneity indicator *I*² = 32% (i.e., low to moderate inconsistency [55]) and the Tau² statistics for heterogeneity being non-significant (*p* = .14).

### Risk-of-bias results

**Fig. 3:**
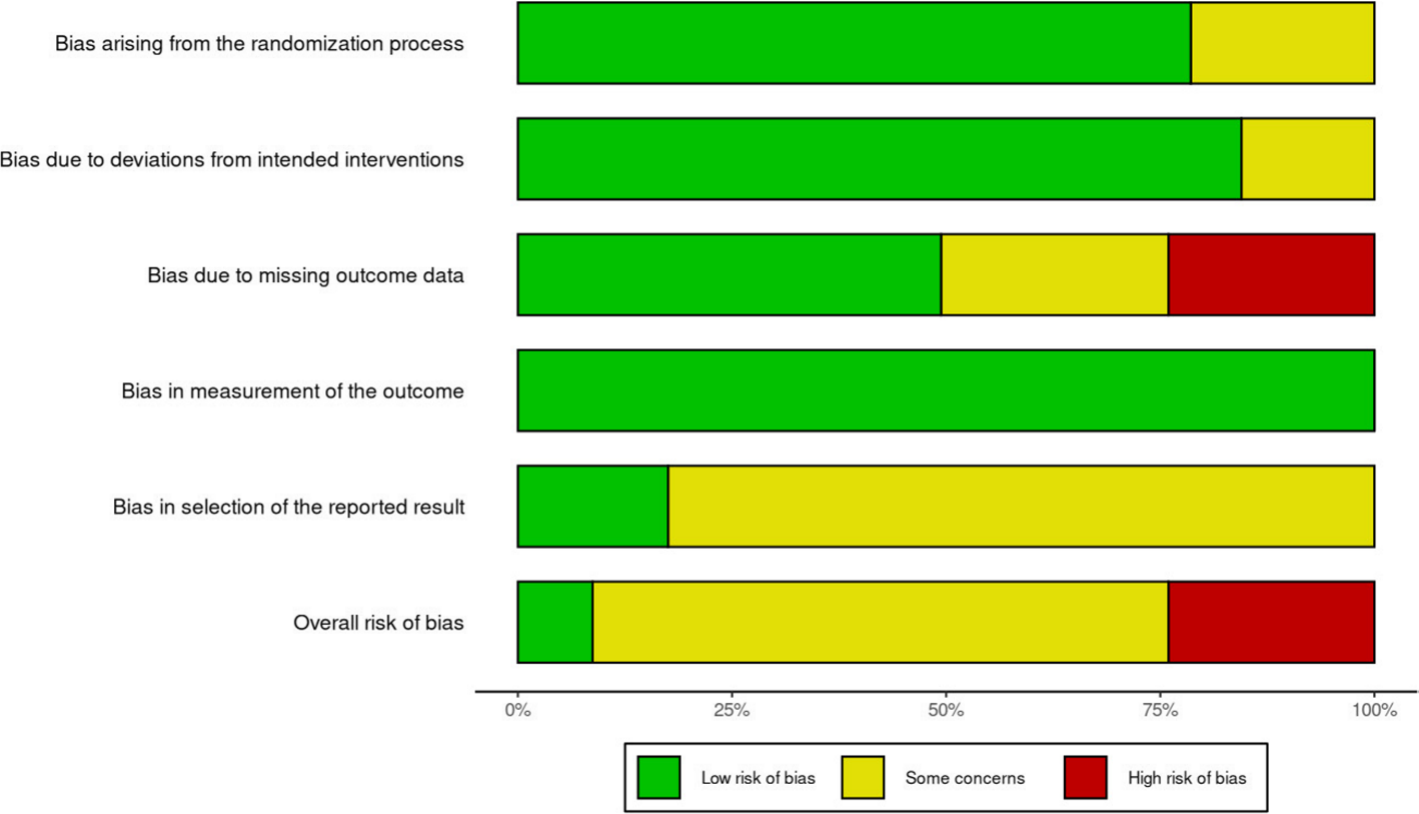
Risk-of-bias assessment. *Note*. RoB = Risk of bias

The complete risk-of-bias (RoB2) assessments are attached in Appendix 10. A summary illustration of the risk-of-bias assessment outcome of the five different domains and the overall risk assessment is illustrated in Fig. 3. The risk-of-bias analysis highlighted two areas of concern. First, two of the ten included articles did not apply an intention-to-treat procedure (i.e., they did not treat dropouts as “no-changers” in the analyses). This resulted in two “high” risk evaluations in the domain of “Bias due to missing outcome data”, which is also reflected in the “high” risk (red) area of the overall evaluation (see the Robustness and Publication Bias section for the results of the meta-analysis with these two high-risk articles removed; in short, this removal has no consequences for the meta-analysis’ conclusion).

The second aspect of concern is that the majority of the articles did not include information whether the reported analyses had been decided before the availability of the data (e.g., pre-registration). Thus, there was no information in the articles whether intended and reported analyses deviated. This concern is reflected in the large “some concerns” (yellow) area in the “Bias in selection of the reported results” domain, which directly contributed to the large “some concerns” (yellow) area in the summary bias at the bottom of Fig. 3. We will discuss the relevance of this aspect in the Discussion section. In short, in line with the RoB guidelines, we labeled these aspects as “some concern” but we do not believe that they are a limitation of the present research as our meta-analysis is not based on the analyses that the authors of the respective articles decided to report.

### Robustness and publication bias

Because of the overall homogeneity of the study results, the outcome of the meta-analysis is unlikely to change depending on including or excluding individual studies. However, we repeated the meta-analysis, with the two articles excluded that we identified as “high risk” in the risk-of-bias analysis (i.e., the two articles not implementing an intention-to-treat method). The heterogeneity of the effects from this analysis based on *N* = 10 comparisons increased (I² = 43%, moderate inconsistency [55]; Tau² statistics for heterogeneity being marginally significant, *p* = .073). However, the central result remained the same. The alternative pooled effect estimate was *MD* = 0.34 [*CI*: 0.11; 0.57] in favour of the if-then planning conditions (*t* = 3.33, *p* = .009).

**Fig. 4:**
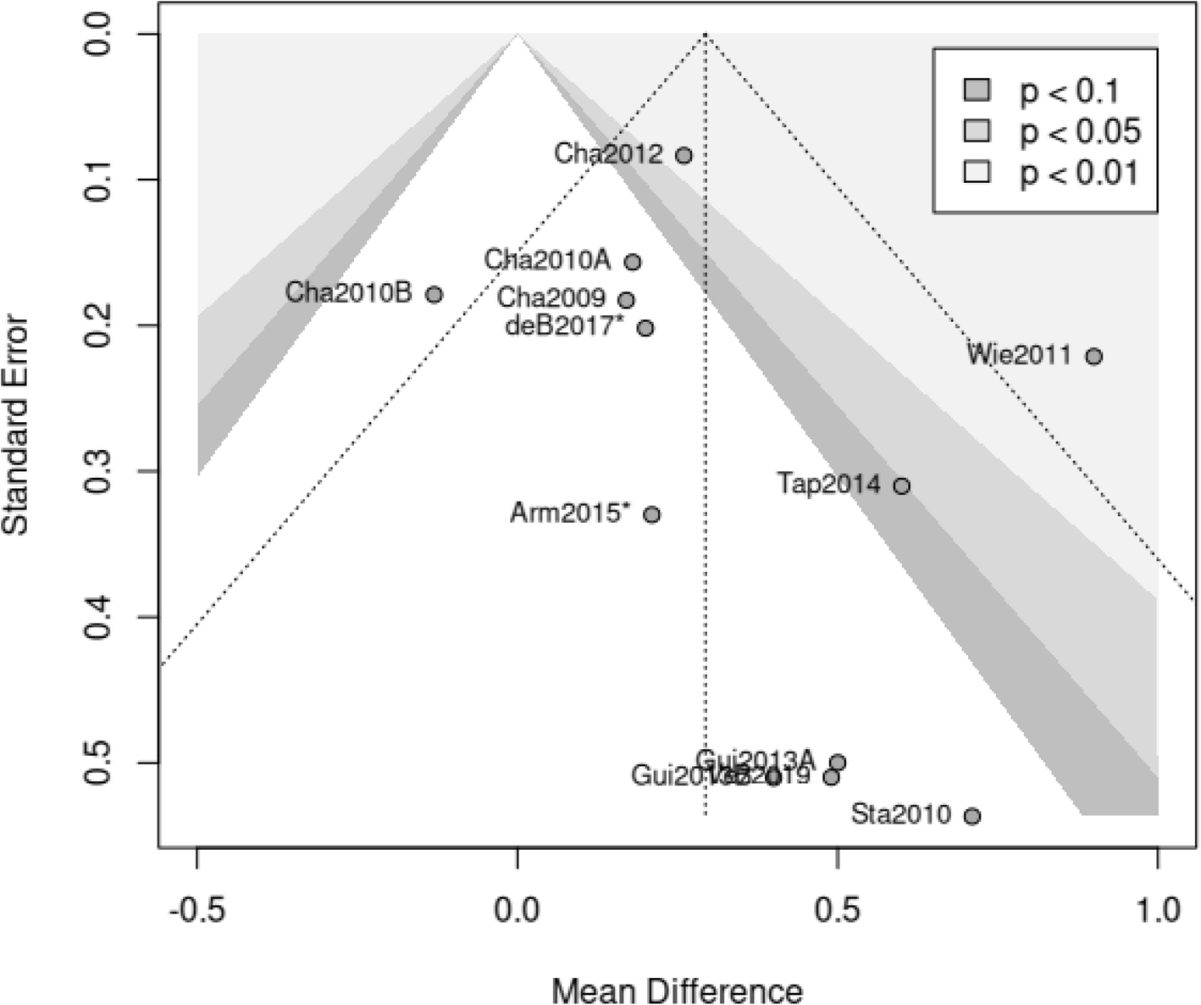
Contour-enhanced Funnel Plot. *Note*. The references for the depicted study codes are presented in Table 3

A contour-enhanced funnel plot is depicted in Fig. 4 to visually depict the mean differences under varying significance levels for the main analysis to check for evidence of a publication bias [66]. Statistically, a linear regression test of funnel-plot asymmetry (i.e., indication of publication bias [67]) showed no considerable asymmetry (*t*(10) = 1.05, *p* = .320). Overall, it is reassuring that the studies showing comparatively large effects are rather high-powered studies (upper part of the graph, low standard error). In sum, the study results are relatively homogenous and we have no strong indication to suspect publication bias.

## Discussion

In this systematic review and meta-analysis, we assessed the effectiveness of the self-regulatory strategy if-then planning [19, 20] to increase fruit and vegetable intake in adults. We identified ten research reports and twelve independent comparisons that satisfied the requirements for our analysis by reporting fruit-and/or-vegetable consumption, implementing an if-then planning intervention in a strict sense, and including an appropriate active control group. We found a small, statistically significant, positive effect for the if-then planning conditions compared to the control conditions. On average, participants reported consuming one quarter of a portion per day more fruit and vegetables (MD = 0.29; SMD = 0.2) in measures assessed between one week and 24 months after completing the if-then planning intervention.

The unique contribution of the present meta-analysis lies in its focus on two specific aspects: increasing fruit and vegetable consumption as an outcome and estimating the effectiveness of if-then planning as an isolated component. The focus on increasing fruit and vegetable consumption is warranted, as it represents a modifiable, proactive behavior with significant health benefits [3, 4]. The focus on if-then planning as a specific intervention component provides an evaluation of its effectiveness, helping health-care providers determine how to use it—for example, as one component in a larger intervention or as the sole intervention component in contexts where resource-intensive interventions are not practical.

This specific focus is not present in previous meta-analyses. Previous meta-analyses on if-then planning [35–37] have included studies that combined if-then planning with other intervention components (e.g., self-efficacy, motivational cues) and included studies labeled by the original authors as if-then planning, even when the intervention did not include the central characteristics of if-then planning. Consequently, the observed effectiveness in those studies cannot be clearly attributed to the if-then planning component; it could be a consequence of if-then planning alone, additional intervention components alone, more conventional planning, or a combination of all of them. In the present meta-analysis, we included only direct comparisons of if-then planning versus control groups (or studies with a parallel inclusion of additional aspects in both the intervention and control groups) and only studies implementing if-then planning in a strict sense. This approach ensures that the observed effectiveness is more clearly attributable to the if-then planning component alone.

### Effect size comparisons with prior studies

The present meta-analysis indicates a small, statistically significant effect with an SMD of 0.20. Direct comparisons to previous studies are complicated by methodological differences, as summarized in the previous section. Earlier studies on increasing healthy food consumption report effect sizes of 0.51 [35], 0.33 [36], and 0.26 (or 0.12 when adjusted for publication bias [37]). Our present outcome (0.20) is at the lower end of these prior estimates. There is little basis for speculating whether the comparatively low estimate is due to the strict focus on if-then planning, the focus on fruit and vegetable consumption, or other methodological factors (e.g., control-group selection). However, we can conclude that our meta-analytic summary of previously published applied interventions using if-then planning in a strict sense to increase fruit and vegetable consumption demonstrates a small positive effect in favor of the intervention. Our current estimate, based on twelve comparisons, is slightly more robust compared to a prior attempt to isolate if-then planning in a strict sense, which yielded an effect size of 0.32 based on just three studies [30]. Finally, compared to another prior estimate (0.43) [38], which is biased toward including laboratory studies and excluding applied studies, our current estimate is more applicable to applied intervention scenarios.

### Implications for health-care practitioners

The practical contribution of our present analysis is greater clarity on the potential effect of if-then planning as an isolated component. The inclusiveness of previous meta-analyses has advantages, such as providing generalized effect estimate that reflects the overall trend across variations in individual intervention procedures. However, this inclusiveness poses challenges for practitioners who must implement a single intervention. The inclusive prior analyses offer limited guidance for determining which aspects are most relevant for the intervention to work. In contrast, while our present meta-analysis does not have the broad scope of prior analyses, it provides a more specific informational basis on the component under investigation. On this basis, practitioners can evaluate whether the costs and effort required to include if-then planning as a component in their intervention would be beneficial and justified, considering the expected average effect of increasing fruit and vegetable consumption by a quarter of a portion per day.

The essence of the planning-related information provided to participants in the analyzed studies was relatively homogeneous and could be delivered as written instructions that fit one or two standard A4 pages. This information included (1) highlighting the importance of eating enough fruit and vegetables, (2) thinking about what behaviors are required (e.g., buying, preparing, packing) to meet the goal, (3) thinking about situations in which these behaviors could be performed (e.g., local grocery shop, before leaving for work), and (C) providing space to formulate a set of if-then plans that explicitly link the previously identified situational cues to the respective intended responses. Such help sheets could be integrated in nutrition-counseling sessions, or provided to potential users to complete on their own. Hence, the investigated if-then planning procedure is a flexible tool, well suited for clinical practice, in which time-management and cost are important considerations.

### Limitations of the identified studies

There are two central limitations to the identified studies with regard to the validity of our effectiveness assessment: First, the sample investigated in the included studies was predominantly from western European countries, female, Caucasian, and young-to-middle aged adults. Thus, the effectiveness evaluation primarily applies to this population group. While we do not see particular reasons why a strategy that is based on a fundamental stimulus-response learning should not apply to other samples with other characteristics, additional research with more diverse samples is required to test this empirically.

Second, a more critical limitation of the included studies is the exclusive use of retrospective self-report measures to quantify fruit and vegetable consumption. Furthermore, most of these measures were based on one or two questions to estimate the amount of portions per day. A central problem in self-reports is that they can be systematically biased, and in the context of testing an intervention, the danger is to create demand characteristics in the intervention condition to report high fruit and vegetable consumption. While this possibility cannot be excluded, the choice of control conditions strengthens our confidence in the present estimates. We only included studies with control conditions that provided information that was similar to the intervention condition with respect to providing information on the health benefits of increasing fruit and vegetable consumption and asking participants to increase their consumption. Thus, the control-condition participants are likely to have experienced the same demands as the participants in the intervention condition.

Nonetheless, retrospective self-reports can easily be biased and evaluations of interventions for an important, health-beneficial behavior as fruit and vegetable consumption should not be based solely on self-reports. It is resource efficient to use easy to implement methods for initial evaluations of the effectiveness of an intervention. However, after initial (positive) evidence is attained, it is desirable to go beyond the typically implemented self-report methods.

### Limitations of the present meta-analysis

Arguably, the central strength of the present meta-analysis is also its central weakness. The theoretical focus on a specific underlying mechanism, required selecting articles based on descriptions of how participants were instructed in the respective study. Inclusion and exclusion decisions could have been unintentionally influenced by knowledge of a study’s results. Inclusion decisions have been made based on the information presented in Table 2 (the full information is presented in Appendix 4); when it was possible to identify instructions that related to a perceivable situation, a goal-directed response, and explicitly linking both components. With the inclusion of the table, we aim to make this decision process as transparent as possible.

We believe that the additional degree of uncertainty that we cannot avoid is justified by the ends of making a novel contribution. Completely removing the uncertainty from the selection process would have resulted in including all studies that vaguely resemble if-then planning (or are labeled by the original authors as such). As argued before, this effectiveness outcome would have been based on a broad range of potential mechanisms and this has been done in previous analyses related to different outcomes [35–37]. This was not the goal of the present analysis. Instead, we accepted this limitation for the benefit of narrowing down the mechanism that contributed to the investigated interventions.

With regards to the systematic risk-of-bias analysis, we identified two critical aspects in the risk-of-bias analysis. First, two articles were evaluated as high risk in the overall assessment mainly because of not implementing an intention-to-treat method (including follow-up dropouts as “no-changers”). To test whether the meta-analysis result is affected meaningfully by these articles, we ran the analysis again with the respective articles removed. The pooled mean difference changed from 0.29 portions per day in the analysis with all articles to 0.34 in the analysis with the high risk comparisons excluded. Thus, we conclude that the high-risk articles are not meaningfully affecting the central outcome of the reported main analysis.

Second, for the majority of the articles, it was not possible to know whether there were differences in the intended analyses (before data was available) and reported analyses. This results in intransparency of analysis decisions with the potential repeated and adjusted analyses based on observed intermediate results. However, we do not think that our meta-analysis’ results are negatively affected by this problem. We did not rely on analyses that the respective authors reported in the manuscripts. Instead, we collected and conducted the meta-analysis on the descriptive statistics of the intervention conditions and the control conditions reported in the articles, independent of the analysis choices done in the original articles.

A serious concern is that we did not identify any unpublished studies. There is a concern of publication bias (i.e., overestimating effect sizes because ‘unsuccessful’ studies are not published) due to the ‘file drawer’ problem in implementation intention research. The ‘file drawer’ problem has many causes, not all of which contribute to publication bias (e.g., researchers losing interest after a null result contributes to publication bias vs. discovering a study design flaw does not contribute to publication bias) [68]. Our analysis included only relatively resource-intensive intervention studies, half of which involved non-student populations and all tracked samples over time. One might argue that such studies are less likely to end up as “file drawer” studies compared to less resource-intensive single-session, student sample, laboratory studies due to higher prior investment.

Supporting this reasoning, most intervention-control comparisons identified in our meta-analysis are not statistically significant. Nonetheless, these studies were published and did not end up as ‘file drawer’ studies because of the non-significant main effect. In summary, we interpret the lack of responses to our request for unpublished data^1^, the absence of preregistrations without corresponding published studies, and the fact that most individual study outcomes reported are not statistically significant as evidence that there may not be many unpublished ‘file drawer’ studies on the specific topic of our meta-analysis.

No protocol was submitted for the present meta-analysis; however, an unpublished protocol is available [41]. A notable deviation from the protocol is the shift from the broad “nutrition-related measures” as outcome to the more narrow “fruit-and-vegetables consumption”. Details for the fruit-and-vegetable outcome are present in the protocol as a subgroup analysis, and the decision was enacted before any analyses outcomes were known to the authors. Second, the investigation of two moderator variables (student vs. non-student population, researcher- vs. participant-formulated plans) were not considered in the present report as the statistics performed with the small number of studies prevent any meaningful conclusion on these aspects.

## Conclusion

Fruit and vegetable consumption is a modifiable behavior with significant health benefits [3, 4], and underconsumption is an issue for Europe’s [7–9] and the majority of the world’s population. Health care providers need evidence-based and resource-efficient strategies to guide and support patients to increase their fruit and vegetable consumption. We tested such a strategy, namely, if-then planning (in a strict sense). We identified ten articles and twelve relevant comparisons reports with follow-up measures done between 1 week and 24 months after the intervention and including *N* = 2399 participants. We found that self-reported fruit and vegetable consumption was a quarter of a portion per day higher in the intervention group compared to similarly motivated participants not receiving the if-then planning intervention. While the effect size of the investigated intervention is small, its low resource requirements make it an accessible option for promoting fruit and vegetable consumption either as one component in a larger intervention or as a single component when more intensive interventions are not feasible. This conclusion is based on the assumption that any increase in fruit and vegetable consumption is beneficial. However, whether an increase of one-quarter of a portion per day is clinically relevant in reducing the risk of non-communicable diseases must be evaluated from a medical and nutritional perspective.

## Supplementary Information

Supplementary Material 1.

Supplementary Material 2.

## Data Availability

All relevant data and analyses are available as appendix (e.g., R-scripts).

## Footnotes

1 We received responses to our e-mail (e.g., interest in our analysis outcome), so that we can be sure about its successful distribution, but we did not receive information that pointed towards unpublished studies.

## Abbreviations

Chi^2^: χ^2^ – statistics/ Q-statistics
HKSJ: Hartung-Knapp-Sidik-Jonkman adjustment
I^2^: I^2^ statistics – the inconsistency between studies
PRISMA: Preferred Reporting of Items for Systematic Reviews and Meta-analyses
REML: Restricted maximum likelihood estimator
RoB 2: Revised Cochrane risk-of-bias tool for randomized trials
[anonymized]: [anonymized]
Tau^2^: τ^2^ - statistics/ heterogeneity
MD: Mean difference (unstandardized)
SMD: Standardized mean difference
