# Supplementary material for: A systematic review and meta-analysis on the effectiveness of if-then plans – in a strict sense – to facilitate fruit and vegetable consumption in adults
Source: Int J Behav Nutr Phys Act. 2026 Apr 15;23:51. doi: 10.1186/s12966-026-01915-y (PMC13195971; doi:10.1186/s12966-026-01915-y)
Supplement: Supplementary file 1 — Supplementary Material 1. [file 12966_2026_1915_MOESM1_ESM.docx]

Appendix
Appendix 1. Search Strategy in MEDLINE
Sets 1-2 & 21 are the text words (words found in the title, abstract or keywords of a record) for the intervention. They are combined using OR.	1. implementation intention*.ab,kw,ti. 2. if then plan*.ab,kw,ti.  21. #1 or #2	Intervention (I)
Sets 3-20 & 22 are the MeSH & text words (words found in the title, abstract or keywords of a record). They are combined using OR.	3. exp "diet, food, and nutrition"/ 4. diet.ab,kw,ti.  5. food.ab,kw,ti. 6. nutrition.ab,kw,ti. 7. exp fruit/  8. exp vegetables/  9. snack*.ab,kw,ti.  10. sugar.ab,kw,ti.  11. fruit*.ab,kw,ti.  12. vegetable*.ab,kw,ti. 13. salt*.ab,kw,ti.  14. exp Sodium Compounds/  15. exp dietary fats/  16. fat*.ab,kw,ti.  17. exp Snacks/ 18. exp feeding behavior/  19. eat* behavior*.ab,kw,ti. 20. eat* behaviour*.ab,kw,ti.  22. #3 or #4 or #5 or #6 or #7 or #8 or #9 or #10 or #11 or #12 or #13 or #14 or #15 or #16 or #17 or #18 or #19 or #20	Outcome (O)
Set 23 is the combination of intervention and outcome. Population and study design will be screened manually for eligibility.	23. #21 and #22	Combination of I & O


Appendix 2.
Approach and outcome of original-instruction inquiries.
To select studies that implemented if-then planning in a strict sense, we contacted the leading authors of all articles identified as relevant based on the remaining inclusion criteria (20 articles). We used the email addresses provided in the respective articles as well as the most current addresses found online for each leading author. We asked the leading authors to share the original, complete instructions given to participants for the planning procedure. Out of the 17 authors contacted, we received 10 responses. Five authors provided the original instructions, and one additional author confirmed our categorization of their article as not involving if-then planning in a strict sense. Three authors acknowledged that the original instructions were no longer available. One author initially responded but did not follow up regarding the availability of the instructions. Six authors did not respond to our email, and one author's email addresses returned an error message indicating that the addresses were invalid. In summary, for most articles, we relied on the information available in the published articles. In five cases, we confirmed our categorization based on the information provided by the original authors. In one case, we revised our initial categorization. An excluded article, due to the lack of any planning information in the article, was included based on details provided in the original instructions.


Appendix 3. Description and R script illustrating the performed adjustments to the reported statistics.
Chapman and Armitage (2012) reported two intervention conditions but only one control group, and fruit and vegetable consumption was reported as separate measures. To take full advantage of the available data, we combined the fruit and vegetable measures and the two intervention conditions. The R-script and the data for these calculations is listed below. In essence, we first combined the separate fruit and vegetable scores for each group (control, “combined-II”, and “separate-II”). The formula requires a correlation coefficient. As the exact correlation coefficient was not available, we used an estimated fruit-and-vegetable correlation of .32 (based on Michels et al., 2005). In the next step, we used the combined fruit-and-vegetable score (from step 1) and combined the two intervention groups (“combined-II” and “separate-II”) based on a procedure provided by the Cochrane handbook (2011) “Formulae for combining groups”. Stadler et al. (2010) reported per-week scores, which were divided by 7 to result in a fruit-and-vegetable score per-day. In addition, only baseline standard deviation was reported. However, the result plot indicated similar variance estimates for all measurement time points. Thus, we used the baseline standard deviation value also for the follow-up measures. As the standard deviation was reported for the per-week mean value, we transformed the per-week standard deviation to a per-day standard deviation estimate. The used formula SD.day=SD.week/sqrt(7) is likely to lead to an inflated variance estimate. Thus, the statistics from this report are likely to slightly underestimate the true effect strength. In three comparisons (from two articles; Armitage et al., 2015 and Guillaumie et al., 2013), we observed irregularities in the reported standard deviation that made us suspect that the reported values are mislabelled standard errors. We transformed the values that we suspected to be standard errors into standard deviations, SD=SE*sqrt(N), and the meta-analysis outcome is based on these adjusted values. A forest plot of the meta-analysis outcome with the original values is presented in Appendix 3. An R-script for all described transformations can be found in Appendix 4. Finally, we made no adjustment to the ‘cup’ measure in Tapper et al. (2014). We treated 1 cup in Tapper et al. as equal to 1 portion in the other studies. This decision is based on the assumption that participants' retrospective consumption judgements are unlikely to reflect accurate measures in gram but rather remembered consumption events that are not so much affected by whether they were labelled 'portion' or 'cup' with slightly different definitions of how many grams each reflects.
# ------------------------------------------------------------------------------ # 1. Chapman and Armitage 2012 group combination # ------------------------------------------------------------------------------  # General SD-combination formula for dependent samples (multiple measures) # RES = sqrt( 1/4*SD1^2 + 1/4*SD2^2 + 1/2*CORR*SD1*SD2 )  # # 1.1) Combining fruit and vegetable scores from same group. # ------------------------------------------------------------------------------  # # Control group #  ## Mean calculation N   = 197                # N control M1  = 1.72               # M fruit control M2  = 1.75               # M vegetables control Res = ( N * M1 + N * M2 ) / ( N + N ) print(Res)               # M = 1.735  ## Standard deviation (SD) calculation SD1  = 0.98              # SD fruit control SD2  = 1.00              # SD vegetables control CORR = 0.32              # correlation estimate taken from                          # https://academic.oup.com/aje/article/161/10/987/104766 Res = sqrt( 1/4*SD1^2 + 1/4*SD2^2 + 1/2*CORR*SD1*SD2 ) print(Res)               # SD = 0.8043   # # "Combined II" group #  ## Mean calculation N   = 183                # N "combined II" group M1  = 2.14               # M fruit "combined II" group M2  = 1.9                # M vegetables "combined II" group Res = ( N * M1 + N * M2 ) / ( N + N ) print(Res)               # M = 2.02  ## Standard deviation (SD) calculation SD1  = 1.03              # SD fruit "combined II" group SD2  = 1.06              # SD vegetables "combined II" group CORR = 0.32              # correlation estimate taken from                          # https://academic.oup.com/aje/article/161/10/987/104766 Res = sqrt( 1/4*SD1^2 + 1/4*SD2^2 + 1/2*CORR*SD1*SD2 ) print(Res)               # SD = 0.849   # # "Separate II" group #  ## Mean calculation N   = 200                # N "separate II" group M1  = 1.94               # M fruit "separate II" group M2  = 2.01               # M vegetables "separate II" group Res = ( N * M1 + N * M2 ) / ( N + N ) print(Res)               # M = 1.975  ## Standard deviation (SD) calculation SD1  = 1.07              # SD fruit "separate II" group SD2  = 1.09              # SD vegetables "separate II" group CORR = 0.32              # correlation estimate taken from                          # https://academic.oup.com/aje/article/161/10/987/104766 Res = sqrt( 1/4*SD1^2 + 1/4*SD2^2 + 1/2*CORR*SD1*SD2 ) print(Res)               # SD = 0.8774     # # 1.2) Combining the previously combined FV-scores for both groups #      ("combined II" and "separate II" group) # --------------------------------------------------------------------------------------  ## formula from https://www.statstodo.com/CombineMeansSDs.php ##  myDat = (" n    mean   sd 183  2.02   0.849 200  1.975  0.8774  ") myDataFrame <- read.table(textConnection(myDat),header=TRUE)  # conversion to data frame     myDataFrame                                                   # optional check input  nr = nrow(myDataFrame)   # number of rows ex <- rep(0,nr)          # array to contain SUMx exx <- rep(0,nr)         # array to contain SUMx2 tn = 0                   # total n tx = 0                   # total SUMx  txx = 0                  # total SUMx2 for(i in 1:nr) {   ex[i] = myDataFrame$n[i] * myDataFrame$mean[i]   exx[i] = myDataFrame$sd[i]^2 * (myDataFrame$n[i]-1) + ex[i]^2 / myDataFrame$n[i]   tn = tn + myDataFrame$n[i]   tx = tx + ex[i]   txx = txx + exx[i]   } # concatenate SUMx and SUMx2 to data frame myDataFrame$ex <- ex myDataFrame$exx <- exx myDataFrame             # show data frame # Calculate combined values tMean = tx / tn tSD = sqrt((txx-tx^2/tn)/(tn-1)) print("Combined n, mean, and SD"); print(c(tn,tMean,tSD))  # Result Combined (F/V & comb/sep) Intervention Group # N     M        SD # 383   1.9965   0.8631  # Result Combined (F/V) Control Group # N     M        SD # 127   1.735    0.8043    # ------------------------------------------------------------------------------ # 2. SE to SD calculations # ------------------------------------------------------------------------------  # Armitage et al., (2005) ## intervention 0.27 * sqrt(33) ## control 0.19 * sqrt(23)  # Guillaumie et al. (2013), Comp A ## intervention 0.30 * sqrt(86) ## control 0.40 * sqrt(54)  # Guillaumie et al. (2013), Comp B ## intervention 0.30 * sqrt(85) ## control 0.40 * sqrt(70)   # ------------------------------------------------------------------------------ # 3. Per-week to per-day standard deviation (Stadler et al., 2010) # ------------------------------------------------------------------------------  ## intervention 11.01 / sqrt(7) ## control 11.68 / sqrt(7)


Appendix 4. Instructions and Comments on the Inclusion Versus Exclusion Reasoning.
Intervention instructions	Comments
Included Studies
“[...] containing lists of ten critical [temptation] situations and ten appropriate responses. [...] temptation items were translated into "if" statements [...], the processes of change items were translated into "then" statements [...]. Participants in the volitional help sheet condition [...] were then asked to draw links between as many critical situations and appropriate responses as they wanted and thereby form implementation intentions […] (Armitage, 2015, “volitional help sheet implementation intention” condition, p. 602)	Situation: temptation situations (perceivable cues) Response: responses to temptation (behavioral responses) Link: link formation by connecting situation and response
“[...] paying particular attention to the situations in which you will implement these plans. You could choose to focus on planning what fruit and vegetables to buy, how you will prepare them [...] you may find it useful to state, "If it is lunchtime at university, then I will eat an apple instead of crisps!" Please write your plans in the space provided, following the format in the example ("if ... then ..."). [...] (Chapman et al., 2009, “if-then implementation intention” condition, p. 321)	Situation: Crisps at lunch (example, perceivable cue) Response: what F/V to buy, how to prepare (behavioral responses) Link: final plan written in if-then format
“[…] planning is more effective if you first identify a situation, then decide what you will do in that situation. For example, […] ‘If it is lunchtime at university, then I will eat an apple instead of crisps!’ Or you could choose to focus on planning where to by fruit and vegetables or when and how you will prepare them. Please write your plans […] following the format in the example (‘if...then…’). [...] (Chapman and Armitage, 2010, p. 370)	Situation: example lunchtime; where to buy (perceivable cue) Response: example, eat an apple; how to prepare (behavioral response) Link: instructions to follow the if-then format
“[…] planning is more effective if you first identify a situation, then decide what you will do in that situation. For example, [...] ‘‘If it is lunchtime at university, then I will eat a salad instead of chips!’’ Alternatively, [...] focus on planning where to buy fruit or vegetables, or when and how you will prepare them. [...] Please write your plans in the space provided, following the format in the example (‘‘if. . .then. . .’’) [...] (Chapman and Armitage, 2012, p. 30)	(same instructions as the included Chapman and Armitage, 2010 study)
“[…] young adults are more likely to […] increase their fruit intake [...] when they formulate specific preparatory actions […]. Three specific preparatory actions were provided, namely (i) buying fruits to have them available [...], (ii) taking fruits with them [...], and (iii) placing fruits at a visible location [...].” [followed by three examples] “when I am at the Central station after a day at university, I will go to the supermarket at the central station and buy a banana to have it available […]” (de Bruijn et al., 2017, “preparatory planning” condition, p. 164)	Situation: example plan includes location (perceivable cue) Response: example preparatory actions provided (behavioral responses) Link: example plan in situation-response format  Comment: see below for the instructions of the excluded “action planning” condition from the same article.
“[…] Participants were required to identify the barriers to achieve the POs [performance objectives] and then to plan, in detail, how the behaviour or preparatory behaviours would be performed and how the barriers […] would be overcome. They formulated and wrote down between two and five II plans […]. The plan were of two types ‘To do x, I will do y (where, when and how); and ‘If the situation x arises, then I do y’ “ (Guillaumie et al., 2013, p. 84)	Situation: barriers Response: response to overcome barriers Link: One of the example templates is in an if (situation)-then (response) format
“[...] They wrote down [...] the most critical obstacle (e.g., “no fruits at work”) together with events and experiences they associated with this obstacle; and (d) formed three implementation intentions with the following questions: (1) “When and where does the obstacle occur, and what can I do to overcome or circumvent the obstacle?”; (2) “When and where is an opportunity to prevent the obstacle from occurring, and what can I do to prevent it from occurring?”; and (3) “When and where is a good opportunity for me to act in a goal-directed way, and what would the goal-directed action be?” For example, [...] To prevent the obstacle from occurring the participant could use the implementation intention “If I pass the greengrocer on my way to work then I buy apples!” (three examples in and if(situation)-then(action) format given)” (Stadler et al., 2010, p. 277-278)	Situation: obstacles, situation in which to overcome obstacles (events/experiences = perceivable cues) Response: response to overcome obstacles Link: example plans in if (situation)-then (response) format
“What situation might make it hard for you to achieve your goal? What obstacles stand in your way? Write these down in the space below. [...] We’d now like you to think about exactly WHEN, WHERE and HOW you will change your behaviour and write it in the space below in the form of an ‘if-then’ statement. You may also find it helpful to incorporate the obstacles you identified on the previous page. [...] Here are a few examples to show you what we mean: [...] ‘If I am in the pub and someone offers me crisps, then I will resist the temptation to have some and say no.’ Write your plan here: If ... then ...” (Tapper et al., 2014, Appendix 3)	Situation: event/perceivable cue examples AND obstacles Response: goal-compatible behaviour OR solution to obstacle Link: If-then template (details provided in personal communication, 06. February 2025 from the studies first author)
“[...] a table containing a list of potential barriers to FV consumption on one side and a list of potential solutions on the other side. [...] The questionnaire contained 12 barriers and one space to write down a barrier not already mentioned in the table. Each barrier was preceded by ‘if’. [...] The questionnaire also contained 12 solutions and one space to write down a new solution to overcome barriers to FV consumption. Each solution was preceded by ‘then’. [...] Participants were instructed to link each barrier that personally applied to them to a solution to overcome it (e.g. If I lack time, then I will buy pre-cut or frozen FV at the grocery store).” (Vézina-Im et al., 2019, p. 603)	Situation: barriers Response: solution to barriers Link: Template to link situation to responses
“[…] Subsequently, participants were asked to specify coping plans, i.e. up to five potential risk situations and corresponding coping responses: [...] write down up to three barriers that might prevent you from consuming five servings of fruit and vegetable’. Three fields were provided for the participant’s personal barriers. […] Subsequently, the specified personal barriers were presented one after the other, and participants were asked ’What could you do to consume five portions of fruit and vegetable nevertheless?’ and given the possibility to generate one strategy per barrier.” (Wiedemann et al., 2011, p. 45)	Situation: risk situations/barriers Response: coping response Link: template to link barrier and coping response  Comment: The intervention included planning instructions to generally eat more F/V. These general instructions alone would not have been categorized as if-then planning in a strict sense. Another study by the authors (Wiedemann et al., 2012) only included the general instructions, without the barrier-coping plans, and was excluded for that reason.
Excluded Studies
“[…] This is my plan about fruit and vegetable consumption for the next seven days. During the next week, I plan to eat ... (please, write down what type and amount of fruit/vegetable you plan to eat) at ... (write down the time of the day) in/at ... (describe the situation/place where you plan to eat your food). If I am tempted to eat something else, I plan to … (write down, how you plan to deal with temptations). [...]” (Luszczynska & Haynes, 2009, p. 1078)	Situation(1): Describe the situation/place (perceivable cues)  Ambiguous Situation(2): Time of day (”calendar” planning; no perceivable cues). Response: What type of F/V (choice) Link: no link formation instructions (the reversed situation-response order is highly unusual for if-then planning).  Comment: The second part introduces an if-then structure, but rather than specifying a clear situational cue, it appears to be meant as a contingency to what was planned before.
“[…] asked to generate [...] action plans in illustrations of notepads in accordance to their allocated condition. Each notepad consisted of four interrogative (‘when’, ‘where’, ‘what kind’, and ‘how’), and blank space to allow for participants’ individual plans. Plans were formed regarding specific opportunities in which to consume one serving, and all plans should relate to one average day to ensure that a plan does not include multiple servings. A sample action plan was provided to facilitate comprehension. Participants were asked to check the completeness of their plans and to memorize them. [...]” (Wiedemann et al., 2012, p. 388-389)	Response(1): “how” (goal-directed responses)  Ambiguous Situation: specific opportunities; however, to be entered following “when” and “where” prompts (”calendar” planning; no perceivable cues) Response(2): “what kind” (choice) Link: example plan; no instructions to turn the prompted (“when”, “where”, “what kind”) responses into an explicit situation-response plan.  Comment: This study did not include the specific if(barrier)-then(coping response) component that made us include the same author’s 2011 study).
“[…] Please write in below when, where and how you will introduce more fruit and vegetables into your diet over the next week. You are free to choose how you will do this but please formulate your plans in as much detail as possible. Please pay particular attention to the situations in which you will implement these plans. For example, you might plan how you could replace five other snacks or foods you normally eat with fruit and vegetables. Or you could see how you can incorporate them into meals that you would normally eat anyway. […]” (Troop, 2013, p. 863)	Response: “how” (goal-directed response)  Ambiguous Situation: “when, where” (unclear whether this would lead to specifying perceivable cues rather than calendar-like day/time planning) Link: no link-formation instructions  Comment: Response alone is referred to as “plan”; similar text components as the excluded Chapman et al., 2009, “global implementation intention” and Armitage et al., 2015, “self-generated implementation intention”)
“[...] You are more likely to carry out your intention to eat two extra portions of fruit or vegetables each day over the next three months if you make a decision about what you will eat and when and where you will do so. For example, you may find it useful to eat an apple instead of a chocolate bar for your afternoon snack, whilst watching Countdown in the living room. What I would like to do now is to decide with you what you will eat and when and where you will do this over the next 3 months. Let’s first record the times and places that you already eat a portion of fruit or vegetables, then we will make a plan for the 2 extra portions each day. [...]” (Jackson et al., 2005)	Situation: “when and where”; times and places (example provides a perceivable cue) Response: extra portion of F/V  Ambiguous Link: example (not in situation-response format); no link-formation instructions
“[…] Intervention participants were then asked to plan and record where and when they would buy their supply of fruit and vegetables for the following week. Space was provided for them to write down (a) the place of purchase, and (b) the day of purchase. Participants were also asked to plan meals that would allow them to consume the recommended daily intake of fruit and vegetables and to describe these meals under the headings, ‘fruit and vegetables at lunchtime’, and ‘fruit and vegetables in the evening meal’. […]” (Kellar & Abraham, 2005, p. 548)	Situation(1): place of purchase (perceivable cues) Response(1): buying F/V (goal-directed response)  Ambiguous Situation(2): day of purchase (”calendar” planning) Response(2): plan meals (~choice) Link: no link-formation instructions
“[…] Please be specific about which meal or snack the substitution will be made each day. 1) What types of food, food additions or beverage can you do without or eat less of so that you eat more fruit and vegetables? Food item(s): When you usually eat it: [...] How you will eat less of it: [...] 2) When can you fit in at least 1 extra serving of vegetables [...] each day? Please be specific about what time(s) of day, snack or meal you can substitute in an extra serving of vegetables. [Repeated once with focus on fruits]” (Djuric et al., 2010, p. 269)	Response: How to eat less of it (potentially appropriate behavioral response)  Ambiguous Situation: which meal or snack to substitute/ what time of day (mix of potential perceivable cue [old meal/snack] and "calendar" planning [time of day]) Link: Text boxes for different components; no link-formation instructions  Comment: The instructions appear to induce a thorough deliberation of a person’s everyday routines and where to fit in extra portions of fruit and vegetables, but developing clear if-then plans to achieve these changes appear to be missing.
“[…] “Please write in the space below when, where, and how you will eat 5 portions of fruit and vegetables a day.” Trained interviewers answered questions or clarified misunderstandings in a nondirectiv manner. […]” (Reuter et al., 2008, p. 201)	Response: how (goal-directed response)  Ambiguous Situation: When and where text boxes (”calendar” planning; no perceivable cues) Link: Text boxes for different components; no link-formation instructions
“[…] Participants received prepared action planning sheets and were asked to generate a detailed plan of when, where, and how they intended to consume fruit and vegetables. They were also asked to specify which particular kind of fruit and vegetables they planned to consume at certain occasions. Moreover, they received prepared coping planning sheets that required them to imagine situations where barriers might emerge that would prevent them from acting as previously planned. In discussion groups, students shared their experience with intentions and barriers, and they learned how to recover from setbacks. Nutrition calendars were introduced to help them practice how to make a weekly plan and a general nutrition planner. […]” (Kreausukon et al., 2012, p. 446)	Situation(1): imagine barriers (perceivable cues)  Ambiguous Situation(2): When and where (”calendar” planning) Response: which kind of F/V (choice, rather than goal-directed response) Link: Text box completion; no link-formation instructions  Comment: There is reference to a nutrition calendar which is closer to the concept of broad action planning than if-then planning.
“[…] Respondents were presented with five action plans for each of the health behaviors and were asked whether they intended to implement these plans (yes/no). Examples of the action plans were ‘‘to go for a brisk walk during lunch,’’ ‘‘to eat fruit at a set time,’’ ‘‘to replace fatty products with low-fat products,’’ ‘‘to eat boiled and raw vegetables at dinner,’’ and ‘‘to remove all smoking materials from the house.’’ [...] (De Vries et al., 2008, p. 419)	Response: ready-made goal-directed responses  Ambiguous Situation: no situations Link: no link formation
“[…] Write down on the next page which day you chose, and formulate in as much detail as possible what exactly you will eat and drink during the specified moments. Thus compose your own menu, and try to do this as realistically as possible'. The form they used for their implementation intentions contained six entries, namely breakfast, in-between breakfast and lunch, lunch, in-between lunch and dinner, dinner, and after-dinner. […]” (Verplanken & Faes, 1999, p. 597-598)	Link: situation-response template  Ambiguous Situation: which day/ each meal on a specific day ("calendar" planning) Response: what exactly you will eat and drink (choices instead of goal-directed responses)
"[...] The if-then and global implementation intention formats were designed to be as similar as possible with regards to information and examples, with the only difference being the instruction for structuring the plans." (page 321)  “ ‘We want you to plan to eat five portions of fruit and vegetables per day during the next week. You are free to choose how you will do this, but we want you to form your plans in as much detail as possible. Please pay particular attention to the situations in which you will implement these plans. You could choose to focus on planning what fruit and vegetables to buy, how you will prepare them, or something more specific. For example, you may find it useful to plan to eat an apple instead of crisps with your lunch at university. Please write your plans in the space provided’ [...]“ (Chapman et al., 2009, “global implementation intention” condition, p. 321).	Situation: Crisps at lunch (example, perceivable cue) Response: what F/V to buy, how to prepare (goal-directed responses)  Ambiguous Link: no link-formation instructions  Comment: The authors highlight this condition as receiving no instructions to structure the plans in an if-then format; in contrast to the same articles included “if-then implementation intention” condition.
“[...] They were further informed that an effective plan contained (a) location, (b) time, (c) old snack they (usually) consumed at that time and place, and (d) new fruit would substitute their old snack. An example of a suitable plan was given (e.g. if I am at home at 20 o'clock in the evening and I feel like having a chocolate candy bar (again), then I will eat an apple instead). Participants were then instructed to formulate such a plan themselves and to consider fruit availability and plan realism when formulating this plan. Participants were also instructed to write down this action plan in an online form on the webpage. In this form, each of the four elements (time; location; old snack; new snacks) was followed by an answering box where the plan elements had to be placed.” (de Bruijn et al., 2017, “action planning” condition, page 164)	Situation(1): location (perceivable cue) Response: new (available) fruit to be eaten (goal-directed response)  Ambiguous Situation(2): time of the day ("calendar" planning) Link: Fill in text boxes (no link-formation instructions)
“[...] 'You are free to choose how you will do this, but we want you to formulate your plans in as much detail as possible. Please pay particular attention to the situations in which you will implement these plans' and participants were left space in which to write their implementation intentions.” (Armitage et al., 2015, “self-generated implementation intention” condition, page 601-602).	Ambiguous Situation: relevance of situation highlighted but no explanation that these should be perceivable cues rather than day/time. Response: Response alone is referred to as “plan” (not the situation-response combination). Link: no explicit facilitation to link situation and response.


Appendix 5. Forest Plot of Comparisons Between If-then Planning Interventions and Control Conditions on Fruit and Vegetable Consumption with the Original, Reported Standard Deviations.


Note. Forest plot illustrating the pooled mean difference (MD), using a random-effects model (REML), with HKSJ-adjustment. * The respective comparison contains only a fruit consumption measure. ** Indicate the values we suspected to be standard errors; they were transformed into standard deviations for the analysis reported in the main article.


Appendix 6. R script including the meta-analysis data and execution code
library(meta)  ######################### ### Final Manuscript, Main Analysis Data #   ====================================  #         Reference                          |  Code      | N.e  | M.e  | SD.e      | N.c  | M.c  | SD.c    |follow-up  | population      | plan                 | year  | outcome #      ---------------------------------------------------------------------------------------------------------------------------------------------------------------------------- x1  <- c( "Armitage et al. (2015)"           , "Arm2015*" ,  33  , 1.46 , 1.55      ,  23  , 1.25 , 0.91   ,  1       ,"Non.student"    ,"Mix"                  ,"2015" ,"F"    ) x2  <- c( "Chapman et al. (2009)"            , "Cha2009"  , 104  , 4.04 , 1.36      ,  97  , 3.87 , 1.23   ,  0.25    ,"Student"        ,"Self.formulated"      ,"2009" ,"FV"   ) x3  <- c( "Chapman et al. (2010), Comp A"    , "Cha2010A" , 119  , 3.82 , 1.13      , 115  , 3.64 , 1.26   ,  3       ,"Student"        ,"Self.formulated"      ,"2010" ,"FV"   ) x4  <- c( "Chapman et al. (2010), Comp B"    , "Cha2010B" , 115  , 3.59 , 1.36      ,  95  , 3.72 , 1.23   ,  6       ,"Student"        ,"Self.formulated"      ,"2010" ,"FV"   ) x5  <- c( "Chapman et al. (2012)"            , "Cha2012"  , 383  , 2.00 , 0.86      , 127  , 1.74 , 0.80   ,  2       ,"Student"        ,"Self.formulated"      ,"2012" ,"FV"   ) x6  <- c( "de Bruijn et al. (2017)"          , "deB2017*" ,  57  , 1.70    , 1.10      ,  62  , 1.50 , 1.1    ,  0.5     ,"Student"        ,"Self.formulated"      ,"2017" ,"F"    ) x7  <- c( "Guillaumie et al. (2013), Comp A" , "Gui2013A" ,  86  , 4.80 , 2.78      ,  54  , 4.30 , 2.94   , 12  ,"Non.student"    ,"Self.formulated"      ,"2013" ,"FV"   ) x8  <- c( "Guillaumie et al. (2013), Comp B" , "Gui2013B" ,  85  , 5.10 , 2.77      ,  70  , 4.70 , 3.45   , 12       ,"Non.student"    ,"Self.formulated"      ,"2013" ,"FV"   ) x9  <- c( "Stadler et al. (2010)"            , "Sta2010"  , 126  , 4.04 , 4.16      , 129  , 3.33 , 4.41   , 24       ,"Non.student"    ,"Self.formulated"      ,"2010" ,"FV"   ) x10 <- c( "Vézina-Im et al. (2019)"          , "Vez2019"  ,  22  , 6.13 , 1.85      ,  23  , 5.64 , 1.55   ,  6       ,"Mix"            ,"Mix"                  ,"2019" ,"FV"   ) x11 <- c( "Wiedemann et al. (2011)"          , "Wie2011"  , 334  , 4.50    , 1.50      ,  40  , 3.60 , 1.3    ,  1       ,"Non.student"    ,"Self.formulated"      ,"2011" ,"FV"   ) x12 <- c( "Tapper et al. (2014)"             , "Tap2014"  ,  50  , 3.90    , 1.60      ,  50  , 3.30 , 1.5    ,  4       ,"Non.student"    ,"Self.formulated"      ,"2014" ,"FV"   ) #      ---------------------------------------------------------------------------------------------------------------------------------------------------------------------------   source.df <- as.data.frame( rbind( x1, x2, x3, x4, x5, x6, x7, x8, x9, x10, x11, x12 ) ) names(source.df) <- c("ReferenceFull","Author","n.e","mean.e","sd.e",                       "n.c","mean.c", "sd.c", "Length_follow_up", "sample",                       "plans", "year","Outcome") source.df$n.e    <- as.numeric( source.df$n.e ) source.df$mean.e <- as.numeric( source.df$mean.e ) source.df$sd.e   <- as.numeric( source.df$sd.e ) source.df$n.c    <- as.numeric( source.df$n.c ) source.df$mean.c <- as.numeric( source.df$mean.c ) source.df$sd.c   <- as.numeric( source.df$sd.c ) str(source.df)  ########################################  # Meta analysis --------------------------------------------------------------- # Use metcont to pool results (REML). m.cont <- metacont(n.e = n.e,                    mean.e = mean.e,                    sd.e = sd.e,                    n.c = n.c,                    mean.c = mean.c,                    sd.c = sd.c,                    studlab = Author,                    data = source.df,                    prediction=TRUE,                    sm = "MD",        #sm = "SMD" # for standardized effect                    common = FALSE,                    random = TRUE,                    method.tau = "REML",    # Restricted maximum-                                            # likelihood estimator [43]                                            # (method.tau="REML")                    method.random.ci = TRUE,   #hakn = TRUE,                    title = "Effect of if-then plans on FV consumption")  summary(m.cont)  # Forest plot  ---------------------------------------------------------------- forest(m.cont,             #layout = "RevMan5",             sortvar = TE,             prediction = TRUE,             print.I2 = TRUE,             print.tau2 = TRUE,             text.random = "Random effects model (HK)",             label.left = "Favours control",             label.right = "Favours if-then planning",             smlab = "Mean difference (MD) \n(portions per day)",                    xlim = c(-1,2.5),             col.diamond = "black",              col.predict = "grey70",             col.square = "grey70" )    # Contour funnel plot --------------------------------------------------------- ## Define colours for contour col.contour = c("gray75", "gray85", "gray95")  ## Make funnel plot  funnel(m.cont,             xlim = c(-0.5, 1.0),             studlab = TRUE,             contour.levels = c(0.9, 0.95, 0.99),             col.contour = col.contour)  ## Add legend legend(x = 0.65, y = 0.01,        legend = c("p < 0.1", "p < 0.05", "p < 0.01"),        fill = col.contour)   # Linear regression test of funnel plot asymmetry ------- metabias(m.cont, method.bias = "linreg")   #################################### # None-adjusted original data ####### #         Reference                          |  Code      | N.e  | M.e  | SD.e      | N.c  | M.c  | SD.c    |follow-up  | population    | plan              | year  | outcome #      ---------------------------------------------------------------------------------------------------------------------------------------------------------------------- #x1  <- c( "Armitage et al. (2015)"           , "Arm2015*" ,  33  , 1.46 , 0.27    ,  23  , 1.25  , 0.19  ,  1      ,"Non.student" ,"Mix"               ,"2015" ,"F"    ) #x2  <- c( "Chapman et al. (2009)"            , "Cha2009"  , 104  , 4.04 , 1.36    ,  97  , 3.87  , 1.23  ,  0.25   ,"Student"     ,"Self.formulated"   ,"2009" ,"FV"   ) #x3  <- c( "Chapman et al. (2010), Comp A"    , "Cha2010A" , 119  , 3.82 , 1.13    , 115  , 3.64  , 1.26  ,  3      ,"Student"     ,"Self.formulated"   ,"2010" ,"FV"   ) #x4  <- c( "Chapman et al. (2010), Comp B"    , "Cha2010B" , 115  , 3.59 , 1.36    ,  95  , 3.72  , 1.23  ,  6      ,"Student"       ,"Self.formulated"   ,"2010" ,"FV"   ) #x5  <- c( "Chapman et al. (2012)"            , "Cha2012"  , 383  , 2.00 , 0.86    , 127  , 1.74  , 0.80  ,  2      ,"Student"       ,"Self.formulated"   ,"2012" ,"FV"   ) #x6  <- c( "de Bruijn et al. (2017)"          , "deB2017*" ,  57  , 1.70   , 1.10    ,  62  , 1.50  , 1.1   ,  0.5    ,"Student"       ,"Self.formulated"   ,"2017" ,"F"    ) #x7  <- c( "Guillaumie et al. (2013), Comp A" , "Gui2013A" ,  86  , 4.80 , 0.30    ,  54  , 4.30  , 0.40  , 12      ,"Non.student" ,"Self.formulated"   ,"2013" ,"FV"   ) #x8  <- c( "Guillaumie et al. (2013), Comp B" , "Gui2013B" ,  85  , 5.10 , 0.30    ,  70  , 4.70  , 0.40  , 12      ,"Non.student" ,"Self.formulated"   ,"2013" ,"FV"   ) #x9  <- c( "Stadler et al. (2010)"            , "Sta2010"  , 126  , 4.04 , 4.16    , 129  , 3.33  , 4.41  , 24      ,"Non.student" ,"Self.formulated"   ,"2010" ,"FV"   ) #x10 <- c( "Vézina-Im et al. (2019)"          , "Vez2019"  ,  22  , 6.13 , 1.85    ,  23  , 5.64  , 1.55  ,  6      ,"Mix"           ,"Mix"       ,"2019" ,"FV"   ) #x11 <- c( "Wiedemann et al. (2011)"          , "Wie2011"  , 334  , 4.50   , 1.50    ,  40  , 3.60  , 1.3   ,  1      ,"Non.student" ,"Self.formulated"   ,"2011" ,"FV"   ) #x12 <- c( "Tapper et al. (2014)"             , "Tap2014"  ,  50  , 3.90   , 1.60    ,  50  , 3.30  , 1.5   ,  4      ,"Non.student"   ,"Self.formulated"   ,"2014"  ,"FV"   ) #      ---------------------------------------------------------------------------------------------------------------------------------------------------------------------- # -> names(source.df) <- [reuse previous code]   #################################### ### High risk articles removed #   -------------------------- #         Reference                          |  Code      | N.e  | M.e  | SD.e      | N.c  | M.c  | SD.c    |follow-up  | population      | plan                 | year  | outcome #      ----------------------------------------------------------------------------------------------------------------------------------------------------------------------------  x1  <- c( "Armitage et al. (2015)"           , "Arm2015*" ,  33  , 1.46 , 1.55      ,  23  , 1.25 , 0.91   ,  1       ,"Non.student"    ,"Mix"                  ,"2015" ,"F"    ) #x2  <- c( "Chapman et al. (2009)"            , "Cha2009"  , 104  , 4.04 , 1.36      ,  97  , 3.87 , 1.23   ,  0.25    ,"Student"        ,"Self.formulated"      ,"2009" ,"FV"   )  x3  <- c( "Chapman et al. (2010), Comp A"    , "Cha2010A" , 119  , 3.82 , 1.13      , 115  , 3.64 , 1.26   ,  3       ,"Student"        ,"Self.formulated"      ,"2010" ,"FV"   )  x4  <- c( "Chapman et al. (2010), Comp B"    , "Cha2010B" , 115  , 3.59 , 1.36      ,  95  , 3.72 , 1.23   ,  6       ,"Student"        ,"Self.formulated"      ,"2010" ,"FV"   )  x5  <- c( "Chapman et al. (2012)"            , "Cha2012"  , 383  , 2.00 , 0.86      , 127  , 1.74 , 0.80   ,  2       ,"Student"        ,"Self.formulated"      ,"2012" ,"FV"   ) #x6  <- c( "de Bruijn et al. (2017)"          , "deB2017*" ,  57  , 1.70   , 1.10      ,  62  , 1.50 , 1.1    ,  0.5     ,"Student"        ,"Self.formulated"      ,"2017" ,"F"    )  x7  <- c( "Guillaumie et al. (2013), Comp A" , "Gui2013A" ,  86  , 4.80 , 2.78      ,  54  , 4.30 , 2.94   , 12  ,"Non.student"    ,"Self.formulated"      ,"2013" ,"FV"   )  x8  <- c( "Guillaumie et al. (2013), Comp B" , "Gui2013B" ,  85  , 5.10 , 2.77      ,  70  , 4.70 , 3.45   , 12       ,"Non.student"    ,"Self.formulated"      ,"2013" ,"FV"   )  x9  <- c( "Stadler et al. (2010)"            , "Sta2010"  , 126  , 4.04 , 4.16      , 129  , 3.33 , 4.41   , 24       ,"Non.student"    ,"Self.formulated"      ,"2010" ,"FV"   )  x10 <- c( "Vézina-Im et al. (2019)"          , "Vez2019"  ,  22  , 6.13 , 1.85      ,  23  , 5.64 , 1.55   ,  6       ,"Mix"            ,"Mix"                  ,"2019" ,"FV"   )  x11 <- c( "Wiedemann et al. (2011)"          , "Wie2011"  , 334  , 4.50   , 1.50      ,  40  , 3.60 , 1.3    ,  1       ,"Non.student"    ,"Self.formulated"      ,"2011" ,"FV"   )  x12 <- c( "Tapper et al. (2014)"             , "Tap2014"  ,  50  , 3.90   , 1.60      ,  50  , 3.30 , 1.5    ,  4       ,"Non.student"    ,"Self.formulated"      ,"2014" ,"FV"   ) #      ---------------------------------------------------------------------------------------------------------------------------------------------------------------------------  source.df <- as.data.frame( rbind( x1,                                    #x2,                                     x3, x4, x5,                                    #x6,                                     x7, x8, x9, x10, x11, x12 ) ) # -> names(source.df) <- [reuse previous code]


Appendix 7.
List of Excluded Studies and the Central Reason for Exclusion.
Wrong outcome (44)
Count	Citation	Reason(s) for exclusion
1	Shreedhar G, Galizzi MM. Personal or planetary health? Direct, spillover and carryover effects of non-monetary benefits of vegetarian behaviour. J Environ Psychol. 2021;78.	Wrong outcome: number of vegetarian days
2	Knauper B, Carriere K, Frayn M, Ivanova E, Xu Z, Ames-Bull A, et al. The Effects of If-Then Plans on Weight Loss: Results of the McGill CHIP Healthy Weight Program Randomized Controlled Trial. Obesity. 2018;26(8):1285-95.	Wrong outcome: weight loss, fat- and caloric intake
3	Achtziger A, Glas A, Kenning P, Rudolph T. Comparing the effects of financial incentives and implementation intentions on unhealthy snacking behavior in employees. Curr Psychol. 2021;40(10):4770-84.	Wrong outcome: unhealthy snacking behaviour
4	Bradbury D, Upsher R, Chilcot J. A pilot randomised test of a self-affirmation implementation intention intervention to reduce dietary salt intake. Journal of health psychology. 2018;23(6):765-75.	Wrong outcome: salt intake
5	Prestwich A, Ayres K, Lawton R. Crossing two types of implementation intentions with a protection motivation intervention for the reduction of saturated fat intake: A randomized trial. Soc Sci Med. 2008;67(10):1550-8.	Wrong outcome: fat intake
6	Ayre J, Bonner C, Cvejic E, McCaffery K. Randomized trial of planning tools to reduce unhealthy snacking: Implications for health literacy. PLoS One. 2019;14(1):e0209863.	Wrong outcome: unhealthy snacking
7	de Freitas Agondi R, Cornelio ME, Rodrigues RCM, Gallani M-C. Implementation Intentions on the Effect of Salt Intake among Hypertensive Women: A Pilot Study. Nursing research and practice. 2014;2014:196410.	Wrong outcome: salt intake
8	Prestwich A, Conner MT, Lawton RJ, Ward JK, Ayres K, McEachan RRC. Partner- and planning-based interventions to reduce fat consumption: randomized controlled trial. British journal of health psychology. 2014;19(1):132-48.	Wrong outcome: fat intake
9	Kroese FM, Adriaanse MA, Evers C, De Ridder DTD. "Instant success": Turning temptations into cues for goal-directed behavior. Personality and Social Psychology Bulletin. 2011;37(10):1389-97.	Wrong outcome: chocolate
10	Lacroix K, Gifford R. Targeting interventions to distinct meat-eating groups reduces meat consumption. Food Qual Prefer. 2020;86.	Wrong outcome: meat intake
11	Zandstra EH, den Hoed W, van der Meer N, van der Maas A. Improving compliance to meal-replacement food regimens. Forming implementation intentions (conscious IF-THEN plans) increases compliance. Appetite. 2010;55(3):666-70.	Wrong outcome: meal replacement products
12	van Koningsbruggen GM, Stroebe W, Papies EK, Aarts H. Implementation intentions as goal primes: Boosting self-control in tempting environments. European Journal of Social Psychology. 2011;41(5):551-7.	Wrong outcome: unhealthy food
13	Verhoeven AAC, Adriaanse MA, De Ridder DTD, De Vet E, Fennis BM. Less is more: The effect of multiple implementation intentions targeting unhealthy snacking habits. European Journal of Social Psychology. 2013;43(5):344-54.	Wrong outcome: unhealthy snacking
14	Gregorio-Pascual P, Mahler HIM. Effects of interventions based on the theory of planned behavior on sugar-sweetened beverage consumption intentions and behavior. Appetite. 2020;145:104491.	Wrong outcome: sugar-sweetened beverage
15	O'Connor DB, Armitage CJ, Ferguson E. Randomized test of an implementation intention-based tool to reduce stress-induced eating. Annals of behavioral medicine : a publication of the Society of Behavioral Medicine. 2015;49(3):331-43.	Wrong outcome: stress-induced eating
16	Adriaanse MA, Oettingen G, Gollwitzer PM, Hennes EP, De Ridder DTD, De Wit JBF. When planning is not enough: Fighting unhealthy snacking habits by mental contrasting with implementation intentions (MCII). European Journal of Social Psychology. 2010;40(7):1277-93.	Wrong outcome: unhealthy snacking
17	Adriaanse MA, De Ridder DTD, De Wit JBF. Finding the critical cue: Implementation intentions to change one's diet work best when tailored to personally relevant reasons for unhealthy eating. Personality and Social Psychology Bulletin. 2009;35(1):60-71.	Wrong outcome: unspecific healthy snacks
18	Hayes JF, Balantekin KN, Graham AK, Strube MJ, Bickel WK, Wilfley DE. Implementation intentions for weight loss in college students with overweight and obesity: A proof-of-concept randomized controlled trial. Translational Behavioral Medicine. 2021;11(2):359-68.	Wrong outcome: weight loss, calories and HEI-score
19	Achtziger A, Gollwitzer PM, Sheeran P. Implementation intentions and shielding goal striving from unwanted thoughts and feelings. Personality and Social Psychology Bulletin. 2008;34(3):381-93.	Wrong outcome: unhealthy snacking
20	Verhoeven AA, Adriaanse MA, de Vet E, Fennis BM, de Ridder DT. Identifying the 'if' for 'if-then' plans: combining implementation intentions with cue-monitoring targeting unhealthy snacking behaviour. Psychology & health. 2014;29(12):1476-92.	Wrong outcome: snacking intake, calories
21	Adriaanse MA, van Oosten JMF, de Ridder DTD, de Wit JBF, Evers C. Planning what not to eat: Ironic effects of implementation intentions negating unhealthy habits. Personality and Social Psychology Bulletin. 2011;37(1):69-81.	Wrong outcome: unhealthy snacks
22	Loy LS, Wieber F, Gollwitzer PM, Oettingen G. Supporting Sustainable Food Consumption: Mental Contrasting with Implementation Intentions (MCII) Aligns Intentions and Behavior. Frontiers in psychology. 2016;7:607.	Wrong outcome: meat intake
23	Rees JH, Bamberg S, Jager A, Victor L, Bergmeyer M, Friese M. Breaking the habit: On the highly habitualized nature of meat consumption and implementation intentions as one effective way of reducing it. Basic Appl Soc Psych. 2018;40(3):136-47.	Wrong outcome: meat reduction
24	Judah G, Mullan B, Yee M, Johansson L, Allom V, Liddelow C. A Habit-Based Randomised Controlled Trial to Reduce Sugar-Sweetened Beverage Consumption: the Impact of the Substituted Beverage on Behaviour and Habit Strength. International journal of behavioral medicine. 2020;27(6):623-35.	Wrong outcome: sugar-sweetened beverage
25	Knäuper B, Shireen H, Carriere K, Frayn M, Ivanova E, Xu Z, et al. The effects of if-then plans on weight loss: Results of the 24-month follow-up of the McGill CHIP Healthy Weight Program randomized controlled trial. Trials. 2020;21(1):40.	Wrong outcome: weight loss, fat- and caloric intake (linked to Knäuper et al., 2018)
26	Sullivan HW, Rothman AJ. When Planning Is Needed: Implementation Intentions and Attainment of Approach Versus Avoidance Health Goals. Health Psychology. 2008;27(4):438-44.	Wrong outcome (calories, fat)
27	Kothe EJ, Mullan BA. Acceptability of a theory of planned behaviour email-based nutrition intervention. Health promotion international. 2014;29(1):81-90.	Wrong outcome (breakfast)
28	Tam L, Bagozzi RP, Spanjol J. When Planning Is Not Enough: The Self-Regulatory Effect of Implementation Intentions on Changing Snacking Habits. Health Psychology. 2010;29(3):284-92.	Wrong intervention: time-based; wrong outcome: unhealthy snacking
29	Luszczynska A, Scholz U, Sutton S. Planning to change diet: A controlled trial of an implementation intentions training intervention to reduce saturated fat intake among patients after myocardial infarction. J Psychosom Res. 2007;63(5):491-7.	Wrong outcome: fat intake
30	Sheeran P, Orbell S. Implementation intentions and repeated behaviour: Augmenting the predictive validity of the theory of planned behaviour. European Journal of Social Psychology. 1999;29(2-3):349-69.	Wrong outcome: vitamin C pill
31	Armitage CJ. Evidence That Implementation Intentions Reduce Dietary Fat Intake: A Randomized Trial. Health Psychology. 2004;23(3):319-23.	Wrong outcome: fat intake
32	Allan JL, Sniehotta FF, Johnston M. The best laid plans: planning skill determines the effectiveness of action plans and implementation intentions. Annals of behavioral medicine : a publication of the Society of Behavioral Medicine. 2013;46(1):114-20.	Wrong outcome: completion of food diary
33	Adriaanse MA, Gollwitzer PM, de Ridder DTD, de Wit JBF, Kroese FM. Breaking habits with implementation intentions: A test of underlying processes. Personality and Social Psychology Bulletin. 2011;37(4):502-13.	Wrong outcome
34	Hopstock LA, Deraas TS, Henriksen A, Martiny-Huenger T, Grimsgaard S. Changes in adiposity, physical activity, cardiometabolic risk factors, diet, physical capacity and well-being in inactive women and men aged 57-74 years with obesity and cardiovascular risk - A 6-month complex lifestyle intervention with 6-month follow-up. PLoS One. 2021;16(8 August):e0256631.	Wrong outcome
35	Kothe EJ, Mullan BA, Amaratunga R. Randomised controlled trial of a brief theory-based intervention promoting breakfast consumption. Appetite. 2011;56(1):148-55.	Wrong outcome: breakfast consumption
36	Verhoeven AAC, Kindt M, Zomer CL, de Wit S. An experimental investigation of breaking learnt habits with verbal implementation intentions. Acta psychologica. 2018;184:124-36.	Wrong outcome
37	Anderson AS, Dunlop J, Gallant S, Macleod M, Miedzybrodzka Z, Mutrie N, et al. Feasibility study to assess the impact of a lifestyle intervention (a LivingWELL') in people having an assessment of their family history of colorectal or breast cancer. BMJ Open. 2018;8(2):e019410.	Wrong outcome: fibre and fat intake
38	Oh HJ, Larose R. Tell Me a Story About Healthy Snacking and I Will Follow: Comparing the Effectiveness of Self-Generated Versus Message-Aided Implementation Intentions on Promoting Healthy Snacking Habits Among College Students. Health Commun. 2015;30(10):962-74.	Wrong outcome: snack intake
39	Chatzisarantis NLD, Hagger MS, Wang JCK. Evaluating the effects of implementation intention and self-concordance on behavior. British Journal of Psychology. 2010;101(4):705-18.	Wrong outcome: multivitamin
40	Churchill S, Pavey L, Sparks P. The Impact of Autonomy-Framed and Control-Framed Implementation Intentions on Snacking Behaviour: The Moderating Effect of Eating Self-Efficacy. Appl Psychol Health Well Being. 2019;11(1):42-58.	Wrong outcome, snack intake
41	Bagozzi RP, Edwards EA. Goal-striving and the implementation of goal intentions in the regulation of body weight. Special Issue: Methods and Models in Health Psychology. 2000;15(2):255-70. https://doi.org/10.1080/08870440008400305	Wrong outcome
42	Churchill S, Jessop D. Spontaneous implementation intentions and impulsivity: can impulsivity moderate the effectiveness of planning strategies? British journal of health psychology. 2010;15(Pt 3):529-41. https://doi.org/10.1348/135910709X475423	Wrong outcome
43	Brittain M, Consedine N, Bagot KL, Booth N, Rodda SN. Sugar Habit Hacker: Initial evidence that a planning intervention reduces sugar intake. J. 2021;10(3):471-81.	Wrong outcome
44	Vinkers CD, Adriaanse MA, Kroese FM, de Ridder DT. Better sorry than safe: Making a Plan B reduces effectiveness of implementation intentions in healthy eating goals. Psychology & health. 2015;30(7):821-38.	Wrong outcome

 
Passive/no goal/no Control (12)
1 (45)	Armitage CJ. Effects of an implementation intention-based intervention on fruit consumption. Psychology & Health. 2007;22(8):917-28.	Passive control
2 (46)	de Nooijer J, de Vet E, Brug J, de Vries NK. Do Implementation Intentions Help to Turn Good Intentions into Higher Fruit Intakes? J Nutr Educ Behav. 2006;38(1):25-9.	Wrong control (only II had goal)
3 (47)	Lange, D., Richert, J., Koring, M., Knoll, N., Schwarzer, R., & Lippke, S. (2013). Self-regulation prompts can increase fruit consumption: A one-hour randomised controlled online trial. Psychology & Health, 28(5), 533–545. https://doi.org/10.1080/08870446.2012.751107	Wrong control (no goal)
4 (48)	Gohner W, Schlatterer M, Seelig H, Frey I, Berg A, Fuchs R. Two-year follow-up of an interdisciplinary cognitive-behavioral intervention program for obese adults. Journal of Psychology: Interdisciplinary and Applied. 2012;146(4):371-91.wrog https://doi.org/10.1080/00223980.2011.642023	Wrong control
5 (49)	Luszczynska A, Cieslak R. Mediated effects of social support for healthy nutrition: Fruit and vegetable intake across 8 months after myocardial infarction. Behav Med. 2009;35(1):30-8. https://doi.org/10.3200/BMED.35.1.30-38	Wrong control; no comparator condition
6 (50)	Plaete J, De Bourdeaudhuij I, Verloigne M, Crombez G. The use and evaluation of self-regulation techniques can predict health goal attainment in adults: an explorative study. PeerJ. 2016;4:e1666. DOI: 10.7717/peerj.1666	Wrong control; no comparator condition
7 (51)	Epton T, Norman P, Dadzie AS, Harris PR, Webb TL, Sheeran P, et al. A theory-based online health behaviour intervention for new university students (U@Uni): results from a randomised controlled trial. BMC Public Health. 2014;14:563. DOI: 10.1186/1471-2458-14-563	Wrong control
8 (52)	Cameron D, Epton T, Norman P, Sheeran P, Harris PR, Webb TL, et al. A theory-based online health behaviour intervention for new university students (U@Uni: LifeGuide): Results from a repeat randomized controlled trial. Trials. 2015;16(1):555.	Wrong control
9 (53)	Pirolli P, Mohan S, Venkatakrishnan A, Nelson L, Silva M, Springer A. Implementation Intention and Reminder Effects on Behavior Change in a Mobile Health System: A Predictive Cognitive Model. Journal of medical Internet research. 2017;19(11):e397. DOI: 10.2196/jmir.8217	Wrong control; no comparator condition
10 (54)	White SC, Agurto I, Araguas N. Promoting healthy behaviors to prevent chronic disease in Panama and Trinidad & Tobago: Results of the women as agents of change project. Journal of Community Health. 2006;31(5):413-29.	No control
11 (55)	Churchill S, Jessop DC. Too impulsive for implementation intentions? Evidence that impulsivity moderates the effectiveness of an implementation intention intervention. Psychology and Health. 2011;26(5):517-30.	passive control
12 (56)	Gholami, M., Lange, D., Luszczynska, A., Knoll, N., & Schwarzer, R. (2013). A dietary planning intervention increases fruit consumption in Iranian women. Appetite, 63, 1–6. https://doi.org/10.1016/j.appet.2012.12.005	passive control

   
Insufficient statistics (4)
1 (57)	Kendzierski D, Ritter RL, Stump TK, Anglin CL. The effectiveness of an implementation intentions intervention for fruit and vegetable consumption as moderated by self-schema status. Appetite. 2015;95:228-38.	No main effect means
2 (58)	Luszczynska, A., Scholz, U., & Sutton, S. (2007). Planning to change diet: A controlled trial of an implementation intentions training intervention to reduce saturated fat intake among patients after myocardial infarction. Journal of Psychosomatic Research, 63(5), 491–497. https://doi.org/10.1016/j.jpsychores.2007.06.014	Means are only in graph (no stats)
3 (59)	Harris, P. R., Brearley, I., Sheeran, P., Barker, M., Klein, W. M. P., Creswell, J. D., Levine, J. M., & Bond, R. (2014). Combining self-affirmation with implementation intentions to promote fruit and vegetable consumption. Health Psychology, 33(7), 729–736. https://doi.org/10.1037/hea0000065	Means are only presented as standardized values
4 (60)	Knäuper, B., Pillay, R., Lacaille, J., McCollam, A., & Kelso, E. (2011). Replacing craving imagery with alternative pleasant imagery reduces craving intensity. Appetite, 57(1), 173–178. https://doi.org/10.1016/j.appet.2011.04.021	No useable statistics

 
General action planning interventions (11)
1 (61)	Troop NA. Brief report: effect of dietary restraint on fruit and vegetable intake following implementation intentions. Journal of health psychology. 2013;18(7):861-5.	General action planning (see Appendix 2 for details)
2 (62)	Kellar I, Abraham C. Randomized controlled trial of a brief research-based intervention promoting fruit and vegetable consumption. British Journal of Health Psychology. 2005;10(4):543-58.	General action planning (see Appendix 2 for details)
3 (63)	Djuric, Z., Ellsworth, J. S., Ren, J., Sen, A., & Ruffin Iv, M. T. (2010). A randomized feasibility trial of brief telephone counseling to increase fruit and vegetable intakes. Preventive Medicine, 50(5–6), 265–271. https://doi.org/10.1016/j.ypmed.2010.03.003	General action planning (see Appendix 2 for details)
4 (64)	Reuter, T., Ziegelmann, J. P., Wiedemann, A. U., & Lippke, S. (2008). Dietary Planning as a Mediator of the Intention–Behavior Relation: An Experimental‐Causal‐Chain Design. Applied Psychology, 57(s1), 194–207. https://doi.org/10.1111/j.1464-0597.2008.00364.x	General action planning (see Appendix 2 for details)
5 (65)	Kreausukon, P., Gellert, P., Lippke, S., & Schwarzer, R. (2012). Planning and self-efficacy can increase fruit and vegetable consumption: A randomized controlled trial. Journal of Behavioral Medicine, 35(4), 443–451. https://doi.org/10.1007/s10865-011-9373-1	General action planning (see Appendix 2 for details)
6 (66)	De Vries, H., Kremers, S. P. J., Smeets, T., Brug, J., & Eijmael, K. (2008). The Effectiveness of Tailored Feedback and Action Plans in an Intervention Addressing Multiple Health Behaviors. American Journal of Health Promotion, 22(6), 417–424. https://doi.org/10.4278/ajhp.22.6.417	General action planning (see Appendix 2 for details)
7 (67)	Verplanken B, Faes S. Good intentions, bad habits, and effects of forming implementation intentions on healthy eating. European Journal of Social Psychology. 1999;29(5-6):591-604.	General action planning (see Appendix 2 for details)
8 (68)	Luszczynska A, Haynes C. Changing nutrition, physical activity and body weight among student nurses and midwives: Effects of a planning intervention and self-efficacy beliefs. Journal of Health Psychology. 2009;14(8):1075-84.	General action planning (see Appendix 2 for details)
9 (69)	Wiedemann, A. U., Lippke, S., & Schwarzer, R. (2012). Multiple plans and memory performance: Results of a randomized controlled trial targeting fruit and vegetable intake. Journal of Behavioral Medicine, 35(4), 387–392. https://doi.org/10.1007/s10865-011-9364-2	General action planning (see Appendix 2 for details)
10 (70)	Jackson, C., Lawton, R., Knapp, P., Raynor, D. K., Conner, M., Lowe, C., & José Closs, S. (2005). Beyond intention: Do specific plans increase health behaviours in patients in primary care? A study of fruit and vegetable consumption. Social Science & Medicine, 60(10), 2383–2391. https://doi.org/10.1016/j.socscimed.2004.10.014	General action planning (see Appendix 2 for details)

 
 


Appendix 8. PRISMA 2020 Checklist
Section and Topic	Item #	Checklist item	Location where item is reported
TITLE	
Title	1	Identify the report as a systematic review.	p. 1
ABSTRACT	
Abstract	2	See the PRISMA 2020 for Abstracts checklist.	App. X
INTRODUCTION	
Rationale	3	Describe the rationale for the review in the context of existing knowledge.	p. 4-6
Objectives	4	Provide an explicit statement of the objective(s) or question(s) the review addresses.	p. 7
METHODS	
Eligibility criteria	5	Specify the inclusion and exclusion criteria for the review and how studies were grouped for the syntheses.	p. 7,8
Information sources	6	Specify all databases, registers, websites, organisations, reference lists and other sources searched or consulted to identify studies. Specify the date when each source was last searched or consulted.	p. 8
Search strategy	7	Present the full search strategies for all databases, registers and websites, including any filters and limits used.	p. 8, App. 1
Selection process	8	Specify the methods used to decide whether a study met the inclusion criteria of the review, including how many reviewers screened each record and each report retrieved, whether they worked independently, and if applicable, details of automation tools used in the process.	p. 8,9
Data collection process	9	Specify the methods used to collect data from reports, including how many reviewers collected data from each report, whether they worked independently, any processes for obtaining or confirming data from study investigators, and if applicable, details of automation tools used in the process.	p. 9
Data items	10a	List and define all outcomes for which data were sought. Specify whether all results that were compatible with each outcome domain in each study were sought (e.g. for all measures, time points, analyses), and if not, the methods used to decide which results to collect.	p. 9
	10b	List and define all other variables for which data were sought (e.g. participant and intervention characteristics, funding sources). Describe any assumptions made about any missing or unclear information.	p. 9,10
Study risk of bias assessment	11	Specify the methods used to assess risk of bias in the included studies, including details of the tool(s) used, how many reviewers assessed each study and whether they worked independently, and if applicable, details of automation tools used in the process.	p. 10
Effect measures	12	Specify for each outcome the effect measure(s) (e.g. risk ratio, mean difference) used in the synthesis or presentation of results.	p. 10
Synthesis methods	13a	Describe the processes used to decide which studies were eligible for each synthesis (e.g. tabulating the study intervention characteristics and comparing against the planned groups for each synthesis (item #5)).	p. 7-8
	13b	Describe any methods required to prepare the data for presentation or synthesis, such as handling of missing summary statistics, or data conversions.	p. 9,10
	13c	Describe any methods used to tabulate or visually display results of individual studies and syntheses.	p. 10
	13d	Describe any methods used to synthesize results and provide a rationale for the choice(s). If meta-analysis was performed, describe the model(s), method(s) to identify the presence and extent of statistical heterogeneity, and software package(s) used.	p. 10
	13e	Describe any methods used to explore possible causes of heterogeneity among study results (e.g. subgroup analysis, meta-regression).	p. 10
	13f	Describe any sensitivity analyses conducted to assess robustness of the synthesized results.	p. 10
Reporting bias assessment	14	Describe any methods used to assess risk of bias due to missing results in a synthesis (arising from reporting biases).	p. 10
Certainty assessment	15	Describe any methods used to assess certainty (or confidence) in the body of evidence for an outcome.	p. 10
RESULTS	
Study selection	16a	Describe the results of the search and selection process, from the number of records identified in the search to the number of studies included in the review, ideally using a flow diagram.	p. 12
	16b	Cite studies that might appear to meet the inclusion criteria, but which were excluded, and explain why they were excluded.	p. 12,13; App. 2
Study characteristics	17	Cite each included study and present its characteristics.	p. 15
Risk of bias in studies	18	Present assessments of risk of bias for each included study.	p. 19,20; App. 7
Results of individual studies	19	For all outcomes, present, for each study: (a) summary statistics for each group (where appropriate) and (b) an effect estimate and its precision (e.g. confidence/credible interval), ideally using structured tables or plots.	App. 5/6
Results of syntheses	20a	For each synthesis, briefly summarise the characteristics and risk of bias among contributing studies.	p. 19,20
	20b	Present results of all statistical syntheses conducted. If meta-analysis was done, present for each the summary estimate and its precision (e.g. confidence/credible interval) and measures of statistical heterogeneity. If comparing groups, describe the direction of the effect.	p. 18,19
	20c	Present results of all investigations of possible causes of heterogeneity among study results.	p. 19
	20d	Present results of all sensitivity analyses conducted to assess the robustness of the synthesized results.	p. 21,22
Reporting biases	21	Present assessments of risk of bias due to missing results (arising from reporting biases) for each synthesis assessed.	p. 21,22
Certainty of evidence	22	Present assessments of certainty (or confidence) in the body of evidence for each outcome assessed.	p. 21,22
DISCUSSION	
Discussion	23a	Provide a general interpretation of the results in the context of other evidence.	p. 23
	23b	Discuss any limitations of the evidence included in the review.	p. 26
	23c	Discuss any limitations of the review processes used.	p. 27
	23d	Discuss implications of the results for practice, policy, and future research.	p. 25
OTHER INFORMATION	
Registration and protocol	24a	Provide registration information for the review, including register name and registration number, or state that the review was not registered.	N/A p. 7,29
	24b	Indicate where the review protocol can be accessed, or state that a protocol was not prepared.	p. 7,29
	24c	Describe and explain any amendments to information provided at registration or in the protocol.	p. 29
Support	25	Describe sources of financial or non-financial support for the review, and the role of the funders or sponsors in the review.	p. 38
Competing interests	26	Declare any competing interests of review authors.	p. 38
Availability of data, code and other materials	27	Report which of the following are publicly available and where they can be found: template data collection forms; data extracted from included studies; data used for all analyses; analytic code; any other materials used in the review.	Appendices include all available data.


From:  Page MJ, McKenzie JE, Bossuyt PM, Boutron I, Hoffmann TC, Mulrow CD, et al. The PRISMA 2020 statement: an updated guideline for reporting systematic reviews. BMJ 2021;372:n71. doi: 10.1136/bmj.n71. This work is licensed under CC BY 4.0. To view a copy of this license, visit https://creativecommons.org/licenses/by/4.0/ 

 


Appendix 9. PRISMA 2020 for Abstracts Checklist
Section and Topic	Item #	Checklist item	Reported (Yes/No)
TITLE	
Title	1	Identify the report as a systematic review.	Yes
BACKGROUND	
Objectives	2	Provide an explicit statement of the main objective(s) or question(s) the review addresses.	Yes
METHODS	
Eligibility criteria	3	Specify the inclusion and exclusion criteria for the review.	Yes
Information sources	4	Specify the information sources (e.g. databases, registers) used to identify studies and the date when each was last searched.	Yes
Risk of bias	5	Specify the methods used to assess risk of bias in the included studies.	No (details in method section)
Synthesis of results	6	Specify the methods used to present and synthesise results.	Yes
RESULTS	
Included studies	7	Give the total number of included studies and participants and summarise relevant characteristics of studies.	Yes
Synthesis of results	8	Present results for main outcomes, preferably indicating the number of included studies and participants for each. If meta-analysis was done, report the summary estimate and confidence/credible interval. If comparing groups, indicate the direction of the effect (i.e. which group is favoured).	Yes
DISCUSSION	
Limitations of evidence	9	Provide a brief summary of the limitations of the evidence included in the review (e.g. study risk of bias, inconsistency and imprecision).	Yes
Interpretation	10	Provide a general interpretation of the results and important implications.	Yes
OTHER	
Funding	11	Specify the primary source of funding for the review.	Yes
Registration	12	Provide the register name and registration number.	N/A (unpublished protocol accessible)


From:  Page MJ, McKenzie JE, Bossuyt PM, Boutron I, Hoffmann TC, Mulrow CD, et al. The PRISMA 2020 statement: an updated guideline for reporting systematic reviews. BMJ 2021;372:n71. doi: 10.1136/bmj.n71. This work is licensed under CC BY 4.0. To view a copy of this license, visit https://creativecommons.org/licenses/by/4.0/ 


 
