# Supplementary material for: A systematic review and meta-analysis on the effectiveness of if-then plans – in a strict sense – to facilitate fruit and vegetable consumption in adults
Source: Int J Behav Nutr Phys Act. 2026 Apr 15;23:51. doi: 10.1186/s12966-026-01915-y (PMC13195971; doi:10.1186/s12966-026-01915-y)
Supplement: Supplementary file 2 — Supplementary Material 2. [file 12966_2026_1915_MOESM2_ESM.docx]

**Appendix 10: Risk-of-bias assessments (RoB 2)**

**Reference to RoB 2:**

Sterne JAC, Savovic J, Page MJ, Elbers RG, Blencowe NS, Boutron I, et al. RoB 2: a revised tool for assessing risk of bias in randomised trials. BMJ. 2019;366:l4898-l.

**Reference to RoB 2 Template for completion:**

Higgins J, Savović J, Page M, JAC. S. Revised Cochrane risk-of-bias tool for randomized trials (RoB 2) Template for completion: RoB 2 Development Group; 2019. Available from: [https://www.riskofbias.info/welcome/rob-2-0-tool/current-version-of-rob-2](https://www.riskofbias.info/welcome/rob-2-0-tool/current-version-of-rob-2"). (Accessed Sept. 2022)

**Reference to RoB 2 Guideline document**

Higgins J, Savović J, Page M, JAC. S. Revised Cochrane risk-of-bias tool for randomized trials (RoB 2) Full Guideline Document RoB 2: Development Group; 2019. Available from: [https://www.riskofbias.info/welcome/rob-2-0-tool/current-version-of-rob-2](https://www.riskofbias.info/welcome/rob-2-0-tool/current-version-of-rob-2"). (Accessed Sept. 2022)

| **Study details**   \| **Reference** \| Armitage CJ. Field experiment of a very brief worksite intervention to improve nutrition among health care workers. Journal of behavioral medicine. 2015;38(4):599-608. \| \| --- \| --- \|   **Study design**   \| X \| Individually-randomized parallel-group trial \| \| --- \| --- \| \| □ \| Cluster-randomized parallel-group trial \| \| □ \| Individually randomized cross-over (or other matched) trial \|   **For the purposes of this assessment, the interventions being compared are defined as**   \| Experimental: \| If-then plans (VHS) \| Comparator: \| Questionnaire control \| \| --- \| --- \| --- \| --- \|  \| **Specify which outcome is being assessed for risk of bias** \| Fruit intake (FI) (quantity) \| \| --- \| --- \|  \| **Specify the numerical result being assessed.** In case of multiple alternative analyses being presented, specify the numeric result (e.g. RR = 1.52 (95% CI 0.83 to 2.77) and/or a reference (e.g. to a table, figure or paragraph) that uniquely defines the result being assessed. \| Mean FI at 1 month:  M.e = 1.46, article sd.e = 0.27, used sd.e = 1.55  M.c = 1.25, article sd.c = 0.19, used sd.c = 0.91 (article sd.e/c are suspected to be SE instead of SD) \| \| --- \| --- \|   **Is the review team’s aim for this result…?**   \| X \| to assess the effect of *assignment to intervention* (the ‘intention-to-treat’ effect) \| \| --- \| --- \| \| □ \| to assess the effect of *adhering to intervention* (the ‘per-protocol’ effect) \|   **If the aim is to assess the effect of *adhering to intervention***, select the deviations from intended intervention that should be addressed (at least one must be checked):  □ occurrence of non-protocol interventions  □ failures in implementing the intervention that could have affected the outcome  □ non-adherence to their assigned intervention by trial participants  **Which of the following sources were obtained to help inform the risk-of-bias assessment? (tick as many as apply)**  X Journal article(s) with results of the trial  □ Trial protocol  □ Statistical analysis plan (SAP)  □ Non-commercial trial registry record (e.g. ClinicalTrials.gov record)  □ Company-owned trial registry record (e.g. GSK Clinical Study Register record)  □ “Grey literature” (e.g. unpublished thesis)  □ Conference abstract(s) about the trial  □ Regulatory document (e.g. Clinical Study Report, Drug Approval Package)  □ Research ethics application  □ Grant database summary (e.g. NIH RePORTER or Research Councils UK Gateway to Research)  □ Personal communication with trialist  □ Personal communication with the sponsor |
| --- | --- | --- | --- | --- | --- | --- | --- | --- | --- | --- | --- | --- | --- | --- | --- | --- | --- | --- | --- | --- |

**Risk of bias assessment**

Responses underlined in green are potential markers for low risk of bias, and responses in red are potential markers for a risk of bias. Where questions relate only to sign posts to other questions, no formatting is used.

**Domain 1: Risk of bias arising from the randomization process**

| **Signalling questions** | **Comments** | **Response options** |
| --- | --- | --- |
| **1.1 Was the allocation sequence random?** | “All participants received a copy of the baseline questionnaire through the internal mail system. The manipulations were placed at the end of identical-looking questionnaires, which were sorted into random order using a web-based randomizer prior to data collection. Once the baseline questionnaire and manipulations were completed, the participant returned it to the researcher in a sealed envelope via the internal mail system. […] Baseline and follow-up questionnaires were matched using personal codes.” | PY |
| **1.2 Was the allocation sequence concealed until participants were enrolled and assigned to interventions?** |  | PY |
| **1.3 Did baseline differences between intervention groups suggest a problem with the randomization process?** | ‘No’, randomization achieved (all p-values checked > 0.05). | N |
| **Risk-of-bias judgement** | Used algorithm for domain 1. | Low |
| Optional: What is the predicted direction of bias arising from the randomization process? | NA | NA |

**Domain 2: Risk of bias due to deviations from the intended interventions (effect of assignment to intervention)**

| **Signalling questions** | **Comments** | **Response options** |
| --- | --- | --- |
| **2.1. Were participants aware of their assigned intervention during the trial?** | Similar questionnaires (self-guided) given to all participants, except If-then planning manipulation (experimental group only) i.e., participants probably not aware of their assigned intervention. Since intervention was self-guided researchers probably not aware of assignment either. | PN |
| **2.2. Were carers and people delivering the interventions aware of participants' assigned intervention during the trial?** |  | N |
| **2.3. If Y/PY/NI to 2.1 or 2.2: Were there deviations from the intended intervention that arose because of the trial context?** | NA | NA |
| **2.4 If Y/PY to 2.3: Were these deviations likely to have affected the outcome?** | NA | NA |
| **2.5. If Y/PY/NI to 2.4: Were these deviations from intended intervention balanced between groups?** | NA | NA |
| **2.6 Was an appropriate analysis used to estimate the effect of assignment to intervention?** | Data analysed following (m)ITT principles with last observation carried forward. ANCOVAs controlling for baseline FI with condition as between participant variable, time as within participants variable, and FI as dependent variable. Including simple planned contrasts. | Y |
| **2.7 If N/PN/NI to 2.6: Was there potential for a substantial impact (on the result) of the failure to analyse participants in the group to which they were randomized?** | NA | NA |
| **Risk-of-bias judgement** | Used algorithm for domain 2. | Low |
| Optional: What is the predicted direction of bias due to deviations from intended interventions? | NA | NA |

**Domain 3: Missing outcome data**

| **Signalling questions** | **Comments** | **Response options** |
| --- | --- | --- |
| **3.1 Were data for this outcome available for all, or nearly all, participants randomized?** | Outcome data at 1 month available for n=43, compared to baseline randomized (n=56) i.e., ≈ 23 % missing data. According to RoB 2 guide ≥ 95% is considered as ‘nearly all’ and is sufficient (‘all’ = all participants randomized) i.e., data not available for ‘nearly all’ due to small sample size and proportional large ‘missing data’. | N |
| **3.2 If N/PN/NI to 3.1: Is there evidence that the result was not biased by missing outcome data?** | Intention to treat method  Analyses rerun ‘per protocol’ and ‘item imputation’ with “…no substantive differences to the pattern of findings.” No significant differences on baseline variables between dropouts and 1-month follow-up sample (*p* = .91), p. 601. | PY |
| **3.3 If N/PN to 3.2: Could missingness in the outcome depend on its true value?** | NA  NA | NA |
| **3.4 If Y/PY/NI to 3.3: Is it likely that missingness in the outcome depended on its true value?** |  | NA |
| **Risk-of-bias judgement** | Used algorithm for domain 2. | Low |
| Optional: What is the predicted direction of bias due to missing outcome data? | NA | NA |

**Domain 4: Risk of bias in measurement of the outcome**

| **Signalling questions** | **Comments** | **Response options** |
| --- | --- | --- |
| **4.1 Was the method of measuring the outcome inappropriate?** | Using the ‘Fruit section’ of a validated, self-administrated eight-item FFQ (reflecting last 1 month) i.e., Dietary assessment method judged as appropriate for quantity measure. | PN |
| **4.2 Could measurement or ascertainment of the outcome have differed between intervention groups?** | No, both groups received the same questionnaires, except If-then planning manipulation (only experimental group). | N |
| **4.3 If N/PN/NI to 4.1 and 4.2: Were outcome assessors aware of the intervention received by study participants?** | No, outcome assessors = the participant (self-assessment). | N |
| **4.4 If Y/PY/NI to 4.3: Could assessment of the outcome have been influenced by knowledge of intervention received?** | NA  NA | NA |
| **4.5 If Y/PY/NI to 4.4: Is it likely that assessment of the outcome was influenced by knowledge of intervention received?** |  | NA |
| **Risk-of-bias judgement** | Low | Low |
| Optional: What is the predicted direction of bias in measurement of the outcome? | NA | NA |

**Domain 5: Risk of bias in selection of the reported result**

| **Signalling questions** | **Comments** | **Response options** |
| --- | --- | --- |
| **5.1 Were the data that produced this result analysed in accordance with a pre-specified analysis plan that was finalized before unblinded outcome data were available for analysis?** | No protocol or SAP published or reported. Comparing ‘methods’ with reported ‘results’ in published article. ‘Data analysis’ reported in methods section align with results reported in ‘results’ section. | PY |
| **Is the numerical result being assessed likely to have been selected, on the basis of the results, from...** |  |  |
| **5.2. ... multiple eligible outcome measurements (e.g. scales, definitions, time points) within the outcome domain?** | One eligible outcome (FI) collected and *reported* three ways for all groups at comparable timepoints. | PN |
| **5.3 ... multiple eligible analyses of the data?** | Multiple eligible analyses conducted based on each FVI outcome measurement, however all analyses are reported in the article. | PN |
| **Risk-of-bias judgement** | Low | Low |
| Optional: What is the predicted direction of bias due to selection of the reported result? | NA | NA |

**Overall risk of bias**

| **Risk-of-bias judgement** | Judged ‘low’ for all domains i.e., overall risk of bias judged as ‘low’ following criteria outlined in RoB 2 Short version (Cribsheet). | Low |
| --- | --- | --- |
| Optional: What is the overall predicted direction of bias for this outcome? | NA | NA |

| **Study details**   \| **Reference** \| Chapman J, Armitage CJ, Norman P. Comparing implementation intention interventions in relation to young adults' intake of fruit and vegetables. Psychology & health. 2009;24(3):317–32. \| \| --- \| --- \|   **Study design**   \| X \| Individually-randomized parallel-group trial \| \| --- \| --- \| \| □ \| Cluster-randomized parallel-group trial \| \| □ \| Individually randomized cross-over (or other matched) trial \|   **For the purposes of this assessment, the interventions being compared are defined as**   \| Experimental: \| If-then plans (II) \| Comparator: \| Active control \| \| --- \| --- \| --- \| --- \|  \| **Specify which outcome is being assessed for risk of bias** \| FVI at 1 week \| \| --- \| --- \|  \| **Specify the numerical result being assessed.** In case of multiple alternative analyses being presented, specify the numeric result (e.g. RR = 1.52 (95% CI 0.83 to 2.77) and/or a reference (e.g. to a table, figure or paragraph) that uniquely defines the result being assessed. \| Table 1, p. 324  M.e = 4.04, sd.e = 1.36  M.c = 3.87, sd.e = 1.23 \| \| --- \| --- \|   **Is the review team’s aim for this result…?**   \| X \| to assess the effect of *assignment to intervention* (the ‘intention-to-treat’ effect) \| \| --- \| --- \| \| □ \| to assess the effect of *adhering to intervention* (the ‘per-protocol’ effect) \|   **If the aim is to assess the effect of *adhering to intervention***, select the deviations from intended intervention that should be addressed (at least one must be checked):  □ occurrence of non-protocol interventions  □ failures in implementing the intervention that could have affected the outcome  □ non-adherence to their assigned intervention by trial participants  **Which of the following sources were obtained to help inform the risk-of-bias assessment? (tick as many as apply)**  X Journal article(s) with results of the trial  □ Trial protocol  □ Statistical analysis plan (SAP)  □ Non-commercial trial registry record (e.g. ClinicalTrials.gov record)  □ Company-owned trial registry record (e.g. GSK Clinical Study Register record)  □ “Grey literature” (e.g. unpublished thesis)  □ Conference abstract(s) about the trial  □ Regulatory document (e.g. Clinical Study Report, Drug Approval Package)  □ Research ethics application  □ Grant database summary (e.g. NIH RePORTER or Research Councils UK Gateway to Research)  □ Personal communication with trialist  □ Personal communication with the sponsor |
| --- | --- | --- | --- | --- | --- | --- | --- | --- | --- | --- | --- | --- | --- | --- | --- | --- | --- | --- | --- | --- |

**Risk of bias assessment**

Responses underlined in green are potential markers for low risk of bias, and responses in red are potential markers for a risk of bias. Where questions relate only to sign posts to other questions, no formatting is used.

**Domain 1: Risk of bias arising from the randomization process**

| **Signalling questions** | **Comments** | **Response options** |
| --- | --- | --- |
| **1.1 Was the allocation sequence random?** | Incomplete information about randomization and allocation concealment, only “randomized controlled design” and "randomly assigned to conditions".  Randomization process not sufficiently described, unclear whether questionnaires are online or in-class, unclear to what extent participants are aware of other students interventions/how the study has prevented this. | NI |
| **1.2 Was the allocation sequence concealed until participants were enrolled and assigned to interventions?** |  | NI |
| **1.3 Did baseline differences between intervention groups suggest a problem with the randomization process?** | Randomization reported as successful, all ps>0.05 (pre-test planning, FVI, theory of planned behaviour variables, age, gender, ethnicity). | N |
| **Risk-of-bias judgement** | Used algorithm for domain 1. | Some concerns |
| Optional: What is the predicted direction of bias arising from the randomization process? | NA | NA |

****Domain 2: Risk of bias due to deviations from the intended interventions (effect of assignment to intervention)****

| **Signalling questions** | **Comments** | **Response options** |
| --- | --- | --- |
| **2.1. Were participants aware of their assigned intervention during the trial?** | Incomplete information to make a judgement. Based on reported reasons for missing data e.g., “varying attendance levels at lectures”, study probably in class, and paper-and-pencil-based, although these details have not been reported clearly. | NI |
| **2.2. Were carers and people delivering the interventions aware of participants' assigned intervention during the trial?** |  | NI |
| **2.3. If Y/PY/NI to 2.1 or 2.2: Were there deviations from the intended intervention that arose because of the trial context?** | No information reported. | NI |
| **2.4 If Y/PY to 2.3: Were these deviations likely to have affected the outcome?** | NA | NA |
| **2.5. If Y/PY/NI to 2.4: Were these deviations from intended intervention balanced between groups?** | NA | NA |
| **2.6 Was an appropriate analysis used to estimate the effect of assignment to intervention?** | No, analyses “…based on the 300 participants for whom full data were available.”  2x2x3 design ANOVA. Analyses only on participants with baseline and follow-up answers. | N |
| **2.7 If N/PN/NI to 2.6: Was there potential for a substantial impact (on the result) of the failure to analyse participants in the group to which they were randomized?** | ‘Probably no’, large proportion of missing data at follow-up, but no sig. diff. between responders vs. non-responders, and between drop-out rates from all groups. | PN |
| **Risk-of-bias judgement** | Used algorithm for domain 2. | Some concerns |
| Optional: What is the predicted direction of bias due to deviations from intended interventions? | NA | NA |

**Domain 3: Missing outcome data**

| **Signalling questions** | **Comments** | **Response options** |
| --- | --- | --- |
| **3.1 Were data for this outcome available for all, or nearly all, participants randomized?** | No, 54 % of questionnaires completed at 1 week follow up i.e., 46 % missing outcomes. | N |
| **3.2 If N/PN/NI to 3.1: Is there evidence that the result was not biased by missing outcome data?** | No sig. diff. between responders vs. non-responders (all ps>0.05). No sig. diff. between drop-out rates from all groups (all ps>0.05). But no intention-to-treat method. | PN |
| **3.3 If N/PN to 3.2: Could missingness in the outcome depend on its true value?** | NA  NA | NA |
| **3.4 If Y/PY/NI to 3.3: Is it likely that missingness in the outcome depended on its true value?** |  | NA |
| **Risk-of-bias judgement** | Algorithm for domain 3 suggest ‘low’ risk, SKM chose to override this suggestion and judge this domain as ‘some concerns’. Although no sig. difference between drop-out rates and responders vs. non-responders, with 46 % missing outcomes at 1 week cannot rule out that missing outcomes influences the estimated effect of the intervention. | High |
| Optional: What is the predicted direction of bias due to missing outcome data? | NA | NA |

**Domain 4: Risk of bias in measurement of the outcome**

| **Signalling questions** | **Comments** | **Response options** |
| --- | --- | --- |
| **4.1 Was the method of measuring the outcome inappropriate?** | Yes, single-item open ended question about FVI. | PN |
| **4.2 Could measurement or ascertainment of the outcome have differed between intervention groups?** | No, the same outcome measurement method used in all groups at comparable timepoints. | N |
| **4.3 If N/PN/NI to 4.1 and 4.2: Were outcome assessors aware of the intervention received by study participants?** | Self-assessed outcome i.e., outcome assessor = participant. Blinding not described (in-class study), and incomplete information about randomization. | NI |
| **4.4 If Y/PY/NI to 4.3: Could assessment of the outcome have been influenced by knowledge of intervention received?** | It is possible that knowledge of the intervention could affect reported FVI, especially due to study design. But authors did check if participants were aware of study aims, and no significant difference in awareness between groups was found (*p* = .21). | PN |
| **4.5 If Y/PY/NI to 4.4: Is it likely that assessment of the outcome was influenced by knowledge of intervention received?** |  | NA |
| **Risk-of-bias judgement** | Used algorithm for domain 4. | Low |
| Optional: What is the predicted direction of bias in measurement of the outcome? | NA | NA |

**Domain 5: Risk of bias in selection of the reported result**

| **Signalling questions** | **Comments** | **Response options** |
| --- | --- | --- |
| **5.1 Were the data that produced this result analysed in accordance with a pre-specified analysis plan that was finalized before unblinded outcome data were available for analysis?** | No protocol or SAP referenced. Comparing ‘methods’ with ‘results’ in published report. Incomplete information reported on analysis intentions in methods section, all details reported in results. | NI |
| **Is the numerical result being assessed likely to have been selected, on the basis of the results, from...** |  |  |
| **5.2. ... multiple eligible outcome measurements (e.g. scales, definitions, time points) within the outcome domain?** | FVI (outcome) reported at planned follow-up for all groups. | PN |
| **5.3 ... multiple eligible analyses of the data?** | One main analysis reported. | PN |
| **Risk-of-bias judgement** | Used algorithm for domain 5. | Some concerns |
| Optional: What is the predicted direction of bias due to selection of the reported result? | NA | NA |

**Overall risk of bias**

| **Risk-of-bias judgement** | Domain 1, 2, 3 and 5 judged as ‘some concerns’, and domain 4 judged as ‘low’ risk of bias i.e., following RoB 2 suggestion overall risk of bias judged as ‘high’ (due to judged as ‘some concerns’ for four of five domains). | High |
| --- | --- | --- |
| Optional: What is the overall predicted direction of bias for this outcome? | NA | NA |

| **Study details**   \| **Reference** \| Chapman J, Armitage CJ. Evidence that boosters augment the long-term impact of implementation intentions on fruit and vegetable intake. Psychology & health. 2010;25(3):365–81. \| \| --- \| --- \|   **Study design**   \| X \| Individually-randomized parallel-group trial \| \| --- \| --- \| \| □ \| Cluster-randomized parallel-group trial \| \| □ \| Individually randomized cross-over (or other matched) trial \|   **For the purposes of this assessment, the interventions being compared are defined as**   \| Experimental: \| If-then plans \| Comparator: \| Questionnaire control (a) \| \| --- \| --- \| --- \| --- \|  \| **Specify which outcome is being assessed for risk of bias** \| FVI (single-item) at 6 months \| \| --- \| --- \|  \| **Specify the numerical result being assessed.** In case of multiple alternative analyses being presented, specify the numeric result (e.g. RR = 1.52 (95% CI 0.83 to 2.77) and/or a reference (e.g. to a table, figure or paragraph) that uniquely defines the result being assessed. \| M.e = 3.82, sd.e = 1.13 (Comp A)  M.c = 3.64, sd.c = 1.26 (Comp A)  M.e = 3.59, sd.e = 1.36 (Comp B)  M.c = 3.72, sd.c = 1.23 (Comp B) \| \| --- \| --- \|   **Is the review team’s aim for this result…?**   \| X \| to assess the effect of *assignment to intervention* (the ‘intention-to-treat’ effect) \| \| --- \| --- \| \| □ \| to assess the effect of *adhering to intervention* (the ‘per-protocol’ effect) \|   **If the aim is to assess the effect of *adhering to intervention***, select the deviations from intended intervention that should be addressed (at least one must be checked):  □ occurrence of non-protocol interventions  □ failures in implementing the intervention that could have affected the outcome  □ non-adherence to their assigned intervention by trial participants  **Which of the following sources were obtained to help inform the risk-of-bias assessment? (tick as many as apply)**  X Journal article(s) with results of the trial  □ Trial protocol  □ Statistical analysis plan (SAP)  □ Non-commercial trial registry record (e.g. ClinicalTrials.gov record)  □ Company-owned trial registry record (e.g. GSK Clinical Study Register record)  □ “Grey literature” (e.g. unpublished thesis)  □ Conference abstract(s) about the trial  □ Regulatory document (e.g. Clinical Study Report, Drug Approval Package)  □ Research ethics application  □ Grant database summary (e.g. NIH RePORTER or Research Councils UK Gateway to Research)  □ Personal communication with trialist  □ Personal communication with the sponsor |
| --- | --- | --- | --- | --- | --- | --- | --- | --- | --- | --- | --- | --- | --- | --- | --- | --- | --- | --- | --- | --- |

Risk of bias assessment

Responses underlined in green are potential markers for low risk of bias, and responses in red are potential markers for a risk of bias. Where questions relate only to sign posts to other questions, no formatting is used.

**Domain 1: Risk of bias arising from the randomization process**

| **Signalling questions** | **Comments** | **Response options** |
| --- | --- | --- |
| **1.1 Was the allocation sequence random?** | Quote: "Paper-and-pencil questionnaires sorted into random order via a random number generator were distributed at the beginning of the class by individuals who were unaware of the conditions. To reduce the risk of cross-contamination, the questionnaires were completed under examination conditions and participants were requested not to discuss the contents of the questionnaires after completion. At the end of the class, the participants were instructed to place the questionnaires into a collection box." p. 369 | Y |
| **1.2 Was the allocation sequence concealed until participants were enrolled and assigned to interventions?** |  | PY |
| **1.3 Did baseline differences between intervention groups suggest a problem with the randomization process?** | No baseline differences on tested variables (all ps > 0.05). | N |
| **Risk-of-bias judgement** | Used algorithm for domain 1. | Low |
| Optional: What is the predicted direction of bias arising from the randomization process? | NA | NA |

**Domain 2: Risk of bias due to deviations from the intended interventions (effect of assignment to intervention)**

| **Signalling questions** | **Comments** | **Response options** |
| --- | --- | --- |
| **2.1. Were participants aware of their assigned intervention during the trial?** | Participants “…requested to not discuss the contents of the questionnaires after completion.” Thus, possible that participants talk together about the content, since trial is conducted ‘in-class’. But study tested ‘awareness of study hypothesis’, and there was no significant difference between groups, and no significant correlation with FVI.  Intervention was self-guided; internet-based follow up, with contact via (coded) email addresses. | PN |
| **2.2. Were carers and people delivering the interventions aware of participants' assigned intervention during the trial?** |  | PN |
| **2.3. If Y/PY/NI to 2.1 or 2.2: Were there deviations from the intended intervention that arose because of the trial context?** | NA | NA |
| **2.4 If Y/PY to 2.3: Were these deviations likely to have affected the outcome?** | NA | NA |
| **2.5. If Y/PY/NI to 2.4: Were these deviations from intended intervention balanced between groups?** | NA | NA |
| **2.6 Was an appropriate analysis used to estimate the effect of assignment to intervention?** | ITT approach. People lost to follow-up treated as no-changers. ANCOVA analysis, controlling for baseline FVI (single-item measure), and simple contrasts. | Y |
| **2.7 If N/PN/NI to 2.6: Was there potential for a substantial impact (on the result) of the failure to analyse participants in the group to which they were randomized?** | NA | NA |
| **Risk-of-bias judgement** | Used algorithm for domain 2. | Low |
| Optional: What is the predicted direction of bias due to deviations from intended interventions? | NA | NA |

**Domain 3: Missing outcome data**

| **Signalling questions** | **Comments** | **Response options** |
| --- | --- | --- |
| **3.1 Were data for this outcome available for all, or nearly all, participants randomized?** | No. Attrition from baseline to 6- months approx. 40 % missing outcomes. (Fig.1, p. 369) | N |
| **3.2 If N/PN/NI to 3.1: Is there evidence that the result was not biased by missing outcome data?** | Intention-to-treat procedure  and  No significant difference between those who responded and those who did not on all variables at 3- and 6 months, or drop-out rates between conditions (p. 372). Study overpowered (min. n=40 in each group with d=0.65, alpha=0.05, power=0.8).  Incomplete information about reasons for missing outcomes e.g., only “withdrawal from study”. | PY |
| **3.3 If N/PN to 3.2: Could missingness in the outcome depend on its true value?** | NA  NA | NA |
| **3.4 If Y/PY/NI to 3.3: Is it likely that missingness in the outcome depended on its true value?** |  | NA |
| **Risk-of-bias judgement** | Algorithm for domain 3 suggest ‘low’ risk. SKM chose to override this suggestion and judge domain 3 as ‘some concerns’. Although no sig. difference between drop-out rates and responders vs. non-responders, high drop-out rate i.e., 40 % missing outcomes at 6-months. | Low |
| Optional: What is the predicted direction of bias due to missing outcome data? | NA | NA |

**Domain 4: Risk of bias in measurement of the outcome**

| **Signalling questions** | **Comments** | **Response options** |
| --- | --- | --- |
| **4.1 Was the method of measuring the outcome inappropriate?** | No, outcome measured by single-item open ended question and FFQ. | PN |
| **4.2 Could measurement or ascertainment of the outcome have differed between intervention groups?** | Same outcome measurement method used in all groups at comparable timepoints. | N |
| **4.3 If N/PN/NI to 4.1 and 4.2: Were outcome assessors aware of the intervention received by study participants?** | Probably not, outcome assessors = the participant (self-assessed), similar in all study groups. | PN |
| **4.4 If Y/PY/NI to 4.3: Could assessment of the outcome have been influenced by knowledge of intervention received?** | NA  NA | NA |
| **4.5 If Y/PY/NI to 4.4:** **Is it likely that assessment of the outcome was influenced by knowledge of intervention received?** |  | NA |
| **Risk-of-bias judgement** | Used algorithm for domain 4. | Low |
| Optional: What is the predicted direction of bias in measurement of the outcome? | NA | NA |

**Domain 5: Risk of bias in selection of the reported result**

| **Signalling questions** | **Comments** | **Response options** |
| --- | --- | --- |
| **5.1 Were the data that produced this result analysed in accordance with a pre-specified analysis plan that was finalized before unblinded outcome data were available for analysis?** | No protocol or SAP referenced. Comparing ‘methods’ section with ‘results’ section. Incomplete information about analysis intentions. Details on analysis primarily reported in ‘results’ section. | NI |
| **Is the numerical result being assessed likely to have been selected, on the basis of the results, from...** |  |  |
| **5.2. ... multiple eligible outcome measurements (e.g. scales, definitions, time points) within the outcome domain?** | Two outcome measurement methods used, both have been reported for all groups at all pre-specified timepoints. | PN |
| **5.3 ... multiple eligible analyses of the data?** | Not sufficient information about analysis intentions. | NI |
| **Risk-of-bias judgement** | Used algorithm for domain 5. | Some concerns |
| Optional: What is the predicted direction of bias due to selection of the reported result? | NA | NA |

**Overall risk of bias**

| **Risk-of-bias judgement** | Domain 1, 2 and 4 judged as ‘low’ risk of bias, whereas domain 3 and 5 judged as ‘some concerns’ i.e., following RoB 2 suggestion overall risk of bias judged as ‘some concerns’. | Some concerns |
| --- | --- | --- |
| Optional: What is the overall predicted direction of bias for this outcome? | NA | NA |

| **Study details**   \| **Reference** \| Chapman J, Armitage CJ. Do techniques that increase fruit intake also increase vegetable intake? Evidence from a comparison of two implementation intention interventions. Appetite. 2012;58(1):28-33. \| \| --- \| --- \|   **Study design**   \| X \| Individually-randomized parallel-group trial \| \| --- \| --- \| \| □ \| Cluster-randomized parallel-group trial \| \| □ \| Individually randomized cross-over (or other matched) trial \|   **For the purposes of this assessment, the interventions being compared are defined as**   \| Experimental: \| Separate and combined If-then plans (II-S+C) \| Comparator: \| Questionnaire control (AC) \| \| --- \| --- \| --- \| --- \|  \| **Specify which outcome is being assessed for risk of bias** \| Fruit intake (FI) at 2 months \| \| --- \| --- \|  \| **Specify the numerical result being assessed.** In case of multiple alternative analyses being presented, specify the numeric result (e.g. RR = 1.52 (95% CI 0.83 to 2.77) and/or a reference (e.g. to a table, figure or paragraph) that uniquely defines the result being assessed. \| Combined groups. Original and combined values can be found in Appendix Listing 1. \| \| --- \| --- \|   **Is the review team’s aim for this result…?**   \| X \| to assess the effect of *assignment to intervention* (the ‘intention-to-treat’ effect) \| \| --- \| --- \| \| □ \| to assess the effect of *adhering to intervention* (the ‘per-protocol’ effect) \|   **If the aim is to assess the effect of *adhering to intervention***, select the deviations from intended intervention that should be addressed (at least one must be checked):  □ occurrence of non-protocol interventions  □ failures in implementing the intervention that could have affected the outcome  □ non-adherence to their assigned intervention by trial participants  **Which of the following sources were obtained to help inform the risk-of-bias assessment? (tick as many as apply)**  X Journal article(s) with results of the trial  □ Trial protocol  □ Statistical analysis plan (SAP)  □ Non-commercial trial registry record (e.g. ClinicalTrials.gov record)  □ Company-owned trial registry record (e.g. GSK Clinical Study Register record)  □ “Grey literature” (e.g. unpublished thesis)  □ Conference abstract(s) about the trial  □ Regulatory document (e.g. Clinical Study Report, Drug Approval Package)  □ Research ethics application  □ Grant database summary (e.g. NIH RePORTER or Research Councils UK Gateway to Research)  □ Personal communication with trialist  □ Personal communication with the sponsor |
| --- | --- | --- | --- | --- | --- | --- | --- | --- | --- | --- | --- | --- | --- | --- | --- | --- | --- | --- | --- | --- |

**Risk of bias assessment**Responses underlined in green are potential markers for low risk of bias, and responses in red are potential markers for a risk of bias. Where questions relate only to sign posts to other questions, no formatting is used.

**Domain 1: Risk of bias arising from the randomization process**

| **Signalling questions** | **Comments** | **Response options** |
| --- | --- | --- |
| **1.1 Was the allocation sequence random?** | "Randomised controlled design"; “The email contained a link to an online questionnaire, which randomly allocated participants to the control, combined or separate implementation intentions condition.”  1.1 judged ‘yes’ based on description above; 1.2 judged ‘probably yes’ due to assuming online questionnaire function keeping allocation sequence concealed. | Y |
| **1.2 Was the allocation sequence concealed until participants were enrolled and assigned to interventions?** |  | PY |
| **1.3 Did baseline differences between intervention groups suggest a problem with the randomization process?** | ‘Probably no’ as detailed information is missing from article, group characteristics not reported, but overall test of differences between groups where non sig., *p* = .67 (indicating randomization achieved). | PN |
| **Risk-of-bias judgement** | Used algorithm for domain 1. | Low |
| Optional: What is the predicted direction of bias arising from the randomization process? | NA | NA |

**Domain 2: Risk of bias due to deviations from the intended interventions (effect of assignment to intervention)**

| **Signalling questions** | **Comments** | **Response options** |
| --- | --- | --- |
| **2.1. Were participants aware of their assigned intervention during the trial?** | ‘Probably no’ since internet-based intervention, but sample from UK university i.e., could have recruited participants from same study program/class, but incomplete information to make a judgement on this. | PN |
| **2.2. Were carers and people delivering the interventions aware of participants' assigned intervention during the trial?** |  | PN |
| **2.3. If Y/PY/NI to 2.1 or 2.2: Were there deviations from the intended intervention that arose because of the trial context?** | NA | NA |
| **2.4 If Y/PY to 2.3: Were these deviations likely to have affected the outcome?** | NA | NA |
| **2.5. If Y/PY/NI to 2.4: Were these deviations from intended intervention balanced between groups?** | NA | NA |
| **2.6 Was an appropriate analysis used to estimate the effect of assignment to intervention?** | ITT approach (n = 580). “Between-persons ANCOVAs controlling for baseline measures were used to examine the effects of condition (…) on fruit and vegetable intake at follow-up.” Simple contrast analyses. Drop-outs treated as no changers. | Y |
| **2.7 If N/PN/NI to 2.6: Was there potential for a substantial impact (on the result) of the failure to analyse participants in the group to which they were randomized?** | NA | NA |
| **Risk-of-bias judgement** | Used algorithm for domain 2. | Low |
| Optional: What is the predicted direction of bias due to deviations from intended interventions? | NA | NA |

**Domain 3: Missing outcome data**

| **Signalling questions** | **Comments** | **Response options** |
| --- | --- | --- |
| **3.1 Were data for this outcome available for all, or nearly all, participants randomized?** | ‘No’, 68% response rate compared to baseline i.e., 32% missing outcomes at follow-up. | N |
| **3.2 If N/PN/NI to 3.1: Is there evidence that the result was not biased by missing outcome data?** | Reasons for missing outcomes not reported. But “…no significant differences were found between drop-out rates for condition” (*p* = .46), and no sig. diff. between non-responders and responders. No power analysis reported, however, likely oversampled. | PY |
| **3.3 If N/PN to 3.2: Could missingness in the outcome depend on its true value?** | NA  NA | NA |
| **3.4 If Y/PY/NI to 3.3: Is it likely that missingness in the outcome depended on its true value?** |  | NA |
| **Risk-of-bias judgement** | Algorithm for domain 3 suggest ‘low’ risk of bias. SKM chose to override this suggestion and judge risk of bias as ‘some concerns’ based on missing data. Although, no significant differences between non-responders and responders (compared on few characteristics). | Some concerns |
| Optional: What is the predicted direction of bias due to missing outcome data? | NA | NA |

**Domain 4: Risk of bias in measurement of the outcome**

| **Signalling questions** | **Comments** | **Response options** |
| --- | --- | --- |
| **4.1 Was the method of measuring the outcome inappropriate?** | Single-item open ended question about FI and VI, similar questions used in previous research. | PN |
| **4.2 Could measurement or ascertainment of the outcome have differed between intervention groups?** | No, measured with the same method and at comparable timepoints in all groups. | N |
| **4.3 If N/PN/NI to 4.1 and 4.2: Were outcome assessors aware of the intervention received by study participants?** | Probably no, outcome assessor = the participant. Self-reported FI and VI. Assuming participant blinded to intervention due to study design, although not explicitly stated in report. | PN |
| **4.4 If Y/PY/NI to 4.3: Could assessment of the outcome have been influenced by knowledge of intervention received?** | NA  NA | NA |
| **4.5 If Y/PY/NI to 4.4: Is it likely that assessment of the outcome was influenced by knowledge of intervention received?** |  | NA |
| **Risk-of-bias judgement** | Used algorithm for domain 4. | Low |
| Optional: What is the predicted direction of bias in measurement of the outcome? | NA | NA |

**Domain 5: Risk of bias in selection of the reported result**

| **Signalling questions** | **Comments** | **Response options** |
| --- | --- | --- |
| **5.1 Were the data that produced this result analysed in accordance with a pre-specified analysis plan that was finalized before unblinded outcome data were available for analysis?** | No protocol or SAP reported. Comparing ‘methods’ with ‘results’ section. Incomplete information in ‘methods’ section, analysis primarily reported in ‘results’ section i.e., cannot judge if analysis plan was finalised before unblinded outcome data were available based on the available evidence. | NI |
| **Is the numerical result being assessed likely to have been selected, on the basis of the results, from...** |  |  |
| **5.2. ... multiple eligible outcome measurements (e.g. scales, definitions, time points) within the outcome domain?** | FI reported at baseline and 2 months using one method i.e., only one eligible outcome (the same for VI), but not reported combined outcome (FVI). | N |
| **5.3 ... multiple eligible analyses of the data?** | ‘Probably no’, one analysis presented in results. | PN |
| **Risk-of-bias judgement** | Used algorithm for domain 5. | Some concerns |
| Optional: What is the predicted direction of bias due to selection of the reported result? | NA | NA |

**Overall risk of bias**

| **Risk-of-bias judgement** | Judged ‘low’ for domain 1, 2 and 4, and ‘some concerns’ for domain 3 and 5 i.e., overall risk of bias judged as ‘some concerns’ following criteria outlined in RoB 2 Short version (Cribsheet). | Some concerns |
| --- | --- | --- |
| Optional: What is the overall predicted direction of bias for this outcome? | NA | NA |

| **Study details**   \| **Reference** \| De Bruijn, G.-J., Nguyen, M. H., Rhodes, R. E., & Van Osch, L. (2017). Effects of preparatory and action planning instructions on situation-specific and general fruit and snack intake. *Appetite, 108*, 161–170. https://doi.org/10.1016/j.appet.2016.09.016 \| \| --- \| --- \|   **Study design**   \| X \| Individually-randomized parallel-group trial \| \| --- \| --- \| \| □ \| Cluster-randomized parallel-group trial \| \| □ \| Individually randomized cross-over (or other matched) trial \|   **For the purposes of this assessment, the interventions being compared are defined as**   \| Experimental: \| Intervention (preparatory planning) \| Comparator: \| Standard information control condition \| \| --- \| --- \| --- \| --- \|  \| **Specify which outcome is being assessed for risk of bias** \| T2 (2 weeks) F intake (2 weeks) \| \| --- \| --- \|  \| **Specify the numerical result being assessed.** In case of multiple alternative analyses being presented, specify the numeric result (e.g. RR = 1.52 (95% CI 0.83 to 2.77) and/or a reference (e.g. to a table, figure or paragraph) that uniquely defines the result being assessed. \| M.e .= 1.7 (SD.e = 1.1)  M.c. = 1.5 (SD.c = 1.1) \| \| --- \| --- \|   **Is the review team’s aim for this result…?**   \| x□ \| to assess the effect of *assignment to intervention* (the ‘intention-to-treat’ effect) \| \| --- \| --- \| \| □ \| to assess the effect of *adhering to intervention* (the ‘per-protocol’ effect) \|   **If the aim is to assess the effect of *adhering to intervention***, select the deviations from intended intervention that should be addressed (at least one must be checked):  □ occurrence of non-protocol interventions  □ failures in implementing the intervention that could have affected the outcome  □ non-adherence to their assigned intervention by trial participants  **Which of the following sources were obtained to help inform the risk-of-bias assessment? (tick as many as apply)**  □x Journal article(s) with results of the trial  □ Trial protocol  □ Statistical analysis plan (SAP)  □ Non-commercial trial registry record (e.g. ClinicalTrials.gov record)  □ Company-owned trial registry record (e.g. GSK Clinical Study Register record)  □ “Grey literature” (e.g. unpublished thesis)  □ Conference abstract(s) about the trial  □ Regulatory document (e.g. Clinical Study Report, Drug Approval Package)  □ Research ethics application  □ Grant database summary (e.g. NIH RePORTER or Research Councils UK Gateway to Research)  □ Personal communication with trialist  □ Personal communication with the sponsor |
| --- | --- | --- | --- | --- | --- | --- | --- | --- | --- | --- | --- | --- | --- | --- | --- | --- | --- | --- | --- | --- |

## Risk of bias assessment

Responses underlined in green are potential markers for low risk of bias, and responses in red are potential markers for a risk of bias. Where questions relate only to sign posts to other questions, no formatting is used.

**Domain 1: Risk of bias arising from the randomization process**

| **Signalling questions** | **Comments** | **Response options** |
| --- | --- | --- |
| **1.1 Was the allocation sequence random?** | Yes. Quote: “…using simple randomization” (p. 164).  Yes, online intervention, participants are assigned to groups in the online software. | Y |
| **1.2 Was the allocation sequence concealed until participants were enrolled and assigned to interventions?** |  | PY |
| **1.3 Did baseline differences between intervention groups suggest a problem with the randomization process?** | No (baseline values are the same for FI M.e. 1.7 (1.1), M.c. 1.6 (1.1). “Randomization successful” (p. 165). | N |
| **Risk-of-bias judgement** |  | Low |
| Optional: What is the predicted direction of bias arising from the randomization process? |  | NA |

Domain 2: Risk of bias due to deviations from the intended interventions (effect of assignment to intervention)

| **Signalling questions** | **Comments** | **Response options** |
| --- | --- | --- |
| **2.1. Were participants aware of their assigned intervention during the trial?** | Probably not.  Probably not, because it was given as an online intervention. | PN |
| **2.2. Were carers and people delivering the interventions aware of participants' assigned intervention during the trial?** |  | PN |
| **2.3. If Y/PY/NI to 2.1 or 2.2: Were there deviations from the intended intervention that arose because of the trial context?** |  | NA |
| **2.4 If Y/PY to 2.3: Were these deviations likely to have affected the outcome?** |  | NA |
| **2.5. If Y/PY/NI to 2.4: Were these deviations from intended intervention balanced between groups?** |  | NA |
| **2.6 Was an appropriate analysis used to estimate the effect of assignment to intervention?** |  | PY |
| **2.7 If N/PN/NI to 2.6: Was there potential for a substantial impact (on the result) of the failure to analyse participants in the group to which they were randomized?** |  | NA |
| **Risk-of-bias judgement** | Low | Low |
| Optional: What is the predicted direction of bias due to deviations from intended interventions? |  | NA |

Domain 3: Missing outcome data

| **Signalling questions** | **Comments** | **Response options** |
| --- | --- | --- |
| **3.1 Were data for this outcome available for all, or nearly all, participants randomized?** | 164 participants analysed at baseline, 119 participants at follow-up. Attrition approx. 30 %. | N |
| **3.2 If N/PN/NI to 3.1: Is there evidence that the result was not biased by missing outcome data?** | No clear evidence that it is not biased by missing data. | PN |
| **3.3 If N/PN to 3.2: Could missingness in the outcome depend on its true value?** |  | NI |
| **3.4 If Y/PY/NI to 3.3: Is it likely that missingness in the outcome depended on its true value?** |  | NI |
| **Risk-of-bias judgement** |  | High |
| Optional: What is the predicted direction of bias due to missing outcome data? |  | NA |

Domain 4: Risk of bias in measurement of the outcome

| **Signalling questions** | **Comments** | **Response options** |
| --- | --- | --- |
| **4.1 Was the method of measuring the outcome inappropriate?** | No. Used validated questionnaire. | N |
| **4.2 Could measurement or ascertainment of the outcome have differed between intervention groups?** | No. Same method used on all groups. | N |
| **4.3 If N/PN/NI to 4.1 and 4.2: Were outcome assessors aware of the intervention received by study participants?** | No, self-reported. | N |
| **4.4 If Y/PY/NI to 4.3: Could assessment of the outcome have been influenced by knowledge of intervention received?** |  | NA |
| **4.5 If Y/PY/NI to 4.4: Is it likely that assessment of the outcome was influenced by knowledge of intervention received?** |  | NA |
| **Risk-of-bias judgement** |  | Low |
| Optional: What is the predicted direction of bias in measurement of the outcome? |  | NA |

Domain 5: Risk of bias in selection of the reported result

| **Signalling questions** | **Comments** | **Response options** |
| --- | --- | --- |
| **5.1 Were the data that produced this result analysed in accordance with a pre-specified analysis plan that was finalized before unblinded outcome data were available for analysis?** | No information. Do not mention a pre-specified plan, protocol or registration. | NI |
| **Is the numerical result being assessed likely to have been selected, on the basis of the results, from...** |  |  |
| **5.2. ... multiple eligible outcome measurements (e.g. scales, definitions, time points) within the outcome domain?** | No | N |
| **5.3 ... multiple eligible analyses of the data?** | No | N |
| **Risk-of-bias judgement** |  | Some concerns |
| Optional: What is the predicted direction of bias due to selection of the reported result? |  | NA |

Overall risk of bias

| **Risk-of-bias judgement** | High risk of bias | High |
| --- | --- | --- |
| Optional: What is the overall predicted direction of bias for this outcome? |  | NA / Favours experimental / Favours comparator / Towards null /Away from null / Unpredictable |

| **Study details**   \| **Reference** \| Guillaumie L, Godin G, Manderscheid JC, Spitz E, Muller L. Self-efficacy and implementation intentions-based interventions on fruit and vegetable intake among adults: impact at 12-month follow-up. Global health promotion. 2013;20(2 Supplement):83-7. \| \| --- \| --- \|   **Study design**   \| X \| Individually-randomized parallel-group trial \| \| --- \| --- \| \| □ \| Cluster-randomized parallel-group trial \| \| □ \| Individually randomized cross-over (or other matched) trial \|   **For the purposes of this assessment, the interventions being compared are defined as**   \| Experimental: \| If-then plans (II) \| Comparator: \| Information control (AC) \| \| --- \| --- \| --- \| --- \|  \| **Specify which outcome is being assessed for risk of bias** \| Fruit and vegetable intake (FVI) at 12 months \| \| --- \| --- \|  \| **Specify the numerical result being assessed.** In case of multiple alternative analyses being presented, specify the numeric result (e.g. RR = 1.52 (95% CI 0.83 to 2.77) and/or a reference (e.g. to a table, figure or paragraph) that uniquely defines the result being assessed. \| M.e = 4.80 (0.30/2.78); M.c = 4.30 (0.40/2.94)  M.e = 5.10 (0.30/2.77); M.c = 4.70 (0.40/3.45)  In brakets (manuscript SD value / adjust SD value) as the manuscript value was suspected to be the Standard Error. \| \| --- \| --- \|   **Is the review team’s aim for this result…?**   \| X \| to assess the effect of *assignment to intervention* (the ‘intention-to-treat’ effect) \| \| --- \| --- \| \| □ \| to assess the effect of *adhering to intervention* (the ‘per-protocol’ effect) \|   **If the aim is to assess the effect of *adhering to intervention***, select the deviations from intended intervention that should be addressed (at least one must be checked):  □ occurrence of non-protocol interventions  □ failures in implementing the intervention that could have affected the outcome  □ non-adherence to their assigned intervention by trial participants  **Which of the following sources were obtained to help inform the risk-of-bias assessment? (tick as many as apply)**  X Journal article(s) with results of the trial  □ Trial protocol  □ Statistical analysis plan (SAP)  □ Non-commercial trial registry record (e.g. ClinicalTrials.gov record)  □ Company-owned trial registry record (e.g. GSK Clinical Study Register record)  □ “Grey literature” (e.g. unpublished thesis)  □ Conference abstract(s) about the trial  □ Regulatory document (e.g. Clinical Study Report, Drug Approval Package)  □ Research ethics application  □ Grant database summary (e.g. NIH RePORTER or Research Councils UK Gateway to Research)  □ Personal communication with trialist  □ Personal communication with the sponsor |
| --- | --- | --- | --- | --- | --- | --- | --- | --- | --- | --- | --- | --- | --- | --- | --- | --- | --- | --- | --- | --- |

**Risk of bias assessment**

Responses underlined in green are potential markers for low risk of bias, and responses in red are potential markers for a risk of bias. Where questions relate only to sign posts to other questions, no formatting is used.

**Domain 1: Risk of bias arising from the randomization process**

| **Signalling questions** | **Comments** | **Response options** |
| --- | --- | --- |
| **1.1 Was the allocation sequence random?** | "During a phone interview conducted by a research assistant (15-minute length), the study was described, inclusion criteria were specified and participants were randomly assigned to one of the study groups."  “…randomised using a computer-generated randomisation list to one of the four groups."  No information provided about allocation sequence concealment. | Y |
| **1.2 Was the allocation sequence concealed until participants were enrolled and assigned to interventions?** |  | NI |
| **1.3 Did baseline differences between intervention groups suggest a problem with the randomization process?** | Sig. diff. for gender *p* < .001, but not correlated to baseline FVI (*p* = 0.28) (judged as compatible with chance). In report covering 3-month follow-up, those who did not complete all questionnaires were more often male and had a mother born abroad. No diff. between baseline variables in analysed participants (n = 163). In report covering 6- and 12-month follow up (n = 291) only sig. diff. for gender (reported). No baseline characteristics table reported. Difference in control (n = 63) vs. If-then plans group (n = 93) size, not reported intended allocation ratio. | NI |
| **Risk-of-bias judgement** | Used algorithm for domain 1. | Some concerns |
| Optional: What is the predicted direction of bias arising from the randomization process? | NA | NA |

**Domain 2: Risk of bias due to deviations from the intended interventions (effect of assignment to intervention)**

| **Signalling questions** | **Comments** | **Response options** |
| --- | --- | --- |
| **2.1. Were participants aware of their assigned intervention during the trial?** | "Participants were blinded to the content of the other interventions…”  "A total of 14 dietitians experienced in health education were randomly assigned to an intervention type, blinded to the content of other interventions [...]" | PN |
| **2.2. Were carers and people delivering the interventions aware of participants' assigned intervention during the trial?** |  | PN |
| **2.3. If Y/PY/NI to 2.1 or 2.2: Were there deviations from the intended intervention that arose because of the trial context?** | NA | NA |
| **2.4 If Y/PY to 2.3: Were these deviations likely to have affected the outcome?** | NA | NA |
| **2.5. If Y/PY/NI to 2.4: Were these deviations from intended intervention balanced between groups?** | NA | NA |
| **2.6 Was an appropriate analysis used to estimate the effect of assignment to intervention?** | “Generalized estimating equations (GEE) with five measurement times were adopted to test the impact of the intervention on FVI.”  ITT approach for 6-month outcome. ‘Nearly all’ participants included in analysis, n= 291, and efforts done to impute missing data (risk not evaluated in this domain). | PY |
| **2.7 If N/PN/NI to 2.6: Was there potential for a substantial impact (on the result) of the failure to analyse participants in the group to which they were randomized?** | NA | NA |
| **Risk-of-bias judgement** | Used algorithm for domain 2. | Low |
| Optional: What is the predicted direction of bias due to deviations from intended interventions? | NA | NA |

**Domain 3: Missing outcome data**

| **Signalling questions** | **Comments** | **Response options** |
| --- | --- | --- |
| **3.1 Were data for this outcome available for all, or nearly all, participants randomized?** | No, 39 % total missing outcomes at 6 months. | N |
| **3.2 If N/PN/NI to 3.1: Is there evidence that the result was not biased by missing outcome data?** | “Prior to performing these analyses, we used multiple imputation procedures (PROCMI and MIANALYZE in SAS software) to deal with missing data.”  FVI (all timepoints) = imputed variable. Predictors: sociodemographic-, behavioural-, psychosocial variables (i.e., all variables available). “Missing data assumed missing at random.” | PY |
| **3.3 If N/PN to 3.2: Could missingness in the outcome depend on its true value?** | NA  NA | NA |
| **3.4 If Y/PY/NI to 3.3: Is it likely that missingness in the outcome depended on its true value?** |  | NA |
| **Risk-of-bias judgement** | Algorithm for domain 3 suggest ‘low’, but reviewer chose to override this suggestion and judge risk of bias as ‘some concerns’ for this domain. Although imputation procedures have been performed, missing outcomes are very high (39 %), and authors have reported missingness for the overall study (four groups) and not included participant flow diagram for 6- and 12- month outcome. | Some concerns |
| Optional: What is the predicted direction of bias due to missing outcome data? | NA | NA |

**Domain 4: Risk of bias in measurement of the outcome**

| **Signalling questions** | **Comments** | **Response options** |
| --- | --- | --- |
| **4.1 Was the method of measuring the outcome inappropriate?** | No, validated self-reported FFQ used for measuring FI, VI and FVI. | N |
| **4.2 Could measurement or ascertainment of the outcome have differed between intervention groups?** | ‘Probably no’, all groups measured by the same outcome method at comparable timepoints. | PN |
| **4.3 If N/PN/NI to 4.1 and 4.2: Were outcome assessors aware of the intervention received by study participants?** | No, participant = outcome assessor (self-reporting); participants were blinded. | N |
| **4.4 If Y/PY/NI to 4.3: Could assessment of the outcome have been influenced by knowledge of intervention received?** | NA  NA | NA |
| **4.5 If Y/PY/NI to 4.4: Is it likely that assessment of the outcome was influenced by knowledge of intervention received?** |  | NA |
| **Risk-of-bias judgement** | Used algorithm for domain 4. | Low |
| Optional: What is the predicted direction of bias in measurement of the outcome? | NA | NA |

**Domain 5: Risk of bias in selection of the reported result**

| **Signalling questions** | **Comments** | **Response options** |
| --- | --- | --- |
| **5.1 Were the data that produced this result analysed in accordance with a pre-specified analysis plan that was finalized before unblinded outcome data were available for analysis?** | No protocol or SAP available. Methods compared with results in both reports. Same analysis method used in both reports, and outcomes reported as “planned” in methods (FI, VI, FVI) for all timepoints. | PY |
| **Is the numerical result being assessed likely to have been selected, on the basis of the results, from...** |  |  |
| **5.2. ... multiple eligible outcome measurements (e.g. scales, definitions, time points) within the outcome domain?** | One outcome available/defined eligible in present review i.e., FVI at 6 months. | N |
| **5.3 ... multiple eligible analyses of the data?** | For 6 months outcome only ITT approach analyses available. | N |
| **Risk-of-bias judgement** | Used algorithm for domain 5. | Low |
| Optional: What is the predicted direction of bias due to selection of the reported result? | NA | NA |

**Overall risk of bias**

| **Risk-of-bias judgement** | Judged ‘low’ for domain 2, 4 and 5, and ‘some concerns’ for domain 1 and 3 i.e., overall risk of bias judged as ‘some concerns’ following criteria outlined in RoB 2 Short version (Cribsheet). | Some concerns |
| --- | --- | --- |
| Optional: What is the overall predicted direction of bias for this outcome? | NA | NA |

| **Study details**   \| **Reference** \| Stadler G, Oettingen G, Gollwitzer PM. Intervention Effects of Information and Self-Regulation on Eating Fruits and Vegetables Over Two Years. Health Psychology. 2010;29(3):274-83. \| \| --- \| --- \|   **Study design**   \| X \| Individually-randomized parallel-group trial \| \| --- \| --- \| \| □ \| Cluster-randomized parallel-group trial \| \| □ \| Individually randomized cross-over (or other matched) trial \|   **For the purposes of this assessment, the interventions being compared are defined as**   \| Experimental: \| Mental contrasting and If-then plans (MCII) \| Comparator: \| Information intervention (AC) \| \| --- \| --- \| --- \| --- \|  \| **Specify which outcome is being assessed for risk of bias** \| Fruit and vegetable intake (FVI) (servings/week) - 24 months follow-up \| \| --- \| --- \|  \| **Specify the numerical result being assessed.** In case of multiple alternative analyses being presented, specify the numeric result (e.g. RR = 1.52 (95% CI 0.83 to 2.77) and/or a reference (e.g. to a table, figure or paragraph) that uniquely defines the result being assessed. \| (original and transformed values are in Appendix Listing 1.) \| \| --- \| --- \|   **Is the review team’s aim for this result…?**   \| X \| to assess the effect of *assignment to intervention* (the ‘intention-to-treat’ effect) \| \| --- \| --- \| \| □ \| to assess the effect of *adhering to intervention* (the ‘per-protocol’ effect) \|   **If the aim is to assess the effect of *adhering to intervention***, select the deviations from intended intervention that should be addressed (at least one must be checked):  □ occurrence of non-protocol interventions  □ failures in implementing the intervention that could have affected the outcome  □ non-adherence to their assigned intervention by trial participants  **Which of the following sources were obtained to help inform the risk-of-bias assessment? (tick as many as apply)**  X Journal article(s) with results of the trial  □ Trial protocol  □ Statistical analysis plan (SAP)  □ Non-commercial trial registry record (e.g. ClinicalTrials.gov record)  □ Company-owned trial registry record (e.g. GSK Clinical Study Register record)  □ “Grey literature” (e.g. unpublished thesis)  □ Conference abstract(s) about the trial  □ Regulatory document (e.g. Clinical Study Report, Drug Approval Package)  □ Research ethics application  □ Grant database summary (e.g. NIH RePORTER or Research Councils UK Gateway to Research)  □ Personal communication with trialist  □ Personal communication with the sponsor |
| --- | --- | --- | --- | --- | --- | --- | --- | --- | --- | --- | --- | --- | --- | --- | --- | --- | --- | --- | --- | --- |

**Risk of bias assessment**

Responses underlined in green are potential markers for low risk of bias, and responses in red are potential markers for a risk of bias. Where questions relate only to sign posts to other questions, no formatting is used.

**Domain 1: Risk of bias arising from the randomization process**

| **Signalling questions** | **Comments** | **Response options** |
| --- | --- | --- |
| **1.1 Was the allocation sequence random?** | "Telephone interviewers allocated the remaining women to the groups according to a computer-generated block-randomization list with block size 3.” p.277. “[...] Single-blinded, longitudinal RCT".  1.2 judged ‘no information’ although using (small) block sizes; last intervention assignment within each block can be predicted, but study used “trained telephone interviewers” i.e., assuming not main research team.  No further information provided in linked article:  DOI: 10.1016/j.amepre.2008.09.021 | Y |
| **1.2 Was the allocation sequence concealed until participants were enrolled and assigned to interventions?** |  | NI |
| **1.3 Did baseline differences between intervention groups suggest a problem with the randomization process?** | No, table 1, p.279. All *p*-values > .05 for comparing baseline group characteristics. | N |
| **Risk-of-bias judgement** | Used algorithm for domain 1. | Low |
| Optional: What is the predicted direction of bias arising from the randomization process? | NA | NA |

**Domain 2: Risk of bias due to deviations from the intended interventions (effect of assignment to intervention)**

| **Signalling questions** | **Comments** | **Response options** |
| --- | --- | --- |
| **2.1. Were participants aware of their assigned intervention during the trial?** | ‘No’, single-blinded study, participants not aware of assigned intervention; experimental intervention compared to active comparator intervention.  ‘Probably yes’, described as single blinded and delivered face-to-face i.e., assuming participants blinded and interventionist/facilitator unblinded. | N |
| **2.2. Were carers and people delivering the interventions aware of participants' assigned intervention during the trial?** |  | PY |
| **2.3. If Y/PY/NI to 2.1 or 2.2: Were there deviations from the intended intervention that arose because of the trial context?** | No deviations described. Scripted intervention (manual), standardized hand-out material, and checklist to maintain intervention fidelity (see ‘Design’ p. 276). | PN |
| **2.4 If Y/PY to 2.3: Were these deviations likely to have affected the outcome?** | NA | NA |
| **2.5. If Y/PY/NI to 2.4: Were these deviations from intended intervention balanced between groups?** | NA | NA |
| **2.6 Was an appropriate analysis used to estimate the effect of assignment to intervention?** | Yes. ITT approach described: mixed-effects model that used all available data, with condition (experimental vs. comparator) as between-persons factor; follow-up time (0,1,2,4,24 months post-intervention) as within-persons factor; FVI at baseline as covariate; and FVI at follow-up as dependent variable. | Y |
| **2.7 If N/PN/NI to 2.6: Was there potential for a substantial impact (on the result) of the failure to analyse participants in the group to which they were randomized?** | NA | NA |
| **Risk-of-bias judgement** | Used algorithm for domain 2. | Low |
| Optional: What is the predicted direction of bias due to deviations from intended interventions? | NA | NA |

**Domain 3: Missing outcome data**

| **Signalling questions** | **Comments** | **Response options** |
| --- | --- | --- |
| **3.1 Were data for this outcome available for all, or nearly all, participants randomized?** | ~50% (266->129) drop-out for 24 months measure.  However, intention-to-treat method, so analyzed dropout was only (266->255) 4%. | PY |
| **3.2 If N/PN/NI to 3.1: Is there evidence that the result was not biased by missing outcome data?** | NA | NA |
| **3.3 If N/PN to 3.2: Could missingness in the outcome depend on its true value?** | NA  NA | NA |
| **3.4 If Y/PY/NI to 3.3: Is it likely that missingness in the outcome depended on its true value?** |  | NA |
| **Risk-of-bias judgement** | Used algorithm for domain 3. | Low |
| Optional: What is the predicted direction of bias due to missing outcome data? | NA | NA |

**Domain 4: Risk of bias in measurement of the outcome**

| **Signalling questions** | **Comments** | **Response options** |
| --- | --- | --- |
| **4.1 Was the method of measuring the outcome inappropriate?** | No. The exact food diary used in study not validated, but rationale for choosing this method and validation of similar diaries for FVI provided (see ‘Measures’, p.278). | PN |
| **4.2 Could measurement or ascertainment of the outcome have differed between intervention groups?** | No, the same method used in both groups and at comparable timepoints. | N |
| **4.3 If N/PN/NI to 4.1 and 4.2: Were outcome assessors aware of the intervention received by study participants?** | No, outcome assessors i.e., the individual participant filled out self-administrated food diaries, and were blinded to intervention assignment; no evidence suggest blinding was compromised. | N |
| **4.4 If Y/PY/NI to 4.3: Could assessment of the outcome have been influenced by knowledge of intervention received?** | NA  NA | NA |
| **4.5 If Y/PY/NI to 4.4: Is it likely that assessment of the outcome was influenced by knowledge of intervention received?** |  | NA |
| **Risk-of-bias judgement** |  | Low |
| Optional: What is the predicted direction of bias in measurement of the outcome? | NA | NA |

**Domain 5: Risk of bias in selection of the reported result**

| **Signalling questions** | **Comments** | **Response options** |
| --- | --- | --- |
| **5.1 Were the data that produced this result analysed in accordance with a pre-specified analysis plan that was finalized before unblinded outcome data were available for analysis?** | No protocol or SAP available. Comparing ‘methods’ with ‘results’ in published report. Data producing results analysed according to plan specified in methods section, but table of effects at each follow-up not reported, means reported in text, and in Fig. 2, p. 280 (both without SDs). | PN |
| **Is the numerical result being assessed likely to have been selected, on the basis of the results, from...** |  |  |
| **5.2. ... multiple eligible outcome measurements (e.g. scales, definitions, time points) within the outcome domain?** | Outcome measured one way (food diaries) and reported at all planned timepoints. | PN |
| **5.3 ... multiple eligible analyses of the data?** | Three main analyses planned in methods, and all reported in results (all answer different research questions). | PN |
| **Risk-of-bias judgement** | Used algorithm for domain 5. | Low |
| Optional: What is the predicted direction of bias due to selection of the reported result? | NA | NA |

**Overall risk of bias**

| **Risk-of-bias judgement** | Overall risk of bias judged as ‘low’ following suggestion by RoB 2 short version (cribsheet), due to all domains judged as ‘low’ risk of bias.. | Low |
| --- | --- | --- |
| Optional: What is the overall predicted direction of bias for this outcome? | NA | NA |

| **Study details**   \| **Reference** \| Vezina-Im LA, Perron J, Lemieux S, Robitaille J. Promoting fruit and vegetable intake in childbearing age women at risk for gestational diabetes mellitus: A randomised controlled trial. Journal of health psychology. 2019;24(5):600-12. \| \| --- \| --- \|   **Study design**   \| X \| Individually-randomized parallel-group trial \| \| --- \| --- \| \| □ \| Cluster-randomized parallel-group trial \| \| □ \| Individually randomized cross-over (or other matched) trial \|   **For the purposes of this assessment, the interventions being compared are defined as**   \| Experimental: \| If-then plans + question behaviour effect intervention (QBE-II) \| Comparator: \| Question behaviour effect intervention (QBE) \| \| --- \| --- \| --- \| --- \|  \| **Specify which outcome is being assessed for risk of bias** \| Fruit and vegetable intake (FVI) (servings/day) \| \| --- \| --- \|  \| **Specify the numerical result being assessed.** In case of multiple alternative analyses being presented, specify the numeric result (e.g. RR = 1.52 (95% CI 0.83 to 2.77) and/or a reference (e.g. to a table, figure or paragraph) that uniquely defines the result being assessed. \| Mean FVI at 6 months:  M.e = 6.13, sd.e = 1.85  M.c = 5.64, sd.c = 1.55 \| \| --- \| --- \|   **Is the review team’s aim for this result…?**   \| X \| to assess the effect of *assignment to intervention* (the ‘intention-to-treat’ effect) \| \| --- \| --- \| \| □ \| to assess the effect of *adhering to intervention* (the ‘per-protocol’ effect) \|   **If the aim is to assess the effect of *adhering to intervention***, select the deviations from intended intervention that should be addressed (at least one must be checked):  □ occurrence of non-protocol interventions  □ failures in implementing the intervention that could have affected the outcome  □ non-adherence to their assigned intervention by trial participants  **Which of the following sources were obtained to help inform the risk-of-bias assessment? (tick as many as apply)**  X Journal article(s) with results of the trial  □ Trial protocol  □ Statistical analysis plan (SAP)  □ Non-commercial trial registry record (e.g. ClinicalTrials.gov record)  □ Company-owned trial registry record (e.g. GSK Clinical Study Register record)  □ “Grey literature” (e.g. unpublished thesis)  □ Conference abstract(s) about the trial  □ Regulatory document (e.g. Clinical Study Report, Drug Approval Package)  □ Research ethics application  □ Grant database summary (e.g. NIH RePORTER or Research Councils UK Gateway to Research)  □ Personal communication with trialist  □ Personal communication with the sponsor |
| --- | --- | --- | --- | --- | --- | --- | --- | --- | --- | --- | --- | --- | --- | --- | --- | --- | --- | --- | --- | --- |

**Risk of bias assessment**

Responses underlined in green are potential markers for low risk of bias, and responses in red are potential markers for a risk of bias. Where questions relate only to sign posts to other questions, no formatting is used.

**Domain 1: Risk of bias arising from the randomization process**

| **Signalling questions** | **Comments** | **Response options** |
| --- | --- | --- |
| **1.1 Was the allocation sequence random?** | "Women were randomly assigned to either the II or QBE group by assigning random numbers from computer-generated random number tables to the treatment conditions." p.602  No information provided about allocation sequence concealment. | Y |
| **1.2 Was the allocation sequence concealed until participants were enrolled and assigned to interventions?** |  | NI |
| **1.3 Did baseline differences between intervention groups suggest a problem with the randomization process?** | No, table 1, p. 605. Group characteristics compared, all p-values > .05. | N |
| **Risk-of-bias judgement** | Algorithm for domain 1 used. | Some concerns |
| Optional: What is the predicted direction of bias arising from the randomization process? | NA | NA |

**Domain 2: Risk of bias due to deviations from the intended interventions (effect of assignment to intervention)**

| **Signalling questions** | **Comments** | **Response options** |
| --- | --- | --- |
| **2.1. Were participants aware of their assigned intervention during the trial?** | Separate protocol not reported. ‘Protocol’ section described in methods, p. 602.  No information describing participant, or researcher blinding. All participants were given identical questionnaires, except for the ‘If-then’ manipulation at baseline (experimental group only). Questionnaires filled out face-to-face with facilitator. Random sample from the local university i.e., assuming participants did not know each other. | PN |
| **2.2. Were carers and people delivering the interventions aware of participants' assigned intervention during the trial?** |  | NI |
| **2.3. If Y/PY/NI to 2.1 or 2.2: Were there deviations from the intended intervention that arose because of the trial context?** | No information. | NI |
| **2.4 If Y/PY to 2.3: Were these deviations likely to have affected the outcome?** | NA | NA |
| **2.5. If Y/PY/NI to 2.4: Were these deviations from intended intervention balanced between groups?** | NA | NA |
| **2.6 Was an appropriate analysis used to estimate the effect of assignment to intervention?** | Effect of intervention on FVI measured using 2 (condition) x 3 (time) repeated-measures mixed model analyses of variance (ANOVAs) i.e., participants analysed in the group they were randomized. No information on ITT approach, or exclusion, but from flow chart (Fig. 1, p. 604) and results (Table 2, p. 606) seems like only those with missing outcomes at 6 months have been excluded. | PY |
| **2.7 If N/PN/NI to 2.6: Was there potential for a substantial impact (on the result) of the failure to analyse participants in the group to which they were randomized?** | Excluded participants ( ≈ 10%, fig. 1, p. 604 and table 2, p. 606) could have had an effect on the estimated results, but outcome is not rare and not related to prognostic factors. Exclusion of participant data and handling of missing information not sufficiently described. | PN |
| **Risk-of-bias judgement** | Algorithm for domain 2 used. | Some concerns |
| Optional: What is the predicted direction of bias due to deviations from intended interventions? | NA | NA |

*****Domain 3: Missing outcome data*****

| **Signalling questions** | **Comments** | **Response options** |
| --- | --- | --- |
| **3.1 Were data for this outcome available for all, or nearly all, participants randomized?** | Outcome data at 6 months available for n = 45, compared to baseline (n = 50), and randomized (n = 56) i.e., ≈ 10% and 20% missing, respectively. According to RoB 2 guide ≥ 95% is considered as ‘nearly all’ and is sufficient (‘all’ = all participants randomized) i.e., data not available for ‘nearly all’ due to small sample size. | PN |
| **3.2 If N/PN/NI to 3.1: Is there evidence that the result was not biased by missing outcome data?** | ≈ Equal proportions of missing data in each group at 6 months. Participants not completing 6-month follow-up were similar to those who completed (all *p*s > 0.05). | PY |
| **3.3 If N/PN to 3.2: Could missingness in the outcome depend on its true value?** | NA  NA | NA |
| **3.4 If Y/PY/NI to 3.3: Is it likely that missingness in the outcome depended on its true value?** |  | NA |
| **Risk-of-bias judgement** | Algorithm for domain 3 used. | Low |
| Optional: What is the predicted direction of bias due to missing outcome data? | NA | NA |

**Domain 4: Risk of bias in measurement of the outcome**

| **Signalling questions** | **Comments** | **Response options** |
| --- | --- | --- |
| **4.1 Was the method of measuring the outcome inappropriate?** | No, validated food frequency questionnaire (FFQ) (baseline and 6-months). | N |
| **4.2 Could measurement or ascertainment of the outcome have differed between intervention groups?** | No, the same measurement of the outcome have been used in both groups at comparable timepoints. | N |
| **4.3 If N/PN/NI to 4.1 and 4.2: Were outcome assessors aware of the intervention received by study participants?** | Outcome assessor = participant. Blinding not explicitly stated in report, but due to study design (randomized trial) and intervention vs. comparator setup (QBE-II vs. QBE), and study participants being ‘randomly’ recruited among students and employees (i.e., not from the same study program) risk of bias judged as ‘probably no’. | PN |
| **4.4 If Y/PY/NI to 4.3: Could assessment of the outcome have been influenced by knowledge of intervention received?** | NA  NA | NA |
| **4.5 If Y/PY/NI to 4.4: Is it likely that assessment of the outcome was influenced by knowledge of intervention received?** |  | NA |
| **Risk-of-bias judgement** | Algorithm for domain 4 used. | Low |
| Optional: What is the predicted direction of bias in measurement of the outcome? | NA | NA |

**Domain 5: Risk of bias in selection of the reported result**

| **Signalling questions** | **Comments** | **Response options** |
| --- | --- | --- |
| **5.1 Were the data that produced this result analysed in accordance with a pre-specified analysis plan that was finalized before unblinded outcome data were available for analysis?** | No protocol or SAP available. Comparing ‘methods’ with ‘results’ in published report p. 603-607. Judged as ‘probably yes’ based on reporting of ‘protocol statistical analysis plan’ in methods, whereby results from main analysis plan are reported in ‘results’. Reporting of ‘post hoc’ analyses in results suggest authors are transparent in their reporting (i.e., open about what was planned and what was post hoc). | PY |
| **Is the numerical result being assessed likely to have been selected, on the basis of the results, from...** |  |  |
| **5.2. ... multiple eligible outcome measurements (e.g. scales, definitions, time points) within the outcome domain?** | Judged as ‘probably no’: FVI outcome reported according to ‘protocol plan’ (from methods section) at all pre-specified timepoints. | PN |
| **5.3 ... multiple eligible analyses of the data?** | Results reported from analyses reported as planned in methods. | PN |
| **Risk-of-bias judgement** | Used algorithm for domain 5. | Low |
| Optional: What is the predicted direction of bias due to selection of the reported result? | NA | NA |

**Overall risk of bias**

| **Risk-of-bias judgement** | Judged ‘low’ for domain 3, 4 and 5, and ‘some concerns’ for domain 1 and 2 i.e., overall risk of bias judged as ‘some concerns’ following criteria outlined in RoB 2 Short version (Cribsheet). | Some concerns |
| --- | --- | --- |
| Optional: What is the overall predicted direction of bias for this outcome? | NA | NA |

| **Study details**   \| **Reference** \| Wiedemann, A. U., Lippke, S., Reuter, T., Ziegelmann, J. P., & Schwarzer, R. (2011). How planning facilitates behaviour change: Additive and interactive effects of a randomized controlled trial. *European Journal of Social Psychology, 41*(1), 42–51. https://doi.org/10.1002/ejsp.724 \| \| --- \| --- \|   **Study design**   \| X \| Individually-randomized parallel-group trial \| \| --- \| --- \| \| □ \| Cluster-randomized parallel-group trial \| \| □ \| Individually randomized cross-over (or other matched) trial \|   **For the purposes of this assessment, the interventions being compared are defined as**   \| Experimental: \| Intervention \| Comparator: \| Active control condition \| \| --- \| --- \| --- \| --- \|  \| **Specify which outcome is being assessed for risk of bias** \| T2 (1 month) \| \| --- \| --- \|  \| **Specify the numerical result being assessed.** In case of multiple alternative analyses being presented, specify the numeric result (e.g. RR = 1.52 (95% CI 0.83 to 2.77) and/or a reference (e.g. to a table, figure or paragraph) that uniquely defines the result being assessed. \| M.e = 4.5 (SD.e = 1.5)  M.c = 3.6 (SD.c = 1.3) \| \| --- \| --- \|   **Is the review team’s aim for this result…?**   \| x□ \| to assess the effect of *assignment to intervention* (the ‘intention-to-treat’ effect) \| \| --- \| --- \| \| □ \| to assess the effect of *adhering to intervention* (the ‘per-protocol’ effect) \|   **If the aim is to assess the effect of *adhering to intervention***, select the deviations from intended intervention that should be addressed (at least one must be checked):  □ occurrence of non-protocol interventions  □ failures in implementing the intervention that could have affected the outcome  □ non-adherence to their assigned intervention by trial participants  **Which of the following sources were obtained to help inform the risk-of-bias assessment? (tick as many as apply)**  □x Journal article(s) with results of the trial  □ Trial protocol  □ Statistical analysis plan (SAP)  □ Non-commercial trial registry record (e.g. ClinicalTrials.gov record)  □ Company-owned trial registry record (e.g. GSK Clinical Study Register record)  □ “Grey literature” (e.g. unpublished thesis)  □ Conference abstract(s) about the trial  □ Regulatory document (e.g. Clinical Study Report, Drug Approval Package)  □ Research ethics application  □ Grant database summary (e.g. NIH RePORTER or Research Councils UK Gateway to Research)  □ Personal communication with trialist  □ Personal communication with the sponsor |
| --- | --- | --- | --- | --- | --- | --- | --- | --- | --- | --- | --- | --- | --- | --- | --- | --- | --- | --- | --- | --- |

## Risk of bias assessment

Responses underlined in green are potential markers for low risk of bias, and responses in red are potential markers for a risk of bias. Where questions relate only to sign posts to other questions, no formatting is used.

**Domain 1: Risk of bias arising from the randomization process**

| **Signalling questions** | **Comments** | **Response options** |
| --- | --- | --- |
| **1.1 Was the allocation sequence random?** | “computer algorithm randomly assigned participants…”  “Participants were blinded to their allocation for the duration of the study.” | Y |
| **1.2 Was the allocation sequence concealed until participants were enrolled and assigned to interventions?** |  | Y |
| **1.3 Did baseline differences between intervention groups suggest a problem with the randomization process?** | No baseline differences (baseline values Int: 3.8 (1.1), Control: 3.7 (1.1).) | N |
| **Risk-of-bias judgement** | Low | Low |
| Optional: What is the predicted direction of bias arising from the randomization process? | NA | NA |

Domain 2: Risk of bias due to deviations from the intended interventions (effect of assignment to intervention)

| **Signalling questions** | **Comments** | **Response options** |
| --- | --- | --- |
| **2.1. Were participants aware of their assigned intervention during the trial?** | No.  No. | N |
| **2.2. Were carers and people delivering the interventions aware of participants' assigned intervention during the trial?** |  | N |
| **2.3. If Y/PY/NI to 2.1 or 2.2: Were there deviations from the intended intervention that arose because of the trial context?** | NA | NA |
| **2.4 If Y/PY to 2.3: Were these deviations likely to have affected the outcome?** | NA | NA |
| **2.5. If Y/PY/NI to 2.4: Were these deviations from intended intervention balanced between groups?** | NA | NA |
| **2.6 Was an appropriate analysis used to estimate the effect of assignment to intervention?** | Yes, intention to treat analysis with rationale for imputing values for drop-out/missing values. | Y |
| **2.7 If N/PN/NI to 2.6: Was there potential for a substantial impact (on the result) of the failure to analyse participants in the group to which they were randomized?** | NA | NA |
| **Risk-of-bias judgement** | Low | Low |
| Optional: What is the predicted direction of bias due to deviations from intended interventions? |  | NA |

Domain 3: Missing outcome data

| **Signalling questions** | **Comments** | **Response options** |
| --- | --- | --- |
| **3.1 Were data for this outcome available for all, or nearly all, participants randomized?** | No, attrition rate was high (approx. 50 %). | N |
| **3.2 If N/PN/NI to 3.1: Is there evidence that the result was not biased by missing outcome data?** | Yes, used intention-to-treat analyses and included all randomized participants. | Y |
| **3.3 If N/PN to 3.2: Could missingness in the outcome depend on its true value?** | NA  NA | NA |
| **3.4 If Y/PY/NI to 3.3: Is it likely that missingness in the outcome depended on its true value?** |  | NA |
| **Risk-of-bias judgement** |  | Low |
| Optional: What is the predicted direction of bias due to missing outcome data? |  | NA |

Domain 4: Risk of bias in measurement of the outcome

| **Signalling questions** | **Comments** | **Response options** |
| --- | --- | --- |
| **4.1 Was the method of measuring the outcome inappropriate?** | No. Used the sum score of two open ended questions. Quote: “slightly adapted version of an English measure … validated against biomarkers” (reference: Steptoe, Perkins-Porras, et al. 2003, p. 46) | N |
| **4.2 Could measurement or ascertainment of the outcome have differed between intervention groups?** | No, self-reported outcome measurement. The same method used in both groups. | N |
| **4.3 If N/PN/NI to 4.1 and 4.2: Were outcome assessors aware of the intervention received by study participants?** | No, outcome assessor was the participant themselves (self-reported), and they were blinded. | N |
| **4.4 If Y/PY/NI to 4.3: Could assessment of the outcome have been influenced by knowledge of intervention received?** | NA  NA | NA |
| **4.5 If Y/PY/NI to 4.4: Is it likely that assessment of the outcome was influenced by knowledge of intervention received?** |  | NA |
| **Risk-of-bias judgement** |  | Low |
| Optional: What is the predicted direction of bias in measurement of the outcome? |  | NA |

Domain 5: Risk of bias in selection of the reported result

| **Signalling questions** | **Comments** | **Response options** |
| --- | --- | --- |
| **5.1 Were the data that produced this result analysed in accordance with a pre-specified analysis plan that was finalized before unblinded outcome data were available for analysis?** | No information reported about a protocol or a statistical analysis plan. | NI |
| **Is the numerical result being assessed likely to have been selected, on the basis of the results, from...** |  |  |
| **5.2. ... multiple eligible outcome measurements (e.g. scales, definitions, time points) within the outcome domain?** | No. Outcome reported in “servings per day” (only one measure). | N |
| **5.3 ... multiple eligible analyses of the data?** | Probably no. | PN |
| **Risk-of-bias judgement** |  | Some concerns |
| Optional: What is the predicted direction of bias due to selection of the reported result? |  | NA |

Overall risk of bias

| **Risk-of-bias judgement** | All domains at low risk, except domain 5 due to no statement about a pre-specified analysis plan / reference to such plan or a protocol.  Overall risk of bias therefore judged as at `some concerns`. | Some concerns |
| --- | --- | --- |
| Optional: What is the overall predicted direction of bias for this outcome? |  | NA |

| **Study details**   \| **Reference** \| Tapper, K., Jiga-Boy, G., Maio, G. R., Haddock, G., & Lewis, M. (2014). Development and Preliminary Evaluation of an Internet-Based Healthy Eating Program: Randomized Controlled Trial. *Journal of Medical Internet Research, 16*(10), e231. https://doi.org/10.2196/jmir.3534 \| \| --- \| --- \|   **Study design**   \| X \| Individually-randomized parallel-group trial \| \| --- \| --- \| \| □ \| Cluster-randomized parallel-group trial \| \| □ \| Individually randomized cross-over (or other matched) trial \|   **For the purposes of this assessment, the interventions being compared are defined as**   \| Experimental: \| Intervention \| Comparator: \| Active control condition \| \| --- \| --- \| --- \| --- \|  \| **Specify which outcome is being assessed for risk of bias** \| “6 month” laboratory (meaning 4 months into the intervention) \| \| --- \| --- \|  \| **Specify the numerical result being assessed.** In case of multiple alternative analyses being presented, specify the numeric result (e.g. RR = 1.52 (95% CI 0.83 to 2.77) and/or a reference (e.g. to a table, figure or paragraph) that uniquely defines the result being assessed. \| M.e = 3.9 (SD.e = 1.6)  M.c = 3.3 (SD.c = 1.5) \| \| --- \| --- \|   **Is the review team’s aim for this result…?**   \| x□ \| to assess the effect of *assignment to intervention* (the ‘intention-to-treat’ effect) \| \| --- \| --- \| \| □ \| to assess the effect of *adhering to intervention* (the ‘per-protocol’ effect) \|   **If the aim is to assess the effect of *adhering to intervention***, select the deviations from intended intervention that should be addressed (at least one must be checked):  □ occurrence of non-protocol interventions  □ failures in implementing the intervention that could have affected the outcome  □ non-adherence to their assigned intervention by trial participants  **Which of the following sources were obtained to help inform the risk-of-bias assessment? (tick as many as apply)**  □x Journal article(s) with results of the trial  □ Trial protocol  □ Statistical analysis plan (SAP)  □ Non-commercial trial registry record (e.g. ClinicalTrials.gov record)  □ Company-owned trial registry record (e.g. GSK Clinical Study Register record)  □ “Grey literature” (e.g. unpublished thesis)  □ Conference abstract(s) about the trial  □ Regulatory document (e.g. Clinical Study Report, Drug Approval Package)  □ Research ethics application  □ Grant database summary (e.g. NIH RePORTER or Research Councils UK Gateway to Research)  □ Personal communication with trialist  □ Personal communication with the sponsor |
| --- | --- | --- | --- | --- | --- | --- | --- | --- | --- | --- | --- | --- | --- | --- | --- | --- | --- | --- | --- | --- |

## Risk of bias assessment

Responses underlined in green are potential markers for low risk of bias, and responses in red are potential markers for a risk of bias. Where questions relate only to sign posts to other questions, no formatting is used.

**Domain 1: Risk of bias arising from the randomization process**

| **Signalling questions** | **Comments** | **Response options** |
| --- | --- | --- |
| **1.1 Was the allocation sequence random?** | “[...] stratified block randomization protocol [...]”  Online distribution of intervention/control group information. | Y |
| **1.2 Was the allocation sequence concealed until participants were enrolled and assigned to interventions?** |  | PY |
| **1.3 Did baseline differences between intervention groups suggest a problem with the randomization process?** | No baseline differences (baseline values Int: 3.7 (1.7), Control: 3.6 (1.5).) | N |
| **Risk-of-bias judgement** | Low | Low |
| Optional: What is the predicted direction of bias arising from the randomization process? | NA | NA |

Domain 2: Risk of bias due to deviations from the intended interventions (effect of assignment to intervention)

| **Signalling questions** | **Comments** | **Response options** |
| --- | --- | --- |
| **2.1. Were participants aware of their assigned intervention during the trial?** | Yes “ Although participants were not blind to group allocation, they were informed that [...]”  No (online distribution of information) | Y |
| **2.2. Were carers and people delivering the interventions aware of participants' assigned intervention during the trial?** |  | N |
| **2.3. If Y/PY/NI to 2.1 or 2.2: Were there deviations from the intended intervention that arose because of the trial context?** | No | PN |
| **2.4 If Y/PY to 2.3: Were these deviations likely to have affected the outcome?** | NA | NA |
| **2.5. If Y/PY/NI to 2.4: Were these deviations from intended intervention balanced between groups?** | NA | NA |
| **2.6 Was an appropriate analysis used to estimate the effect of assignment to intervention?** | Yes, intention to treat analysis. | Y |
| **2.7 If N/PN/NI to 2.6: Was there potential for a substantial impact (on the result) of the failure to analyse participants in the group to which they were randomized?** | NA | NA |
| **Risk-of-bias judgement** | Low | Low |
| Optional: What is the predicted direction of bias due to deviations from intended interventions? |  | NA |

Domain 3: Missing outcome data

| **Signalling questions** | **Comments** | **Response options** |
| --- | --- | --- |
| **3.1 Were data for this outcome available for all, or nearly all, participants randomized?** | Yes | Y |
| **3.2 If N/PN/NI to 3.1: Is there evidence that the result was not biased by missing outcome data?** | NA | NA |
| **3.3 If N/PN to 3.2: Could missingness in the outcome depend on its true value?** | NA  NA | NA |
| **3.4 If Y/PY/NI to 3.3: Is it likely that missingness in the outcome depended on its true value?** |  | NA |
| **Risk-of-bias judgement** |  | Low |
| Optional: What is the predicted direction of bias due to missing outcome data? |  | NA |

Domain 4: Risk of bias in measurement of the outcome

| **Signalling questions** | **Comments** | **Response options** |
| --- | --- | --- |
| **4.1 Was the method of measuring the outcome inappropriate?** | No (validated fruit frequency questionnaire) | N |
| **4.2 Could measurement or ascertainment of the outcome have differed between intervention groups?** | No | N |
| **4.3 If N/PN/NI to 4.1 and 4.2: Were outcome assessors aware of the intervention received by study participants?** | Yes, outcome assessor was the participant themselves (self-reported), and they were not blinded. | PY |
| **4.4 If Y/PY/NI to 4.3: Could assessment of the outcome have been influenced by knowledge of intervention received?** | Control group participants probably believe to be in a health intervention. | PY |
| **4.5 If Y/PY/NI to 4.4: Is it likely that assessment of the outcome was influenced by knowledge of intervention received?** |  | PN |
| **Risk-of-bias judgement** |  | some concern |
| Optional: What is the predicted direction of bias in measurement of the outcome? |  | NA |

Domain 5: Risk of bias in selection of the reported result

| **Signalling questions** | **Comments** | **Response options** |
| --- | --- | --- |
| **5.1 Were the data that produced this result analysed in accordance with a pre-specified analysis plan that was finalized before unblinded outcome data were available for analysis?** | No information reported about a protocol or a statistical analysis plan. | NI |
| **Is the numerical result being assessed likely to have been selected, on the basis of the results, from...** |  |  |
| **5.2. ... multiple eligible outcome measurements (e.g. scales, definitions, time points) within the outcome domain?** | No. Outcome reported in “servings per day” (only one measure). | N |
| **5.3 ... multiple eligible analyses of the data?** | Probably no. | PN |
| **Risk-of-bias judgement** |  | Some concerns |
| Optional: What is the predicted direction of bias due to selection of the reported result? |  | NA |

Overall risk of bias

| **Risk-of-bias judgement** | All domains at low risk, except domain 5 due to no statement about a pre-specified analysis plan / reference to such plan or a protocol.  Overall risk of bias therefore judged as at `some concerns`. | Some concerns |
| --- | --- | --- |
| Optional: What is the overall predicted direction of bias for this outcome? |  | NA |
